# Structural and Enzymatic Basis for Impaired Deacylation of *N*‑Acyl‑*N*‑Methyl Lysine Residues

**DOI:** 10.1021/acs.biochem.6c00508

**Published:** 2026-09-08

**Authors:** Sebastian Hilscher, Paul Horst Böttcher, Florian Friedrich, Martin Pannek, Fady Baselious, Marat Meleshin, Oliver Einsle, Cyril Barinka, Clemens Steegborn, Wolfgang Sippl, Manfred Jung, Mike Schutkowski, Cordelia Schiene-Fischer

**Affiliations:** † Department of Medicinal Chemistry, Institute of Pharmacy, 9176 Martin-Luther-University Halle-Wittenberg, Kurt-Mothes-Str. 3, Halle/Saale 06120, Germany; ‡ Department of Enzymology, Charles Tanford Protein Center, Institute of Biochemistry and Biotechnology, 9176 Martin-Luther-University Halle-Wittenberg, Kurt-Mothes-Str. 3a, Halle/Saale 06120, Germany; § Institute of Pharmaceutical Sciences, 9174 University of Freiburg, Albertstr. 25, Freiburg 79104, Germany; ∥ Institute of Biochemistry, 9174 University of Freiburg, Albertstr. 21, Freiburg 79104, Germany; ⊥ Institute of Biotechnology of the Czech Academy of Sciences, BIOCEV, Prumyslova 595, Vestec 252 50, Czech Republic; # Department of Biochemistry, 26523 University of Bayreuth, Universitaetsstr. 30, Bayreuth 95447, Germany

## Abstract

Posttranslational lysine acetylation and methylation are generally considered mutually exclusive modifications of the ε-amino group. The recent discovery of *N*-acetyl-*N*-methyl lysine (KAcMe) on histone H4 showed that both marks can coexist on the same lysine side chain, raising the question of whether this dual modification is enzymatically reversible. Here, we synthesized model peptides containing *N*-acyl lysine or *N*-acyl-*N*-methyl lysine residues and tested their processing by Zn^2+^-dependent HDACs and NAD^+^-dependent sirtuins. While the corresponding unmethylated substrates were deacylated by the expected enzymes, KAcMe-containing peptides were not detectably deacetylated by either enzyme family. *N*-methylation also blocked HDAC8/11-mediated demyristoylation and SIRT5-mediated desuccinylation, whereas SIRT2 and SIRT6 retained weak demyristoylase activity toward *N*-myristoyl-*N*-methyl lysine. X-ray crystal structures of sirtuin–peptide complexes, supported by inhibition studies and molecular docking, reveal that *N*-methylation disrupts productive substrate positioning and misaligns the scissile amide bond for catalysis. These findings suggest that KAcMe is resistant to canonical lysine deacetylases and may represent a persistent chromatin-associated modification.

## Graphical abstract

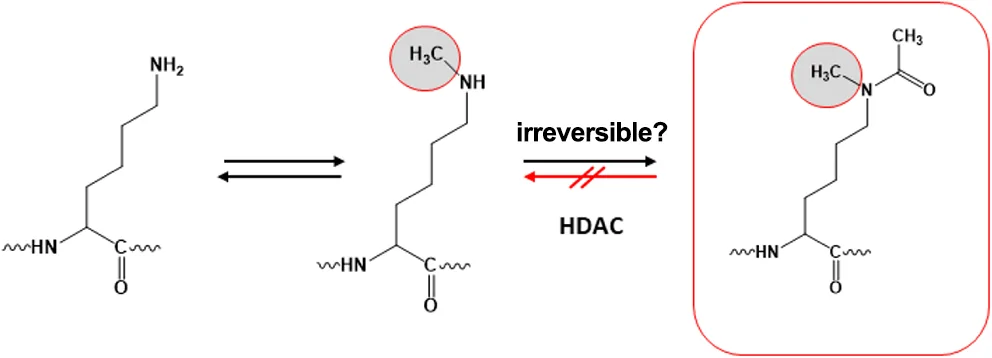

## Introduction

The lysine side chain can undergo a wide variety of posttranslational modifications (PTMs). They include methylation and acetylation, but also other acylations including propionylation, butyrylation, crotonylation, malonylation, succinylation, glutarylation, and β-hydroxybutyrylation, as well as modifications by proteins like ubiquitination or SUMOylation.^1,2^ The lysine PTM status of histones is of major importance for the epigenetic regulation of gene expression by influencing the chromatin structure.^3,4^ In nonhistone proteins, lysine PTMs modulate different properties of the respective proteins, like stability, activity, or subcellular localization.^5^

In terms of PTM crosstalk, different PTMs at one individual site can occur at different times, however, a single PTM is generally considered to establish a covalent bond to one specific atom at a given time. As an exception, up to three methylations may modify incrementally a single nitrogen atom leading to the existence of mono-, di-, or trimethylated lysine side chains or the formation of asymmetric dimethylarginine. Similarly, oxidation of cysteine leads to sulfenic, sulfinic, and sulfonic acids by successive covalent attachment of oxygen to the sulfur atom. However, in both cases, a single atom is modified by more than one of the same groups.

Despite its chemical feasibility, it has just been shown recently that two different functional groups, namely acetylation and monomethylation, are not mutually exclusive at the ε-amino group of a lysine residue. Both modifications have been found to exist simultaneously on the side chain of a single lysine residue forming a dual PTM.^6^ The resulting *N*-acetyl-*N*-methyl lysine (KAcMe) on histone H4 was shown to be associated with active transcription start sites. Thus, uniquely, in KAcMe, one atom of an amino acid side chain is covalently modified by two different functional groups at the same time.

Enzymatic covalent modifications of lysine side chains are generally considered to be reversible reactions. They are generally controlled by two classes of enzymes, the so-called writers and erasers that add and remove the respective PTMs and thus determine the levels of lysine PTMs.^2^ The removal of a PTM by erasers to switch off a signal is a prerequisite for timely control of signaling by the modification. Acetylation of the lysine side chain is regulated by histone acetyltransferases (HATs) as writers, which are counterbalanced by histone deacetylases (HDACs) as erasers.^7,8^ To date, 18 different HDACs have been characterized in humans, which can be divided into four classes based on sequence homology. Classes I (HDAC1–3 and 8) and II further divided into subclasses IIa (HDAC4, 5, 7, and 9) and IIb (HDAC6 and 10), and IV (HDAC11) can be summarized as Zn^2+^-dependent HDACs based on their catalytic mechanism.^9^ Class III HDACs are NAD^+^-dependent enzymes known as sirtuins (SIRTs), comprising SIRT1–7.^10^ Lysine methylation is regulated by lysine methyltransferases (KMTs) as writers and lysine demethylases (KDMs) as erasers.^11^ The lysine demethylases LSD1 and LSD2 both are flavin-dependent monoamine oxidases and only demethylate monomethyl and dimethyl lysine residues.^12^ In contrast, Jumonji C (JMJC) domain-containing proteins use an oxygenase mechanism to demethylate monomethylated, dimethylated, and trimethylated lysine residues.^13,14^ The levels of the dual PTM KAcMe are probably also subject to dynamic regulation as implied by their changes in response to cellular signals and transcriptional activity.^6^ The known reaction mechanisms of KMTs and HATs suggest that an initial monomethylation of lysine is followed by acetylation to form KAcMe in a sequential mode of action. Accordingly, the HAT p300 has been described to be able to acetylate *N*-methyl lysine.^6^

Enzyme-mediated deacetylation of KAcMe modifications leading to the formation of *N*-methyl lysine residues was suggested by *in vivo* inhibition experiments.^6^ On the other hand, an additional methyl group at the scissile amide bond of *N*-acetyl lysine residues has been described to convert substrates of Zn^2+^-dependent HDACs into inhibitors that are stable against enzymatic deacetylation.^15^

The aim of this study was to systematically analyze the ability of different HDACs to deacetylate KAcMe residues. Here we show that neither Zn^2+^-dependent HDACs nor sirtuins can remove the acetyl residue from KAcMe in model peptides *in vitro*, suggesting that the presence of the methyl group renders a per se reversible acetylation irreversible. *N*-Methylation also blocked HDAC8/11-mediated demyristoylation and SIRT5-mediated desuccinylation, whereas SIRT2 and SIRT6 retained weak demyristoylase activity toward *N*-myristoyl-*N*-methyl lysine.

## Materials and Methods

### Synthesis

#### Chemicals and General Methods for Peptide Synthesis

All Fmoc-protected amino acids (except lysine) were purchased from GL Biochem Ltd. (Shanghai, China). Fmoc-Lys­(Ns)-OH was prepared as described elsewhere.^16^ *N,N*-dimethylformamide (DMF), piperidine, OxymaPure, pentafluorophenol, and Rink amide MBHA resin were purchased from Iris Biotech (Germany). *N,N*-diisopropylethylamine (DIPEA) was purchased from Carl Roth (Germany). All other chemicals were purchased from Sigma-Aldrich if not stated otherwise. Purification of peptides was done on a Shimadzu LC system with a Phenomenex Kinetex 5 μm XB-C18 (250 × 21.1 mm, 100 Å) column using different gradients of 0.1% trifluoroacetic acid (TFA) in H_2_O (solvent A) and 0.1% TFA in ACN (solvent B) solutions. The wavelength used was 220 nm. UPLC-MS analysis was performed using a Waters ACQUITY UPLC-MS system (Milford, USA) with a Waters ACQUITY-UPLC-MS-BEH C18, 1.7 μm (2.1 × 50 mm; 30 Å) column. As the mobile phase, 0.1% formic acid in H_2_O (solvent A) and 0.1% formic acid in acetonitrile (ACN) (solvent B) solutions were used. A typical gradient from 95:5 (v/v) H_2_O:ACN to 5:95 (v/v) H_2_O:ACN in 6 min was used for most of the runs. Data analysis was performed using Waters MassLynx software.

#### Solid-Phase Peptide Synthesis

The peptides were synthesized using Fmoc-SPPS on Rink Amide resin using an automated microwave peptide synthesizer Liberty Blue (CEM Corporation, Matthews, NC, USA). The coupling of amino acids was performed with DIC/OxymaPure at 90 °C for 2 min, and each coupling step was performed twice. Fmoc deprotection was accomplished with 20% piperidine in DMF at 90 °C for 1 min. Once the peptide had been modified as desired, it was cleaved from the resin using a mixture of TFA/H_2_O/1-dodecanethiol (97:1:2 v/v/v) for 1 h twice.

#### Peptide Modification

Subsequent to the SPPS, the nosyl protecting group was cleaved and the lysine side chain was modified on solid support. Removal of the nosyl group was done by adding β-mercaptoethanol (10 equiv) and DBU (5 equiv) in DMF and incubating for 1 h. For acetylation, a solution containing acetic anhydride (4 equiv) and DIPEA (5 equiv) in dry DMF was used and the reaction was allowed to proceed for 30 min while shaking. For trifluoroacetylation, a solution of ethyl trifluoroacetate (5% v/v) and DIPEA (5% v/v) in DMF was added to the resin and incubated overnight. For succinylation, a solution of succinic anhydride (4 equiv) and DIPEA (5 equiv) in dry DMF was added for 30 min. For fatty acylation, a solution of the corresponding fatty acid (5 equiv), DIPEA (5 equiv), and HATU (5 equiv) in dry DMF was added and allowed to react for 1 h. The reaction was repeated once. For alkylation, the alkyl groups were introduced in the presence of the nosyl group. The methyl, propyl, pentyl, and benzyl groups were introduced by adding a solution of triphenylphosphine (5 equiv) and the corresponding dry alcohol (10 equiv) in dry THF. After 5 min, a solution of diisopropyl azodicarboxylate (DIAD) (5 equiv) in dry THF was added and the reaction was left shaking for 24 h. Isopropylation and neopentylation were performed by addition of acetone or pivalaldehyde (10 equiv) in dry DMF with 1% (v/v) acetic acid, followed by NaBH_3_CN (10 equiv) in dry DMF and shaking for 48 h. UPLC-MS analysis of the synthesized compounds was performed (Table S1, Figures S1–S22).

### Expression and Purification of Recombinant Proteins

For HDAC1, 2, 3, and HDAC6, the recombinant proteins were purchased from Enzo Life Sciences AG (Lausen, Switzerland), whereas HDAC4, 5, and 7–11 were produced as described.^15,17^ Sirtuins 2, 3, 5, and 6 were expressed and purified as described.^18^

Human SIRT1 (143–747) with an *N*-terminal His_6_-SUMO tag was obtained by expression from a peSUMO vector in *E. coli* BL21 (DE3) in LB medium supplemented with ampicillin or kanamycin. Overexpression was induced by the addition of IPTG at a final concentration of 0.5 mM at an OD_600_ of 0.8 at 18 °C. Cells were harvested by centrifugation, resuspended in lysis buffer (50 mM Tris, pH 7.8, 0.25 M NaCl, 1 mM β-mercaptoethanol), and lysed by sonication. Cell debris was pelleted by ultracentrifugation and the resulting supernatant was loaded onto a Ni chelating sepharose column (GE Healthcare, Uppsala, Sweden). The loaded column was intensively washed before elution with 200 mM imidazole in 50 mM Tris, pH 7.8, 0.25 M NaCl, 1 mM β-mercaptoethanol. Sirtuin-containing fractions were concentrated and finally separated using a Superdex 200 16/600 gel filtration column (GE Healthcare) equilibrated with 25 mM Tris, pH 7.6, 300 mM NaCl, 1.5 mM DTT.

### Enzymatic Assays

#### HPLC-MS-Based Deacylation Assays

The *in vitro* deacylation of the HDACs was monitored using UPLC-MS. The assays were performed in the following buffers: HDAC buffer (HDAC1–9) contained 20 mM HEPES, 140 mM NaCl, 10 mM KCl, 0.1 mg/mL BSA, 1 mM TCEP, pH 7.4; HDAC10 buffer consisted of 20 mM HEPES, 0.1 mg/mL BSA, pH 7.4; HDAC11 buffer contained 20 mM HEPES, 0.5 mg/mL BSA, 70 μM TCEP, pH 7.4; and the SIRT buffer contained 20 mM Tris, 150 mM NaCl, 5 mM MgCl_2_, 0.1 mg/mL BSA, 50 μM TCEP, 1 mM NAD^+^, pH 7.8.

Peptide derivatives were analyzed for their substrate properties at concentrations of 50 μM for the unmethylated and 100 μM for the methylated derivatives. Product formation was quantitatively evaluated by the product peak area in the photometric analysis of the UPLC separation. Conversion of less than 1% after a 24 h reaction with the respective HDAC was considered as not detectable by evaluation of UPLC-MS as assessed by analysis of a series of different concentrations of product peptide **2**. Absence of the formed product was confirmed by mass spectral scans for the presumed cleavage product.

The demyristoylation of peptides **5** and **6** by SIRT2 and SIRT6 was monitored using HPLC. SIRT2 and SIRT6 (0.5–2.0 μM) were incubated for different times at 30 °C in SIRT buffer with the substrate peptide (15 μM) followed by analyzing demyristoylation activity by HPLC separation. Analytical runs were performed on an Agilent 1100 system (Boeblingen, Germany). Separation was done with a linear water/ACN gradient within 6 min and a flow rate of 0.5 mL/min on a 3.0 × 50 mm reversed-phase column (Phenomenex Kinetex XB-C18, 2.6 μm). Absorbance was monitored at 280 nm. Product formation was calculated as the ratio of product peak area to total peak area.

To control the activity of the respective HDACs independently, the following standard substrates optimal for the individual HDAC tested were used: the acetylated p53-derived peptide substrate Ac-RHKK­(Ac)-AMC for HDAC1–3;^19^ the trifluoroacetylated artificial peptide **S1** for HDAC4–9 and 11;^15,17^ the myristoylated TNFα-derived peptide substrate Ac-EALPKK­(Myr)­TGG-NH_2_ for SIRT2, 3, and 6;^16^ the acetylated TNFα-derived peptide substrate Ac-EALPKK­(Ac)­TGG-NH_2_ for SIRT1; and the acetylated CPS1-derived peptide substrate Bz-GVLK­(Suc)­EYGV-NH_2_ for SIRT5.^20^ HDACs 1–11 (50 nM), SIRT2, 3 (300 nM), and SIRT1, 5, and 6 (2 μM) were incubated for 24 h at 30 °C in the corresponding buffer with the peptide derivative to check for deacetylation activity. In the control reactions, buffer conditions and DMSO concentrations corresponding to the deacylation experiments for the dually modified peptides and their unmethylated counterparts were used.

#### Fluorescence-Based Inhibition Assays

Inhibition of HDACs was analyzed in fluorescence-based enzymatic in vitro assays using 384-well plates in a PerkinElmer EnVision 2104 multilabel plate reader. After 5 min of incubation of the compounds with the respective enzymes (HDAC1 = 10 nM, HDAC2 and HDAC3 = 3 nM, HDAC4 = 5 nM, HDAC5 = 10 nM, HDAC6 = 1 nM, HDAC7 = 5 nM, HDAC8 = 2 nM, HDAC9 = 20 nM, HDAC11 = 20 nM), the reactions were started by the addition of substrate.

For HDAC1, 2, 3, and 6, an acetylated peptide substrate derived from p53 (Ac-RHKK­(Ac)-AMC) was used in a discontinuous assay.^15^ All reactions were performed in assay buffer (20 mM HEPES, 140 mM NaCl, 10 mM MgCl_2_, 1 mM TCEP, and 0.2 mg/mL BSA, pH 7.4, adjusted with NaOH) at 37 °C. The reaction was quenched after 1 h by adding trypsin and suberoylanilide hydroxamic acid (SAHA). The fluorescence intensity was measured after 1 h of incubation with an excitation wavelength of 380 nm and an emission wavelength of 430 nm. HDAC4, 5, 7, 8, and 9 were measured in a continuous manner using the thiotrifluoroacetylated artificial peptide substrate Abz-SRGGK­(TFAc)­FFRR.^15^ The fluorescence increase was followed for 1 h with two reads per min with an excitation wavelength of 320 nm and an emission wavelength of 430 nm. HDAC11 activity was measured in a continuous manner using the peptide substrate Ac-EALPKK­(11-Abz-Aun)­Y­(NO_2_)­GG-NH_2_,^21^ inhibition of HDAC11 was analyzed as described.^22^ Positive (enzyme, substrate, DMSO, and buffer) and negative (substrate, DMSO, and buffer) controls were included in every measurement and were set as 100% and 0%, respectively, and the measured values were normalized accordingly. All tests were done in three replicates. If no IC_50_ value could be determined in the concentration range used, an inhibition assay with 100 μM inhibitor was performed to determine possible binding.

### Crystal Structure Analyses

#### Production of Zebrafish SIRT5

The expression and purification of zebrafish SIRT5 (zSIRT5) were performed as described previously.^20^

#### Crystallization of zSIRT5/Peptide Complexes

The zSIRT5 protein (10 mg/mL in 20 mM Tris/HCl pH 8.5, 150 mM NaCl) was cocrystallized with 1 mM KSucMe CPS1-peptide (final DMSO concentration, 10%) at 20 °C using 18–24% PEG 3350, 0.1 M HEPES, pH 7.0 to 8.0 as the reservoir solution. Crystallization drops were set up at 2:1, 1:1, and 1:2 ratios of protein:reservoir and crystals grew in a rod-shaped form with a length of 100–300 μm and a maximum diameter of 50 μm within 2 weeks. Prior to diffraction experiments, crystals were transferred to a cryoprotectant solution composed of the reservoir solution and ligand peptide supplemented with 25% glycerol, and they were subsequently shock-frozen in liquid nitrogen. Diffraction data were collected on BL14.1 operated by the Helmholtz-Zentrum Berlin (HZB) at the BESSY II electron storage ring (Berlin-Adlershof, Germany).^23^ Indexing, scaling, and merging of diffraction data were done using XDS.^24^ Structures were solved by molecular replacement using MolRep^25^ using a previously solved zSIRT5/CPS1-peptide complex (PDB entry 4UTV^20^) as a search model. Refinement was performed with REFMAC5 ^26^ using local NCS restraints and TLS refinement.^27^ Manual model building and structure validation was done using Coot;^28^ acyl-lysine restraints were generated by using the PRODRG server, and figures were created using PyMOL.^29^

#### SIRT2 Expression and Purification for Crystallization and Soaking

For crystallization experiments, human SIRT2_56–356_ was expressed and purified as previously described with minor modifications summarized hereafter.^30^ Cell cultures were grown in 2× YT medium supplemented with 100 μg/mL ampicillin at 37 °C reaching an OD_600_ of 0.6–0.8. Protein expression of His_10_-tagged SIRT2 was induced with 1 mM IPTG, followed by incubation for 16 h at 20 °C. The His_10_-Tag was cleaved via TEV protease in lysis buffer (50 mM Tris/HCl, 500 mM NaCl, 10% (v/v) glycerol, 0.5 mM TCEP, pH 8.0) at 4 °C for 18–24 h. Cleaved protein was isolated using a HisTrap HP column (5 mL, GE Healthcare), by collecting the flow-through. A final purification step via size-exclusion chromatography (Superdex S75 26/600) in SEC buffer (20 mM HEPES, 150 mM NaCl, pH 7.5) yielded pure SIRT2. The protein was concentrated, flash-frozen, and stored at −80 °C. Protein purity and identity were confirmed by SDS-PAGE, and protein concentration was determined using the BCA assay with BSA as a standard.

Crystallization trials were set up using an Oryx Nano pipetting robot (Douglas Instruments, East Garston, UK) and the sitting-drop vapor-diffusion method (MRC 2-Well UVP Plate, SWISSCI, Buckinghamshire, UK) at 20 °C. SIRT2_56–356_ was mixed with compound **12** and incubated on ice for 1 h prior to crystallization, then cleared by centrifugation for 10 min at 4 °C to remove precipitates.

Crystals of the SIRT2–**12** complex (11.6 mg/mL SIRT2, 1.25 mM compound **12**; 2.5% v/v DMSO) appeared within 1 day from drops composed of 0.2 μL protein solution and 0.4 μL reservoir solution containing 21.5% (w/v) PEG 3350 and 0.1 M Bis–Tris (pH 6.5). Crystals were cryoprotected with a mixture of reservoir solution and 10% (v/v) 2*R*,3*R*-(−)-butanediol, mounted on nylon loops, and flash-cooled in liquid nitrogen.

The SIRT2–**12**–NAD^+^ ternary complex was obtained by soaking NAD^+^ into preformed SIRT2–**12** crystals (11.6 mg/mL SIRT2, 1.25 mM compound **12**; 2.5% v/v DMSO) grown in 21.5% PEG 3350 and 0.1 M HEPES (pH 7.5). For soaking, compound **12** (50 mM, in DMSO) and NAD^+^ (100 mM, in SEC buffer) were diluted 1:10 into a solution containing 31.5% (w/v) PEG 3350 and 0.1 M HEPES (pH 7.5), resulting in final concentrations of 5 mM for compound **12** and 10 mM for NAD^+^. Crystals were incubated in this solution for 6 h, then cryoprotected with the reservoir solution supplemented with 10% (v/v) DMSO, mounted on nylon loops, and flash-cooled in liquid nitrogen.

#### Data Collection and Structure Determination

X-ray diffraction data for the SIRT2–**12** (PDB 30KD) complex were collected on the ID30A-3 (MASSIF-3) beamline at the European Synchrotron Radiation Facility (ESRF, Grenoble, France) using an EIGER1 × 4 M detector (DECTRIS, Switzerland). X-ray diffraction data for the SIRT2–**12**–NAD^+^ (PDB 30KE) complex were collected on the ID23-2 beamline at the ESRF using an EIGER2 × 9 M detector (DECTRIS, Switzerland). The data sets were processed with autoPROC^31^ and scaled using Aimless.^32^ The structures were solved by molecular replacement with Phaser^33^ using the SIRT2 apo structure (PDB 9FDR)^34^ and the SIRT2–H3K9myr complex (PDB 4Y6L)^35^ as the search models. Models were built and refined iteratively in COOT^28^ and either REFMAC^36^ or Phenix.refine.^37^ Restraints for **12** were generated with the grade Web Server (Global Phasing Ltd., UK). Final structures were validated using MolProbity.^38^ All data collection and refinement statistics are reported in Table S2.

### Computational Methods

For the molecular docking study, Schrödinger Suite 2025-2 was used. LigPrep panel^39^ with OPLS2005 ^40–46^ force fields was used to prepare the substrates. The optimized HDAC11-AF2 model bound to SIS17 in the vertical pose was utilized as the receptor.^47^ To generate a suitable grid for substrate docking, the HDAC11 model was merged with a water molecule and a lysine residue derived from the HDAC8 crystal structure complexed with a substrate (PDB 2V5W^48^). The merged complex was then minimized using Desmond minimization with OPLS5. For this, the complex was solvated in an orthorhombic box using the SPC water model and a buffer distance of 10 Å. The system was neutralized by adding chloride ions 4 Å away from the bound ligand.

Receptor grids were generated using the receptor grid generation panel by utilizing the centroid of the ligand. The Glide module^49–51^ was used for docking with flexible ligand sampling and standard precision using the OPLS2005 force field.

For docking of H4K5Myr peptide **5** and the artificial KAc peptide **S2,** a fragment of *N-*myristoylated lysine was docked using core comparison constraints specifying the core atoms with the maximum common substructure. The docked fragment was used to generate a new grid as described above while increasing the length of ligands to be docked to 35 Å and increasing the inner box size to 15 Å to account for the difference in ligand size.

For docking of H4K5MyrMe peptide **6** and the artificial KAcMe peptide **10**, a fragment of *N*- methylated, *N*-myristoylated lysine was aligned flexibly to the *N*-myristoylated lysine in the HDAC11 model using the Ligand Alignment Panel. The receptor was then minimized in the presence of the new fragment using the Protein Preparation Panel^52,53^ specifying all atoms with a cutoff of 0.3 Å, and the grid was generated as mentioned above.

Different conformers of the substrates were generated using the ConfGen application.^54^ The generated conformers were subjected to core-constrained docking using Glide by applying enhanced sampling and subjecting 100 poses to postdocking minimization while reporting 5 poses. Docked poses were then clustered using the Conformer Cluster Panel by atomic RMSD using average linkage to generate 20 clusters for each substrate. The members of each cluster were arranged according to their Glide score, and the top-scoring member was selected as the representative of the cluster. Selected poses of the substrates were compared by an RMSD distance matrix, and similar poses (with the lowest RMSD) were selected for further analysis.

## Results

### Synthesis of Model Peptides Containing Dually Modified Lysine Residues

Dual modification of the lysine residue by an acetyl and a methyl residue KAcMe was found at H4K5.^6^ To analyze the substrate properties of acyl residues in the additional presence of a methyl residue at the same lysine ε-amino group for enzymatic deacylation by HDACs, the 11-mer peptide Ac-SGRGKGGKGWW-NH_2_ **1** representing positions −4 to +4 of H4K5 was used. The two additional tryptophans at the C-terminus were introduced for efficient peptide detection by UV-Vis. We synthesized derivatives of this sequence carrying an acetyl (**3**, **4**), myristoyl (**5**, **6**), or succinyl (**7**, **8**) group as the acyl moiety either in combination with a methyl group (**4**, **6**, **8**) or a hydrogen atom (**3**, **5**, **7**) at the side-chain amino group of the central lysine (Figure 1). Peptides were prepared using automated Fmoc-based solid-phase peptide synthesis (SPPS). The lysine side chain selected for modification was protected with a nosyl group. The alkyl groups were introduced after SPPS by treating the resin-bound peptide with the corresponding alcohol under Mitsunobu conditions. Acylations were performed after removal of the nosyl group. Peptides **1** and **2** contain no acyl residue at the central lysine, thus representing the potential products of a deacylation reaction. To get access to the enzymatic activity of the catalytically less active class IIa HDACs, the artificial sequence SRGGKFFRR already described as the basis for very good HDAC substrates^55^ and the trifluoroacetyl group which is readily hydrolyzed from the lysine side chain^56^ were used to obtain peptide derivatives **S1** and **9**. The *N*-terminal Abz group allows fluorescence detection in the HPLC-based activity assay. The lysine of **9** is modified with a methyl group in addition to the trifluoroacetyl group (KTFAcMe). Peptide **10** with the *N*-terminal nonfluorescent Bz group allowed enzyme binding analysis at higher peptide concentrations. **S2** is the unmethylated counterpart of **10** (Figure 1).

**Figure 1:**
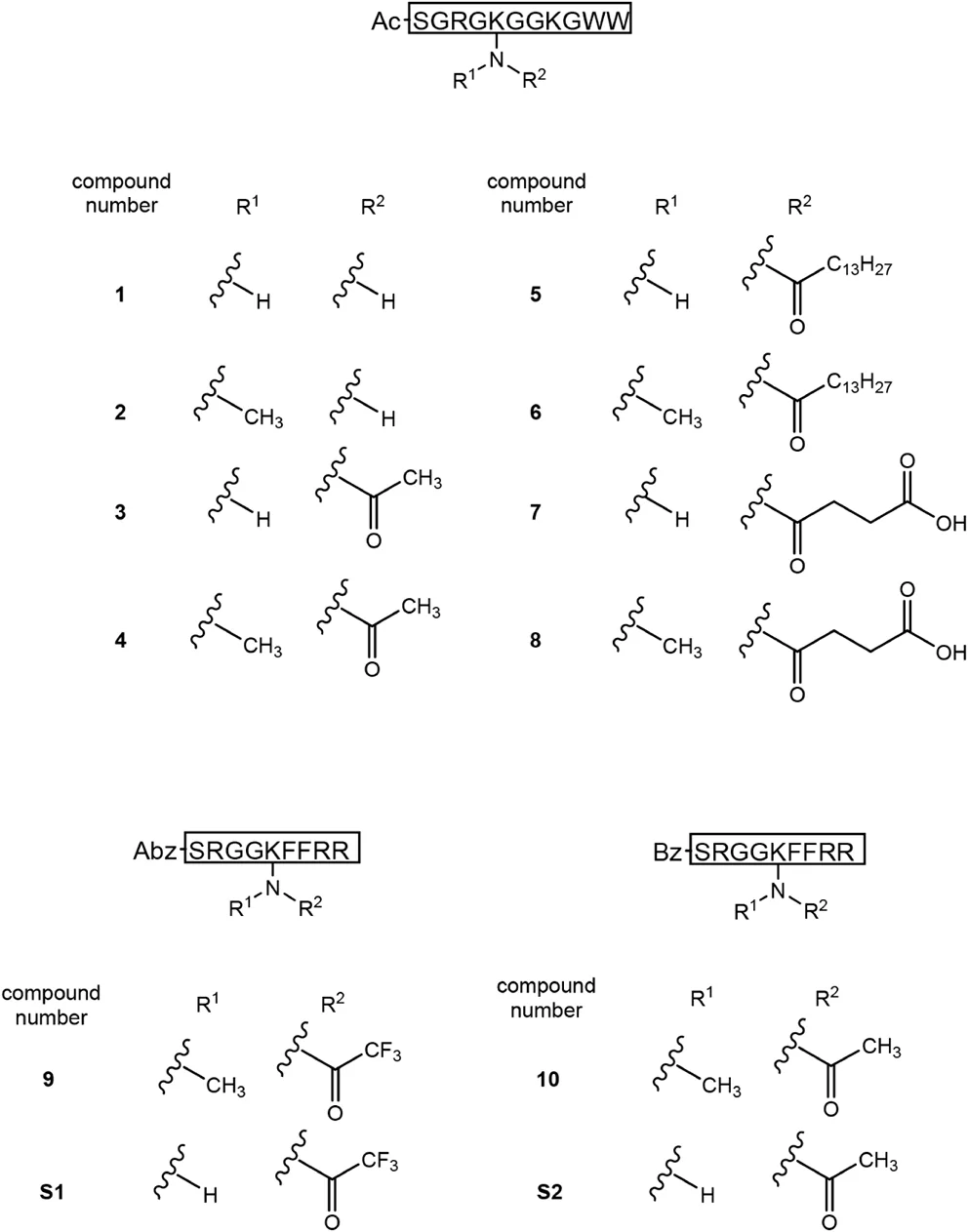
Schematic representation of the peptides **1**–**10**, **S1,** and **S2** analyzed in this study.

### Zn^2+^-Dependent HDACs Are Unable to Deacylate Dually Modified Lysine Residues

Initially, we analyzed the ability of the different Zn^2+^-dependent HDACs to use peptides based on the H4K5 sequence as substrates. After incubation of the respective isozyme at 50 nM with 50 μM of **3** for 3 h at 30 °C, UPLC-MS analysis was performed. It was found that HDACs 1–3 and 6 were able to deacetylate the H4K5Ac peptide **3** (Table 1), whereas the other HDACs were not able to use **3** as a substrate. HDAC10 was omitted from the analysis because it is a poor lysine deacylase but rather recognizes acetylated polyamines as substrates.^57^ To analyze if a deacylation reaction is catalyzed by the respective HDAC also in the presence of an additional methyl group at the ε-amino group of the lysine, 50 nM of HDAC1–3 or 6 were incubated with 100 μM of the H4K5AcMe peptide **4** for 24 h at 30 °C. No deacetylation could be detected (Table 1, Figures S23–S26, S38). Since HDAC8 and HDAC11 are known to exhibit activity against long-chain acyl groups,^17,58,59^ for these enzymes demyristoylation of the H4K5Myr peptide **5** was analyzed after incubation of the respective isozyme with **5** for 24 h at 30 °C. Both HDAC8 and 11 were found to be able to demyristoylate **5** (Table 1). However, an additional methyl group at the amino group of the lysine residue prevented demyristoylation. After incubation of 50 nM of HDAC8 or HDAC11 with 100 μM of the H4K5MyrMe peptide **6** for 24 h, no product of demyristoylation could be detected (Table 1, Figures S31, S32, S39). As already described, class IIa HDACs are known to be inert toward acetyl lysine residues even in the optimized artificial HDAC substrate peptide, whereas trifluoroacetyl lysine peptides are readily hydrolyzed by these HDACs.^15,56^ Thus, the artificial *N-*trifluoroacetyl *N-*methyl lysine peptide **9** in comparison to the artificial *N-*trifluoroacetyl lysine peptide **S1** as a standard substrate was used to analyze the enzymatic activity of HDACs 4, 5, 7, and 9.^15^ Reactions of the unmethylated peptide derivative **S1** were performed for 1 h, whereas peptide **9** was incubated for 24 h with the respective HDAC. Clearly, the dually modified peptide lacks HDAC substrate properties (Table 1).

**Table 1:** HDAC Activity for Unmethylated and Methylated Peptide Derivatives^a^

HDAC isoform	**3** H4K5Ac^a)^	**4** H4K5AcMe^b)^
HDAC1	+	-
HDAC2	+	-
HDAC3	++	-
HDAC6	+++	-
HDAC isoform	**5** H4K5Myr^b)^	**6** H4K5MyrMe^b)^
HDAC8	++	-
HDAC11	+++	-
HDAC isoform	**S1** Abz-SRGGK(TFAc)FFRR^c)^	**9** Abz-SRGGK(TFAcMe)FFRR^b)^
HDAC4	+++	-
HDAC5	+++	-
HDAC7	++	-
HDAC9	+++	-

a The enzyme concentration was 50 nM, the peptide concentration was 50 μM for **3, 5**, and **S1** and 100 μM for **4, 6,** and **9**. Cleavage was analyzed using UPLC-MS. Activity values reflect substrate conversion based on the intensity of absorbance at 280 nm in the photometric analysis of UPLC-MS data (n = 3). signals: +, <10% conversion; ++, 11–60% conversion; +++, 61–100% conversion after ^a)^3 h, ^b)^24 h, and ^c)^1 h. Incubation times were adjusted according to substrate performance. −, no product detectable by MS.

In order to analyze if the peptides with an additional *N-*methyl residue at the *N-*acetylated or *N-*myristoylated lysine are still able to bind to the enzyme’s active site, inhibition experiments were performed (Table 2). To this end, peptides **4** and **6**, representing the doubly modified H4K5 sequence, were tested for their inhibitory potential. Additionally, the doubly modified nonfluorescent artificial peptide **10** was used.

**Table 2:** Inhibition of Zn^2+^-dependent HDACs by Peptide Derivatives **4, 10,** and **6** ^a^

	IC_50_ (μM)		
HDAC-Isoform	**4**H4K5 AcMe	**10**Bz-SRGGKFFRR-NH_2_ AcMe	**6**H4K5MyrMe
HDAC1	ni	ni	nd
HDAC2	ni	ni	nd
HDAC3	ni	5.2%	nd
HDAC4	ni	5.6%	nd
HDAC5	ni	11.4%	nd
HDAC6	59 μM	16.3%	nd
HDAC7	ni	4.2%	nd
HDAC8	ni	6.5%	34.5%
HDAC9	ni	9.9%	nd
HDAC11	ni	8.2 μM	17.2 μM

a The peptides were used in concentrations between 20 nM and 60 μM. If the determined IC_50_ was above 60 μM an inhibition test at [peptide] = 100 μM was performed (% inhibition). n = 3. ni = no inhibition, nd = not determined.

The different inhibition patterns of the different HDACs indicate that acceptance of the additional methyl group in the HDAC active site was influenced by the peptide sequence context in a way specific to each HDAC isoform. For HDACs 1–5 and HDACs 7–9, the affinities of the peptides analyzed were too low to determine IC_50_ values. Peptide **4** was found to bind only to HDAC6 with an IC_50_ value of 59 μM but not to the other HDACs. Peptide **10** significantly inhibited HDAC11 with an IC_50_ value of 8.2 μM. In addition, the inhibitory potential of **6** against HDAC8 and 11 already described to be able to accommodate long-chain acyl groups was evaluated. HDAC11 was found to be potently inhibited by **6** with an IC_50_ value of 17.2 μM. This reflects an increased affinity compared to the unmethylated peptide **5** with an IC_50_ value of 81.4 μM. Determination of the IC_50_ value was performed using a fluorescence-based HDAC11 inhibition assay in the presence of 6 to 200 μM **5** and 20 nM HDAC11. Short-time measurements for 20 min allowed evaluation of the inhibitory potency of **5** despite it being a substrate of HDAC11.

For HDAC8, only moderate inhibition of 34.5% at 100 μM of peptide **6** was found. Thus, *N*-methylation of *N*-acyl lysine causes either (i) abolition of binding to the respective HDAC or (ii) perturbation of a chemical step of catalysis, depending on the HDAC isoform and the sequence of the peptide.

Since the HDAC11 binding site turned out to be quite promiscuous, we used an inhibition approach to further analyze the spatial requirements for efficient binding of peptides containing a lysine residue dually modified by different acyl and alkyl residues. To this end, a series of 7-mer H3K9 peptide derivatives (Ac-TARKSTG-OH) were synthesized, as H3K9 has been documented as a natural deacylation site for HDAC11.^60^ At the side-chain amino group of the central lysine, a range of alkyl modifications in combination with fatty acyl residues of different chain lengths has been introduced yielding compounds **11**–**22**. The inhibitory potential of the compounds for HDAC11 was determined (Table 3, Figures S36, S37).

**Table 3:** IC_50_ Values of Various Dually Alkylated/Acylated Peptides Based on the H3K9 Sequence Were Determined Using a Fluorescence-Based Assay^a^ ^16^

Compound	Alkyl residue	Acyl residue	IC_50_ (μM)
**11**	Me	Lau	>60
**12**	Me	Myr	19.0 ± 2.2
**13**	Me	Pal	28.5 ± 5.4
**14**	Me	Stea	>60
**15**	Et	Myr	52.5 ± 7.4
**16**	Et	Pal	13.7 ± 5.4
**17**	n-Prop	Pal	4.7 ± 0.7
**18**	iso-Prop	Pal	23.3 ± 3.0
**19**	*n*-Bu	Pal	12.2 ± 1.8
**20**	n-Pent	Pal	29.0 ± 4.6
**21**	Bn	Pal	16.9 ± 3.8
**22**	neo-Pent	Pal	57.1 ± 5.2

a [peptide] was 60 μM–20 nM, [HDAC11] was 20 nM, [substrate] was 20 μM. n = 3.

It turned out that all peptide derivatives investigated except for **11** and **14** were able to bind to the HDAC11 active site, as inhibition was detected. In combination with an *N-*methyl residue, *N-*myristoylation of lysine induced the highest inhibitory potential of the 7-mer H3K9 peptide as indicated by an IC_50_ value of 19 μM for compound **12**. As the steric demand for the alkylation increased, palmitoylation emerged as beneficial for HDAC11 binding, as evidenced by a comparison of **15** and **16**.

It should be noted that in **17,** as the most potent inhibitor in this series with an IC_50_ in the lower micromolar range, the lysine is modified by a propyl residue beside a palmitoyl residue indicating a rather large alkyl-binding site of HDAC11. Even sterically demanding alkyl residues, such as the benzyl residue (**21**) and the neopentyl residue (**22**), can be accommodated. However, the increased size of the alkyl residue found in **18**–**20** as well as the use of branched alkyl residues as exemplified by comparing **17** and **18** or **20** and **22** reduced affinity for HDAC11.

To get insight into the molecular basis for the diminished catalytic activity, docking studies were utilized to analyze HDAC11 binding to the H4K5MyrMe peptide **6** and to the artificial KAcMe peptide **10**, which bound with relatively high affinity but no enzymatic catalysis was found to occur (Figure 2). The binding mode of the myristoylated or acetylated, additionally methylated peptides was compared to the analogous peptides without methylation **5** and **S2**, respectively. The binding modes were studied by molecular docking using an optimized AlphaFold2 model of HDAC11. The model was extensively validated and employed to study the binding mode of HDAC11 inhibitors in previous work^47^ and was further utilized to identify and design novel selective inhibitors.^22,61^ The obtained docking poses of the peptides revealed that the carbonyl oxygen of the acetyl/myristoyl group coordinated the Zn^2+^ ion while forming a hydrogen bond with Y304. For the unmethylated substrates **5** and **S2**, the NεH is positioned within a suitable distance to form a hydrogen bond with G151. The myristoyl chain of peptides **5** and **6** is conveniently accommodated in the foot pocket and the extended hydrophobic tunnel of HDAC11 along loop 3 and loop 7, while forming hydrophobic interactions with residues lining the tunnel such as F37, W42, F141, and F152. Comparing the docking poses of **5** (KMyr)/**6** (KMyrMe) and **S2** (KAc)/**10** (KAcMe) revealed that additional lysine *N-*methylation of the peptide derivatives results in the loss of possible hydrogen-bond formation with G151 as well as a slight shift in the positioning of the lysine Nε, which might justify the lack of deacylation of the dually modified peptides.

**Figure 2:**
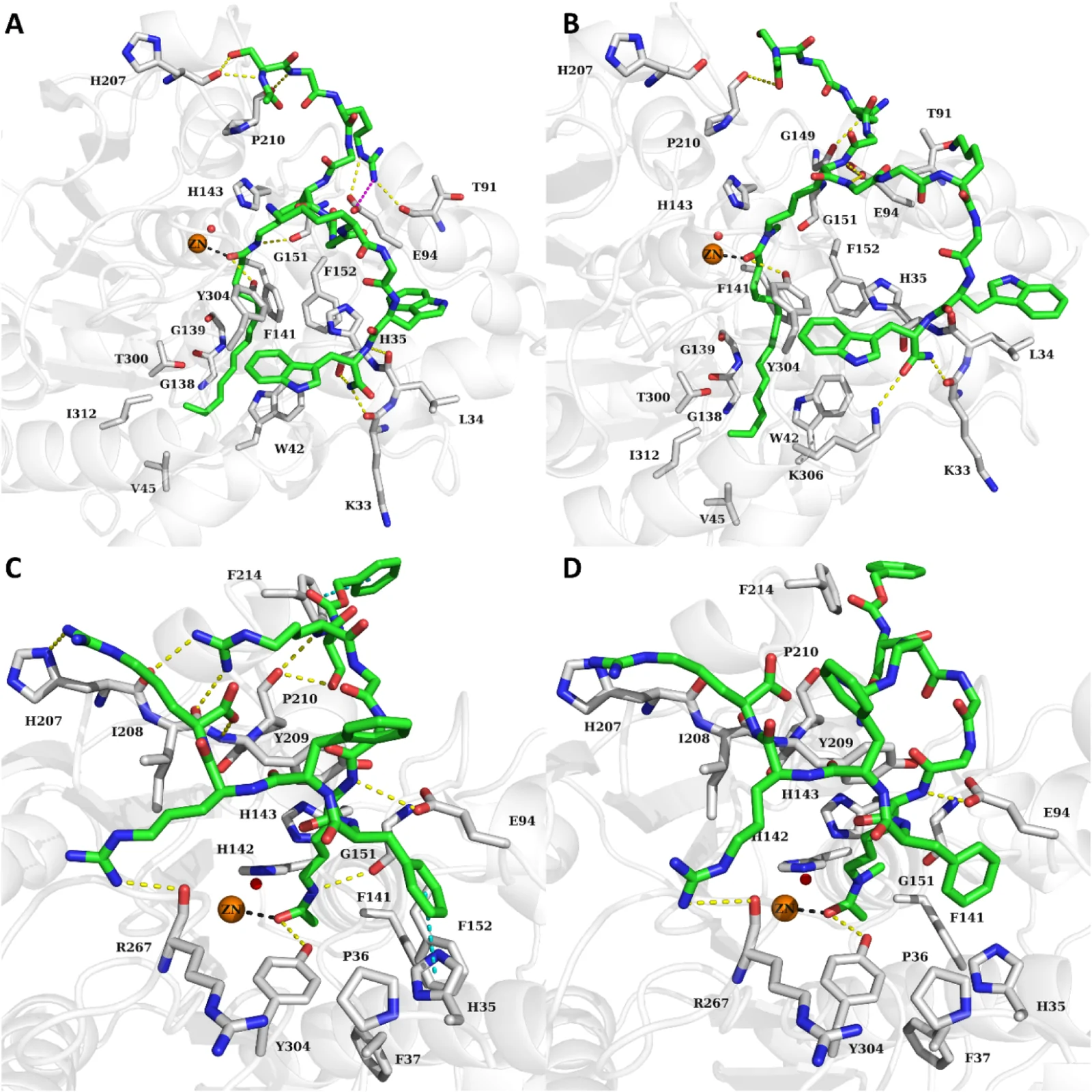
Docking poses of the peptide derivatives in the optimized HDAC11 AlphaFold2 model. (A) H4K5Myr peptide **5**. (B) H4K5MyrMe peptide **6**. (C) Artificial KAc peptide **S2**. (D) Artificial KAcMe peptide **10**. The protein backbone is represented as a white cartoon, the binding site residues are represented as white sticks, the ligands as green sticks, the Zn^2+^ ion as an orange sphere, and water residues as red spheres. The coordination bonds to the Zn^2+^ ion are shown as gray dashed lines, the hydrogen bonds as yellow dashed lines, the Pi–Pi interactions as cyan dashed lines, and the salt bridges as magenta dashed lines.

### Sirtuins Are Unable to Deacetylate and Desuccinylate Dually Modified Lysine Residues

Next, we examined the ability of sirtuins to deacylate dually modified lysine residues. We first analyzed the substrate properties of H4K5-based peptide derivatives lacking additional methylation and carrying different *N-*acyl modifications at the lysine with respect to the major sirtuins. Sirtuins SIRT1–3 and SIRT6 were able to deacetylate **3** (Table 4). The well-known demyristoylases SIRT2, SIRT3, and SIRT6 could demyristoylate **5** (Figure 4). SIRT5 as a deacylase specific for negatively charged acyl residues was able to desuccinylate **7** (Table 4). Using peptides **4** and **8** methylated additionally to the respective acylation showed that neither deacetylation of H4K5AcMe (**4**) nor desuccinylation of H4K5SucMe (**8**) could be achieved by any tested sirtuin (Table 4, Figures S27–S30). Obviously, the additional methyl group at lysine prevents the enzymatic cleavage of the respective amide bond.

**Table 4:** Sirtuin Activity for H4K5Ac (**3**), H4K5AcMe (**4**), H4K5Suc (**7**), and H4K5SucMe (**8**) Peptide Derivatives^a^

SIRT isoform	**3** H4K5Ac^a)^	**4** H4K5AcMe^a)^
SIRT1	++	-
SIRT2	++	-
SIRT3	++	-
SIRT6	+	-
SIRT isoform	**7** H4K5Suc^c)^	**8** H4K5SucMe^a)^
SIRT2	-	-
SIRT3	-	-
SIRT5	+++	-
SIRT6	-	-

a The sirtuin concentration was 50 nM. The peptide concentration was 50 μM. Cleavage was analyzed using UPLC-MS. Activity values reflect substrate conversion based on the intensity of absorbance at 280 nm in the photometric analysis of UPLC-MS data (n = 3). signals: +, <10% conversion; ++, 11–60% conversion; +++, 61–100% conversion after ^a)^24 h and ^b^ 1 h. −, no product detectable by MS.

Inhibition experiments using peptide **4** were performed to analyze if binding to the active site of the sirtuins can occur. At up to 60 μM of **4**, no influence on the activity of SIRT2, SIRT3, and SIRT6 was observed, indicating that the methyl group in addition to the acetyl group on the lysine prevented efficient binding.

Interestingly, complex formation between SIRT5 and an *N*-succinyl-*N-*methyl lysine peptide indicated significant peptide binding and allowed crystallographic analyses of the complex to examine why the catalytic activity is diminished (Figure 3). The crystal structure of the zebrafish SIRT5 ortholog in complex with a KSucMe peptide (2.8 Å resolution; Figure 3A, B; Table S2) reveals the absence of interaction between the lysine side-chain Nε atom and V221 in the SIRT5 active site, which is present in the zSIRT5–KSuc peptide complex (PDB 4UTN; Figure 3A, C, D). A twist of the elongated lysine side chain of the KSucMe peptide brings its succinyl carboxyl group near the NAD^+^ binding pocket and prevents hydrogen bond formation with the R105 and Y102 side chains necessary for catalysis (Figure 3D). The carbonyl oxygen of the tertiary amide bond of KSucMe is turned by about 145° away from the NAD^+^ binding pocket and thus is probably not correctly positioned to attack the 1′ carbon of the nicotinamide ribose in NAD^+^ which initiates the deacetylation process (Figure 3A).

**Figure 3:**
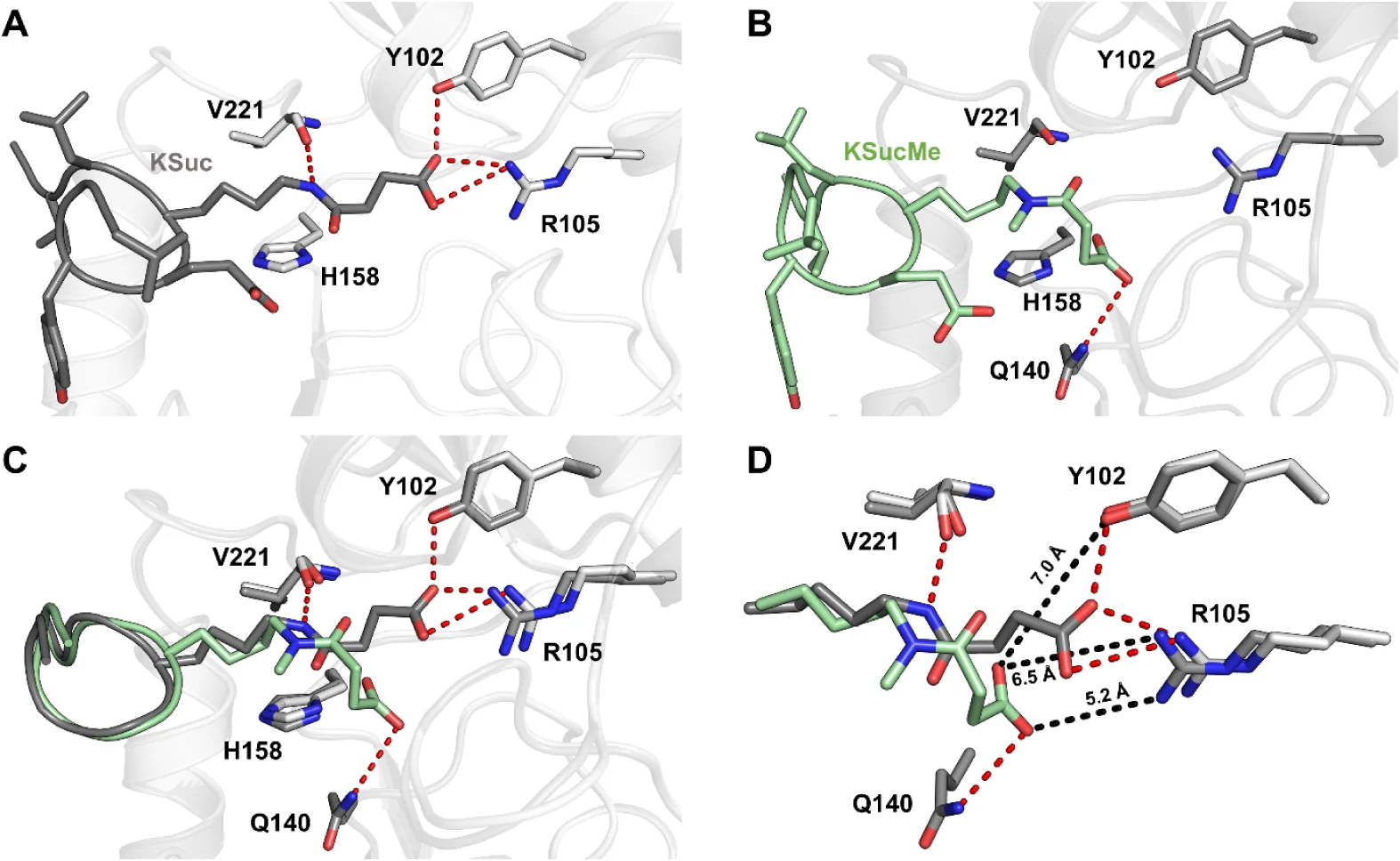
Crystallographic analyses of binding of a KSucMe peptide in comparison to binding of the KSuc peptide to the zSIRT5 active site. (A) Structure of the zSIRT5–KSuc peptide complex (PDB 4UTN). (B) Structure of the zSIRT5–KSucMe peptide complex. (C) Superimposition of the zSIRT5–KSuc peptide (gray) and zSIRT5–KSucMe peptide (light green) complexes illustrates the twist of the elongated lysine side chain of the KSucMe peptide which prevents hydrogen bond formation of the carboxyl group with R105 and Y102 in the zSIRT5 active site found for the zSIRT5–KSuc peptide. (D) Superimposition of the zSIRT5–KSuc peptide (gray) and zSIRT5–KSucMe peptide (light green). Substrate peptides and interacting residues are shown as sticks. The *N*-methyl amide bond places the carboxyl-group oxygens of the KSucMe carbonyl 7.0 Å from Y102 and 6.5 Å and 5.2 Å, respectively, from the R105 guanidinium nitrogens. The interaction of the lysine side chain Nε with the V221 carbonyl oxygen is absent in KSucMe. Red dotted lines indicate hydrogen bonds, black dotted lines show distances.

### Myristoylated Dually Modified Lysine Residues Form Substrates of the Deacylases SIRT2 and SIRT6

Next, we analyzed the ability of the known deacylases of the sirtuin family SIRT2, SIRT3, and SIRT6 with respect to their activity against peptides containing a lysine residue doubly modified with methyl and myristoyl groups. In contrast to SIRT3, SIRT2 and SIRT6 were found to be able to demyristoylate the H4K5MyrMe peptide (**6**) besides their ability to demyristoylate the H4K5Myr peptide (**5**) (Figure 4A, Figures S33–S35). The omission of NAD^+^ in the reaction mixture as a control resulted in the prevention of the reaction, verifying that demyristoylation of **6** occurs via enzymatic catalysis (Figure S41).

**Figure 4:**
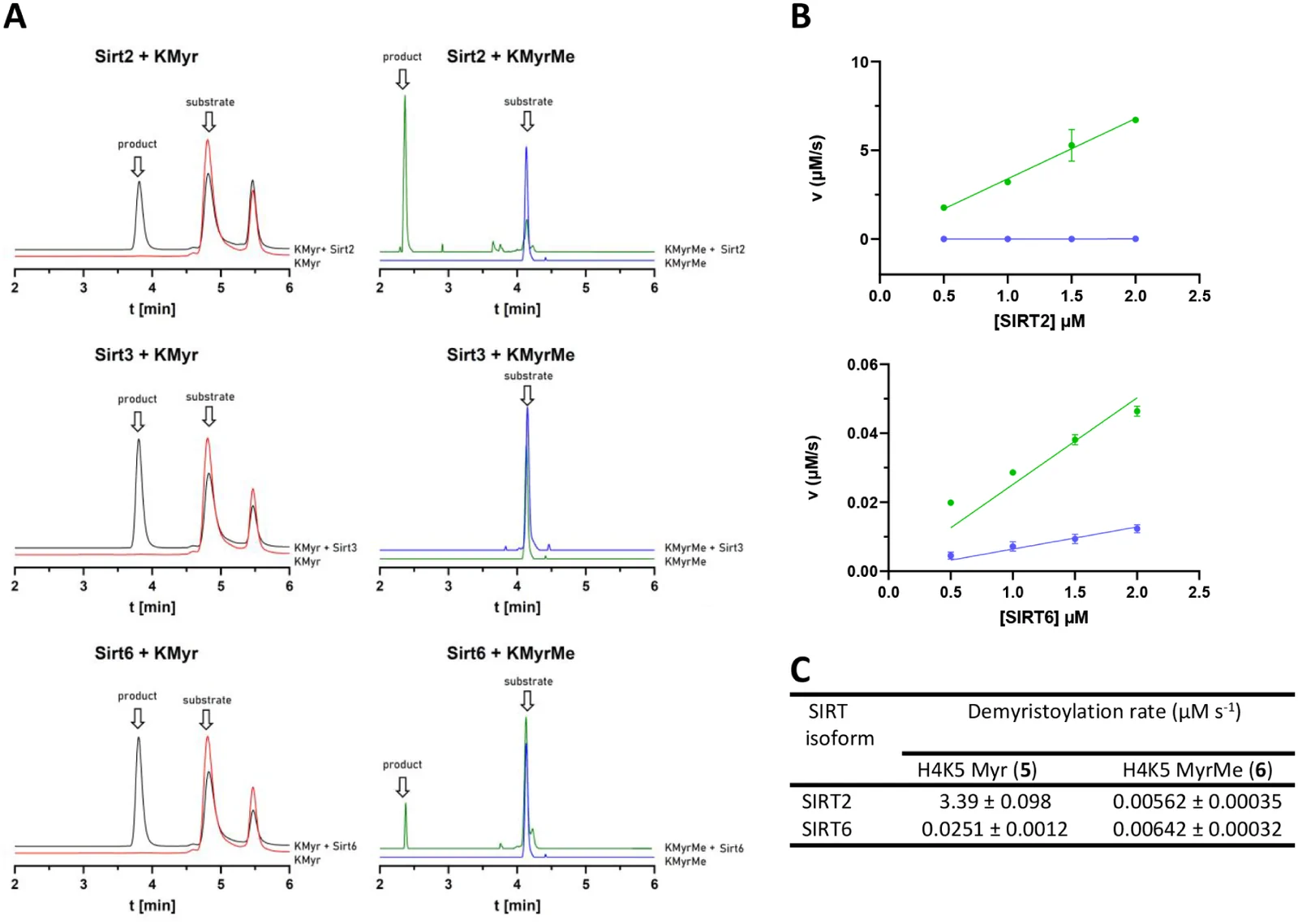
Demyristoylation of H4K5MyrMe (**6**) by SIRT2 and SIRT6. (A) HPLC chromatograms from demyristoylation reactions by SIRT2, SIRT3, and SIRT6. 100 μM of peptide **5** (H4K5Myr) (black, red) or **6** (H4K5MyrMe) (green, blue) were incubated with the respective sirtuin (black, green) or alone (red, blue) for 24 h at 30 °C. Analyses of the samples were performed by RP-HPLC using a C18 column and UV detection at 280 nm. The demyristoylation of peptides caused elution at an earlier retention. (B) Dependence of the velocity of demyristoylation of H4K5Myr (**5**) (green) and H4K5MyrMe (**6**) (blue) on the concentration of the sirtuins SIRT2 (upper panel) and SIRT6 (lower panel). The peptides were used at a concentration of 15 μM. Measurements were performed by HPLC assay. (C) Demyristoylation rates of SIRT2 and SIRT6 for their substrates H4K5Myr (**5**) and H4K5MyrMe (**6**) at a substrate concentration of 15 μM and an enzyme concentration of 1 μM. Rates are expressed as mean ± standard error. All measurements were carried out in triplicate.

In order to quantify the substrate properties of **5** and **6** for SIRT2 and SIRT6, progress curves of the demyristoylation reactions at different enzyme concentrations were recorded (Figure 4B) and demyristoylation rates were obtained (Figure 4C). As expected, SIRT2 efficiently demyristoylated **5** with a rate of 3.39 μM s^–1^. This rate was approximately 135-fold higher than that for SIRT6 with 0.0251 μM s^–1^. The additional methylation of the myristoyl-lysine caused about a 600-fold reduction of the demyristoylation rate of SIRT2 and about a 4-fold reduction of the demyristoylation rate of SIRT6. Both enzymes catalyze the demyristoylation of **6** with similar rates of about 0.006 μM s^–1^ (Figure 4C). Taken together, the enzymatic activity was reduced but still existent in the case of myristoyl-methyl-lysine in the substrate peptide for SIRT2 and SIRT6.

The structural basis for the reduced yet detectable demyristoylation of KMyrMe by SIRT2 was examined using peptide **12**, whose H3K9 sequence represents a well-established SIRT2 substrate.^62^ Two structures were solved: SIRT2–**12** (PDB 30KD, 1.45 Å resolution; Figure 5A) and SIRT2–**12**–NAD^+^ (PDB 30KE, 2.80 Å resolution; Figure 5B) (Table S2). In the SIRT2–**12** complex, the myristoyl group occupies the large hydrophobic acyl-lysine channel formed at the interface of the Zn^2+^-binding and Rossmann-fold domains. The KMyrMe dual modification introduces steric bulk that forces the *N*-methyl group to rotate toward the Rossmann-fold domain. Consequently, KMyrMe adopts a *cis*-amide conformation in which the *N*-methyl and carbonyl groups orient toward H187, a catalytically essential residue (Figure 5A, S42). This rotation moves the amide away from V233, whose backbone carbonyl normally forms a key hydrogen bond with the amide nitrogen in the unmethylated substrate, as presented by comparison of the structures of the complexes SIRT2–**12** and SIRT2–H3K9Myr (PDB 4Y6L) (Figure S42). Loss of this stabilizing interaction weakens substrate anchoring in the channel, consistent with the rather weak electron density observed for the KMyrMe moiety of peptide **12**.

**Figure 5:**
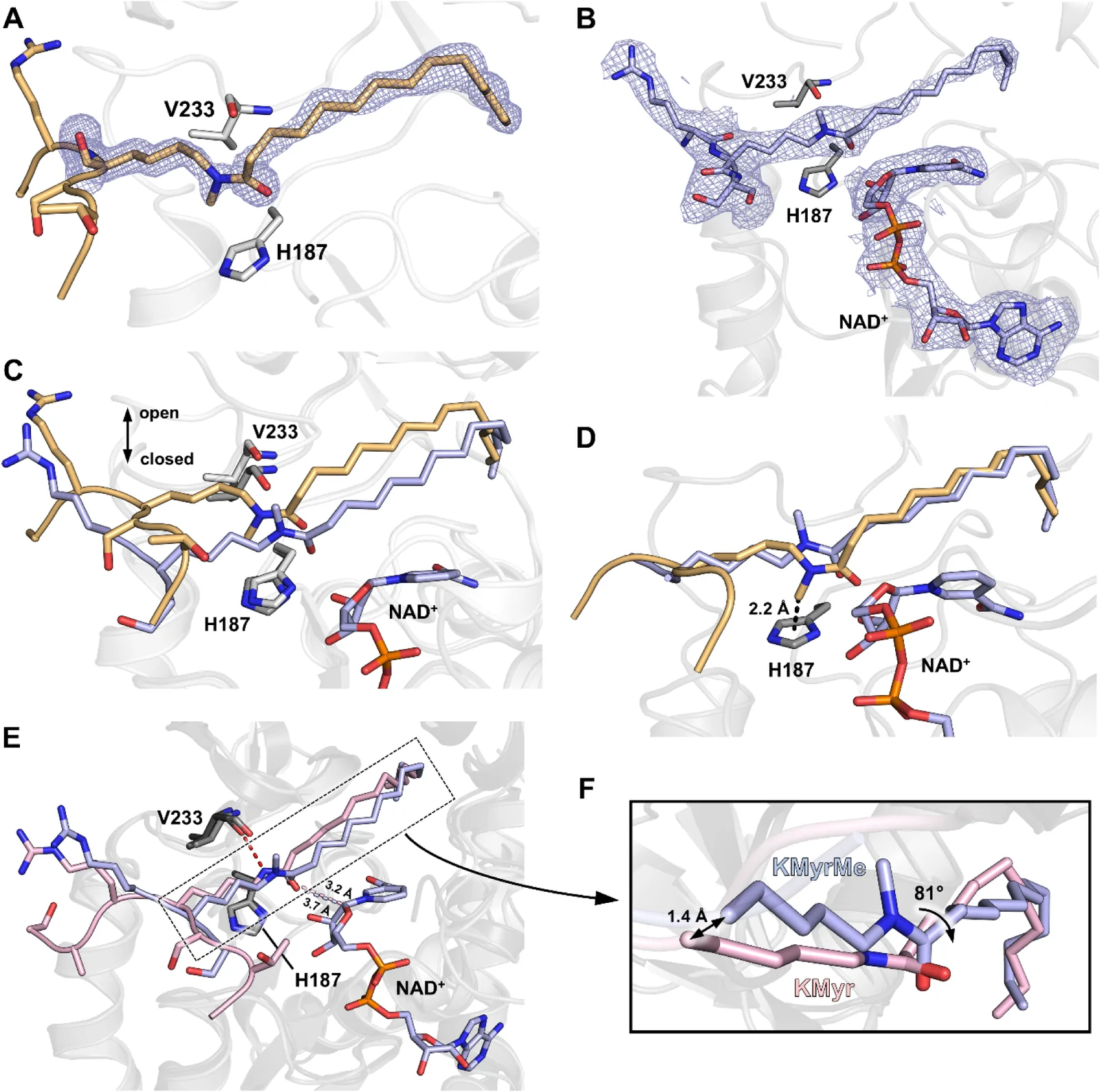
Structural analysis of the SIRT2–**12** (PDB 30KD) and SIRT2–**12**–NAD^+^ (PDB 30KE) complexes. (A) Structure of the SIRT2–**12** complex: To avoid steric clashes with the Val233 backbone, the *N*-methyl amide bond of KMyrMe adopts a *cis* conformation. The 2F_o_–F_c_ map of the KMyrMe moiety is shown as a blue mesh contoured at 0.9σ. (B) Structure of the SIRT2–**12**–NAD^+^ complex: Upon NAD^+^ binding, the *N*-methyl amide bond adopts a *trans* conformation. Clear electron density for the NAM moiety of NAD^+^ reflects the markedly reduced catalytic turnover of KMyrMe. The 2F_o_–F_c_ map is shown as a blue mesh contoured at 0.9σ. (C) Superimposition of SIRT2–**12** (yellow–orange, open state) and SIRT2–**12**–NAD^+^ (light blue, closed state) reveals NAD^+^-induced closure of the Zn^2+^-binding domain, driving *cis*–*trans* isomerization of the *N*-methyl amide bond in peptide **12** through steric hindrance between the *N*-methyl group and H187. (D) Alignment of peptide **12** in both structures shows that retaining the *cis* conformation upon domain closure would generate steric clashes with H187 and the ribose moiety of NAD^+^. (E) Superposition of SIRT2–**12**–NAD^+^ with SIRT2–H3K9Myr (light pink, PDB 4Y6L) illustrates that rotation of the *N*-methyl amide bond places the KMyrMe carbonyl 3.7 Å from the C1′ of NAD^+^ ribose, putting the C=O in a suboptimal position and distance for the initial nucleophilic attack. (F) Close-up view shows the 1.4 Å shift of the K9 Cα atom in KMyrMe versus KMyr upon entry into the acyl lysine channel and the 81° rotation of the *N*-methyl amide bond. These features, together with steric hindrance, likely account for the ∼600-fold reduction in catalytic turnover of KMyrMe relative to KMyr.

The SIRT2–**12**–NAD^+^ structure was obtained by soaking NAD^+^ into preformed SIRT2–**12** crystals for 6 h (Figure 5B). Clear electron density for the nicotinamide (NAM) moiety of NAD^+^ aligns well with the biochemical data, which show markedly reduced demyristoylation of KMyrMe compared to KMyr (see Figure 4). This reduction likely arises because nucleophilic attack of the amide C=O on the C1′ of NAD^+^ ribose, required for NAM cleavage, is impaired. Moreover, NAD^+^ binding induces an unusual ribose conformation, in which the 2′–OH and 3′–OH groups kink to avoid steric interference with the atypical KMyrMe carbonyl orientation.

Notably, the myristoyl amide bond of KMyrMe switches from *cis* to *trans* in the NAD^+^-bound structure. Superposition of SIRT2–**12** and SIRT2–**12**–NAD^+^ reveals the structural basis for this isomerization. In the absence of NAD^+^, SIRT2–**12** adopts an open, unproductive conformation. NAD^+^ binding triggers closure of the Zn^2+^-binding domain (Figure 5C), which would position the *N*-methyl of the *cis* amide bond only 2.2 Å from His187 (Figure 5D). This steric clash potentially forces isomerization to the *trans*-species. Because NAD^+^ was soaked into preformed crystals, the reduced resolution of the NAD^+^-bound structure likely reflects slight disruption of the crystal lattice caused by SIRT2 domain movement.

Comparison of SIRT2–**12**–NAD^+^ with SIRT2–H3K9Myr (PDB 4Y6L)^35^ further clarifies the reduced catalytic turnover. To accommodate peptide **12** in the acyl-lysine channel, the K9 Cα atom shifts by 1.4 Å relative to KMyr, allowing an 81° rotation of KMyrMe compared to KMyr and preventing a clash with V233 (Figure 5E,F). However, this repositioning yields a suboptimal angle and distance between the amide C=O and the C1′ of the NAD^+^ ribose (3.7 Å versus 3.2 Å for KMyr), reducing the efficiency of nucleophilic attack. This structural arrangement aligns with the observed diminished yet measurable enzymatic activity.

(A) Structure of the SIRT2–**12** complex: To avoid steric clashes with the Val233 backbone, the *N*-methyl amide bond of KMyrMe adopts a *cis* conformation. The 2F_o_–F_c_ map of the KMyrMe moiety is shown as a blue mesh contoured at 0.9σ. (B) Structure of the SIRT2–**12**–NAD^+^ complex: Upon NAD^+^ binding, the *N*-methyl amide bond adopts a *trans* conformation. Clear electron density for the NAM moiety of NAD^+^ reflects the markedly reduced catalytic turnover of KMyrMe. The 2F_o_–F_c_ map is shown as a blue mesh contoured at 0.9σ. (C) Superimposition of SIRT2–**12** (yellow–orange, open state) and SIRT2–**12**–NAD^+^ (light blue, closed state) reveals NAD^+^-induced closure of the Zn^2+^-binding domain, driving *cis*–*trans* isomerization of the *N*-methyl amide bond in peptide **12** through steric hindrance between the *N*-methyl group and H187. (D) Alignment of peptide **12** in both structures shows that retaining the *cis* conformation upon domain closure would generate steric clashes with H187 and the ribose moiety of NAD^+^. (E) Superposition of SIRT2–**12**–NAD^+^ with SIRT2–H3K9Myr (light pink, PDB 4Y6L) illustrates that rotation of the *N*-methyl amide bond places the KMyrMe carbonyl 3.7 Å from the C1′ of NAD^+^ ribose, putting the C=O in a suboptimal position and distance for the initial nucleophilic attack. (F) Close-up view shows the 1.4 Å shift of the K9 Cα atom in KMyrMe versus KMyr upon entry into the acyl lysine channel and the 81° rotation of the *N*-methyl amide bond. These features, together with steric hindrance, likely account for the ∼600-fold reduction in catalytic turnover of KMyrMe relative to KMyr.

## Discussion

The *in vivo* existence of a dually modified lysine residue carrying both a methyl and an acetyl group at the ε-amino group was recently found on histone H4 in a range of species and mammalian tissues.^6^ The modification was shown to be generated enzymatically by the successive action of a KMT and a HAT (Figure 6, 1, 3).^6^ KMTs are described to be specific for the amino group of lysine and do not readily methylate other nitrogen nucleophiles, making an initial HAT-mediated lysine acetylation (Figure 6, 2) followed by KMT-mediated methylation unlikely (Figure 6, 4).^63^

**Figure 6:**
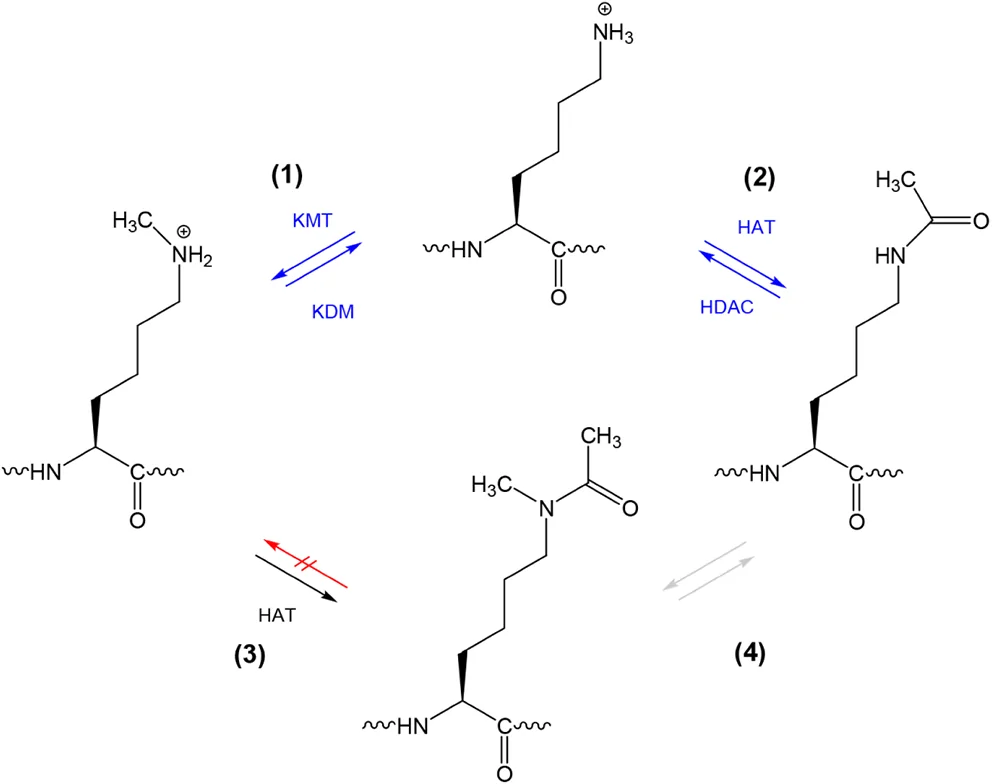
Schematic representation of the possible reactions for modification of a single lysine by acetylation and/or methylation. KMT and KDM control reversible methylation (1), HAT and HDAC control reversible acetylation (2) in the case of a single modification (blue). Acetylation of methyl-lysine residues to produce the dually modified KAcMe can be performed by HAT, as exemplified for p300 (3).^6^ Reversion of this route by HDAC-mediated KAcMe deacetylation was not found in this work (3). Methylation of the poor amide nitrogen nucleophile of KAc and demethylation of KAcMe (4) has not been envisioned yet.

The removal of the KAcMe modification could be envisaged by either deacetylation followed by demethylation or the other way around (Figure 6).

In the study presented here, we have shown that in the analyzed context, acetyl groups of methylated acetyl lysines are virtually not enzymatically removable by lysine deacetylases, neither by Zn^2+^-dependent HDACs nor by sirtuins (Tables 1 and 4).

For deacylation analyses, we used model peptides either with a sequence derived from histone H4K5 or with an artificial sequence described to interact very efficiently with HDACs, so that the corresponding unmethylated peptide formed a substrate for the lysine deacetylase investigated. As a more reactive analogue of the acetyl residue, the trifluoroacetyl residue was used for HDACs of class IIb.^56^ Possibly, HDAC substrate properties of peptides may differ from those of folded proteins forming *in vivo* substrates. However, since histone tails, where the KAcMe modification was found, are intrinsically disordered and devoid of three-dimensional structure, peptides form useful models to study the enzymology of histone PTMs.^64–66^ The *in vitro* assay used was designed to be suitable to find even very slow deacetylation of KAcMe residues by the use of high enzyme concentrations and long reaction times. The conditions used would allow detection of enzymatic reactions for substrates with very low catalytic efficiencies, with *k*_cat_/K_M_ values as low as 2 M^–1^ s^–1^.

Several lysine deacylases are known to have distinct specificity for the acyl group they remove. Whereas HDAC8 and 11 and sirtuins SIRT1–3 and SIRT6 efficiently deacylate long acyl chains,^17,35,59,62,67^ SIRT5 is specific for cleavage of negatively charged acyl groups like succinyl residues from lysine.^68^ Therefore, we analyzed for these deacylases their ability to remove the respective acyl group in the additional presence of a methyl residue at the ε-amino group of lysine. The natural occurrence of these modifications is not known; however, spontaneous acylation of methyl lysine residues by the respective CoA derivatives is chemically feasible. Furthermore, the acetyltransferase p300 is known to transfer different acyl residues besides acetyl residues like the succinyl, propionyl and butyryl residues.^69,70^ Despite their specific ability to deacylate the unmethylated counterparts, the respective Zn^2+^-dependent HDACs were not able to deacylate *N-*myristoylated *N*-methylated peptide derivatives and SIRT5 was not able to remove a succinyl group from the respective dually labeled peptide derivatives in our experiments.

For most HDACs, KAcMe deacetylation was precluded because the KAcMe-containing peptides were unable to bind to the active sites of the respective enzymes. Only HDAC6, 10, and 11 could interact with a KAcMe-containing peptide as indicated by the efficient inhibition of enzymatic catalysis (Table 2). Docking studies for HDAC6 ^15^ and 11 showed that the overall binding of dually modified peptides to the HDAC active sites to be similar to that of unmethylated peptides. This similarity of the interactions during binding may explain the relatively high affinity of the dually modified peptides toward the HDAC active sites. However, the additional lysine methylation of the substrates causes a loss of possible hydrogen-bond formation and a slightly disturbed binding mode of the lysine side chain which may account for the lack of catalysis (Figure 2). Similarly, the crystal structure analysis of SIRT5 reveals a disturbed binding of the KAcMe peptide derivative (Figure 3). Whereas the Nε of the lysine side chain of the KSuc peptide interacts with Val221 in the SIRT5 active site, this interaction is missing for the KSucMe peptide derivative. A turn of the peptide leads to a misalignment of the carbonyl group of the scissile bond in relation to NAD^+^ precluding the deacetylation reaction.

The inability of HDACs to remove acetyl groups from KAcMe suggests KAcMe to be a quasi-permanent mark *in vivo* taking into account that demethylation of KAcMe has not been envisioned yet. If the additional modification does not interfere with readers of the single modifications, permanence would prevent deactivation. In the case of histones, permanent histone acetylation would mean a permanent permissive “switched on” state of chromatin allowing access by chromatin-remodeling factors. Alternatively, KAcMe may constitute a completely new signal recognized by specific readers with yet unknown biological implications. The dual modification may also mask one of the single modifications supplying an additional level of readout control. In either case, the irreversibility of the acetylation event implies the importance of long-term control and the existence of an alternative possibility of displacement.

There are other PTMs of amino acid side chains in proteins known to be irreversible. Often they are caused by spontaneous chemical processes connected with protein aging;^71,72^ however, also enzymatic modifications like the deimination of arginine into citrulline represent nonreversible PTMs.^73^ Irreversible PTMs are considered to enable rather longer-lasting signal transduction and regulation of biochemical processes.^73^

The *in vivo* results of Lu-Culligan *et al.* showing that the addition of the pan-HDAC inhibitor trichostatin A (TSA) increased the level of H4K5AcMe peptides from cellular histone proteins imply that HDAC-mediated reversibility of H4K5AcMe formation may not necessarily arise from Zn^2+^-dependent HDACs involved in the removal of the acetyl group of methylated acetyl-lysine residues.^6^ A possible alternative explanation may be offered by the TSA-induced inhibition of HDACs leading to acetylation and thereby the activation of the histone acetyltransferase p300 shown to acetylate methyl-lysine residues.^74,75^

Surprisingly, a fatty acyl group in the additional presence of a methyl group on the lysine could be removed by the two sirtuins SIRT2 and SIRT6, even though with very low activity (Figure 4). The structural analysis of SIRT2-**12** complexes implies that interactions of the hydrophobic tail of the fatty acyl chain anchor the substrate peptide in the active site in a position with sufficiently correct placement of the scissile bond to allow catalysis (Figure 5). The reduction in enzymatic activity may result from repositioning of the lysine Cα atom to prevent a clash with V233 in the SIRT2 active site, which leads to a suboptimal angle and distance between the amide C=O and the C1′ of the NAD^+^ ribose (Figure 5E,F) for nucleophilic attack. Generally, the reversibility of fatty acylation on methylated lysine residues may be important in a biological context because of the large impact of fatty acylations on protein localization.^76^

## Conclusion

In conclusion, we show that HDACs are virtually not able to remove the acetyl group from KAcMe residues in the context of H4K5. Also, other acyl residues could not be removed in the additional presence of a methyl residue at the ε-amino group of lysine by the HDACs known to be specific for the respective acyl group. The origin of the prohibition of catalysis could be structurally clarified by a disrupted binding mode of the substrate peptide in the enzyme’s active site. Surprisingly, as an exception, SIRT2 and SIRT6 were found to be able to demyristoylate the H4K5MyrMe peptide even though with very low activity. Our results suggest that dual modification may feature an additional level of regulation by lysine side-chain modifications especially with regard to long-term control.

## Supplementary Material









## Data Availability

Diffraction data and the refined models have been deposited with the Protein Data Bank for the zSIRT5-KSucMe-CPS1 peptide complex (zSIRT5–NmetNsuccCPS1) under accession code 30KU, for the SIRT2–**12** complex under accession code 30KD, and for the SIRT2–**12**–NAD^+^ complex under accession code 30KE.

## Notes

Data for SIRT2-12 complex (CIF)
Data for SIRT2-12-NAD+ complex (CIF)
Data for zSIRT5-KSucMe-CPS1 peptide complex (CIF)
UPLC-MS data, analyses of enzymatic deacylation experiments, dose–response curves for inhibitor measurements, and crystallographic data (PDF)
