# Supplementary material for: Structural and Enzymatic Basis for Impaired Deacylation of N‑Acyl‑N‑Methyl Lysine Residues
Source: Biochemistry. 2026 Sep 8;65(19):3092–105. doi: 10.1021/acs.biochem.6c00508 (PMC13648224; doi:10.1021/acs.biochem.6c00508)
Supplement: Supplementary file 1 [file bi6c00508_si_004.pdf]

Supporting Information

Structural and enzymatic basis for impaired deacylation of N-acyl-N-methyl lysine residues

Sebastian Hilscher[1], Paul Horst Böttcher[2], Florian Friedrich[3], Martin Pannek[6], Fady Baselious[1], Marat Meleshin[2], Oliver Einsle[4], Cyril Barinka[5], Clemens Steegborn[6], Wolfgang Sippl[1], Manfred Jung[3], Mike Schutkowski[2*] and Cordelia Schiene-Fischer[2*]

[1] Department of Medicinal Chemistry, Institute of Pharmacy, Martin-Luther-University Halle-Wittenberg, Kurt-Mothes-Str. 3, 06120 Halle/Saale (Germany)

[2] Department of Enzymology, Charles Tanford Protein Center, Institute of Biochemistry and Biotechnology, Martin-Luther-University Halle-Wittenberg, Kurt-Mothes-Str. 3a, 06120 Halle/Saale (Germany)

[3] Institute of Pharmaceutical Sciences, University of Freiburg, Albertstr. 25 79104, Freiburg (Germany)

[4] Institute of Biochemistry, University of Freiburg, Albertstr. 21, 79104, Freiburg (Germany)

[5] Institute of Biotechnology of the Czech Academy of Sciences, BIOCEV, Prumyslova 595, 252 50 Vestec (Czech Republic)

[6] Department of Biochemistry, University of Bayreuth, Universitaetsstr. 30, 95447, Bayreuth (Germany)

* Corresponding authors: mike.schutkowski@biochemtech.uni-halle.de, cordelia.schiene-fischer@biochemtech.uni-halle.de

A. UPLC-MS data of the synthesized compounds
B. UPLC-MS analysis of enzymatic deacylation experiments of the dually modified lysine peptides
C. Dose-response curves
D. HPLC-based deacylation analysis and controls
E. Crystallographic data

A. UPLC-MS data of the synthesized compounds

Table S1: Overview about the molecular mass of the synthesized compounds.

Compound	Calculated mass [g/mol]	m/z of $[M+2H]^{2+}$	Found mass [g/mol]	calculated mass - found mass [g/mol]
**1**	1215.63	608.87	1215.74	-0.11
**2**	1229.64	615.97	1229.94	-0.3
**3**	1257.64	630.00	1258	-0.36
**4**	1271.65	636.87	1271.74	-0.09
**5**	1425.82	714.18	1426.36	-0.54
**6**	1439.84	721.18	1440.36	-0.52
**7**	1315.64	658.87	1315.74	-0.1
**8**	1329.66	665.87	1329.74	-0.08
**9**	1338.47	670.08	1338.16	0.31
**10**	1299.51	650.67	1299.34	0.17
**11**	956.60	479.76	957.52	-0.92
**12**	985.62	493.76	985.52	0.1
**13**	1013.65	507.77	1013.54	0.11
**14**	1041.68	521.77	1041.54	0.14
**15**	999.63	500.77	999.54	0.09
**16**	1027.66	514.77	1027.54	0.12
**17**	1041.68	521.77	1041.54	0.14
**18**	1039.70	520.77	1039.54	0.16
**19**	1055.70	528.87	1055.74	-0.04
**20**	1069.71	535.78	1069.56	0.15
**21**	1089.68	545.68	1089.36	0.32
**22**	1069.71	535.78	1069.56	0.15

UPLC-MS analysis of the used peptides

UPLC-MS analysis was performed using either a Waters Acquity UPLC-MS system with a Waters Acquity-UPLC-MS-BEH C18, 1.7 µm (2.1 mm × 50 mm, 30 Å) column. As a mobile phase, 0.1% formic acid in H2O (solvent A) and 0.1% formic acid in acetonitrile (ACN) (solvent B) solutions were used. A typical gradient from 95:5 to 5:95 (v/v) H_2O:ACN in 6 min followed by reequilibration (6-10 min) was used for most of the runs. Data analysis was performed using Waters MassLynx software.

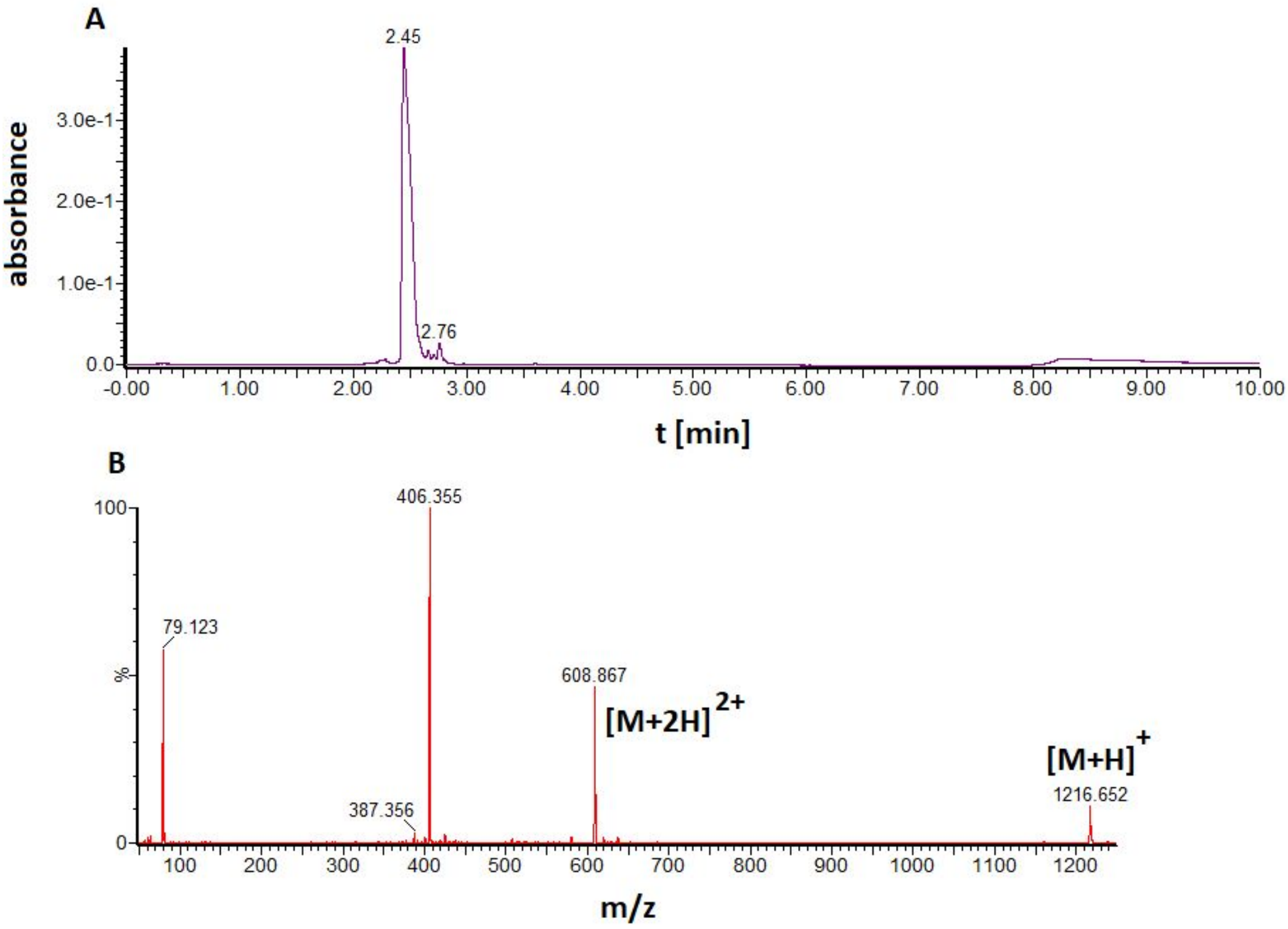

Figure S1: UPLC-MS Spectra of compound **1**. **A**. The photometric analysis was done between 200 to 400 nm (here shown at 280 nm). **B**. Mass spectra of the peak at 2.45 min.

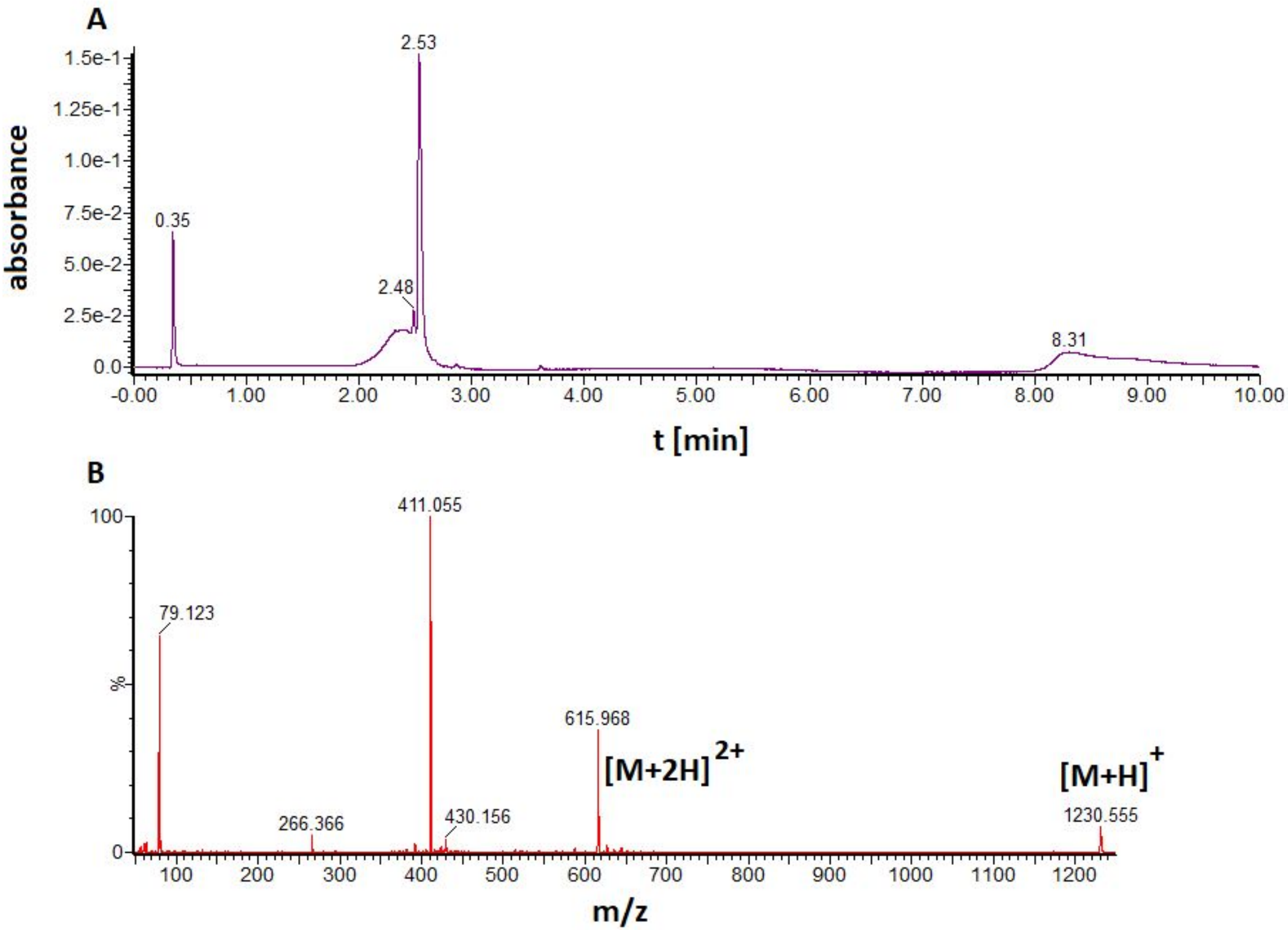

Figure S2: UPLC-MS Spectra of compound **2**. **A**. The photometric analysis was done between 200 to 400 nm (here shown at 280 nm). **B**. Mass spectra of the peak at 2.53 min.

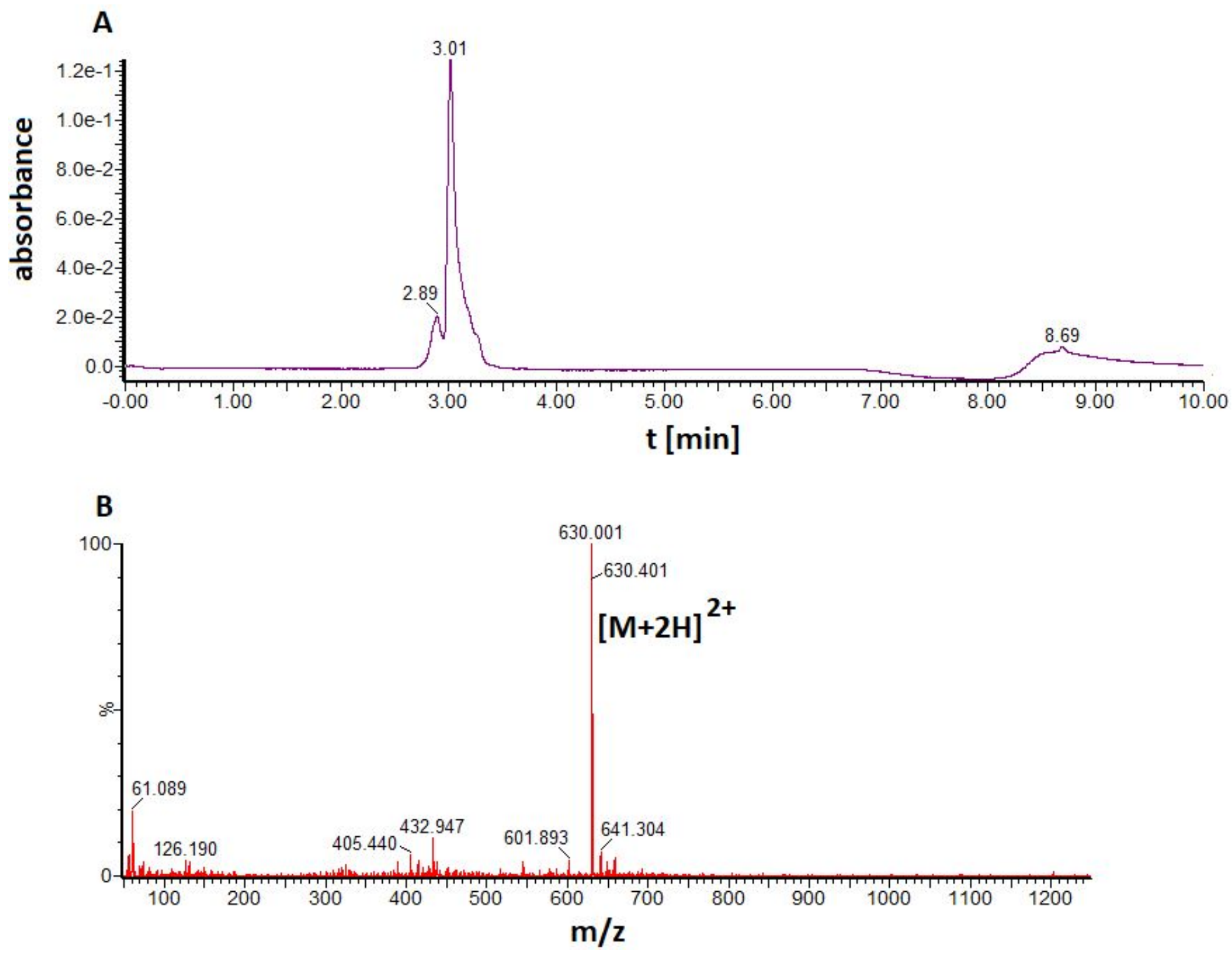

Figure S3: UPLC-MS Spectra of compound **3** **A**. The photometric analysis was done between 200 to 400 nm (here shown at 280 nm). **B**. Mass spectra of the peak at 3.01 min.

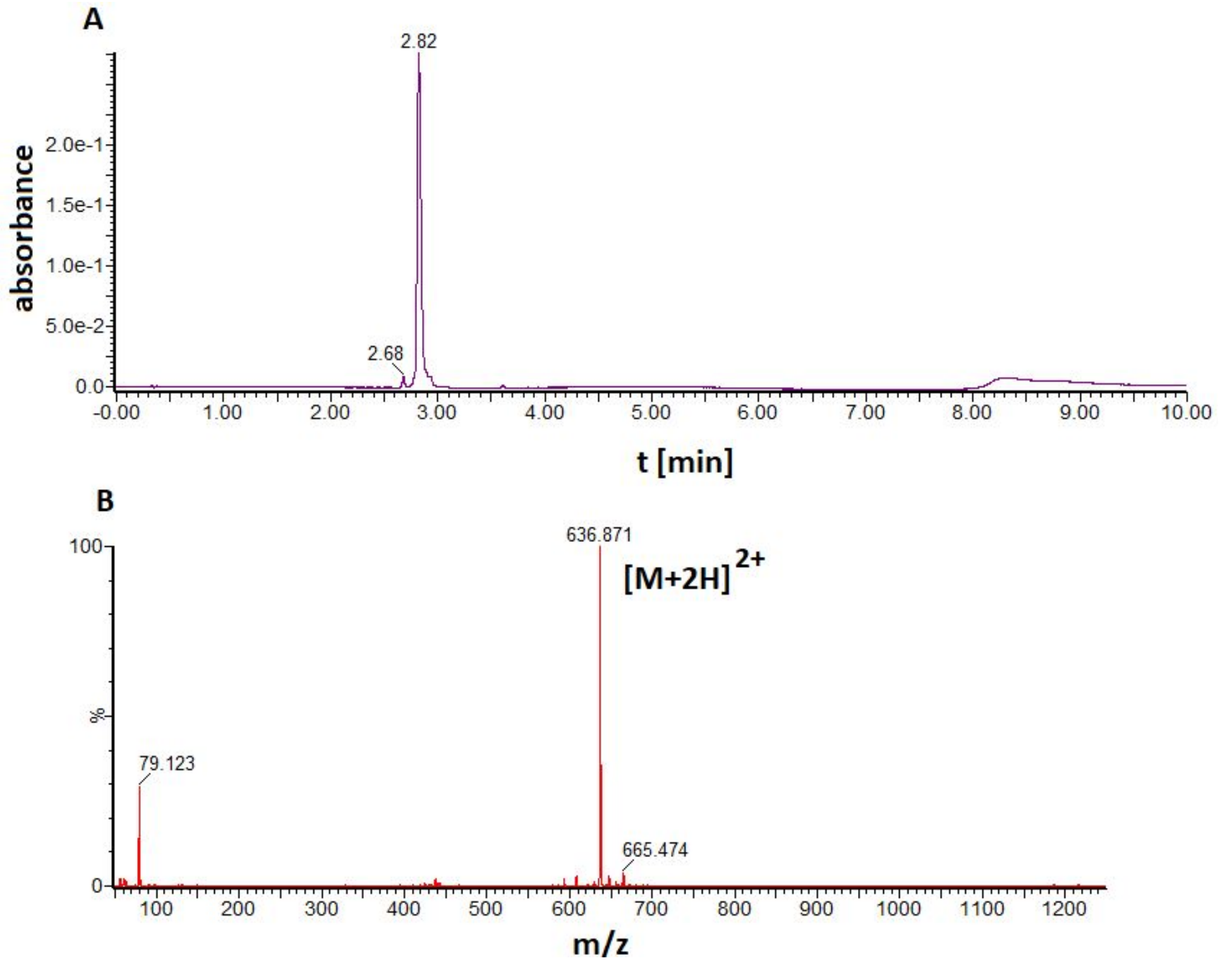

Figure S4: UPLC-MS Spectra of compound **4**. **A**. The photometric analysis was done between 200 to 400 nm (here shown at 280 nm). **B**. Mass spectra of the peak at 2.82 min.

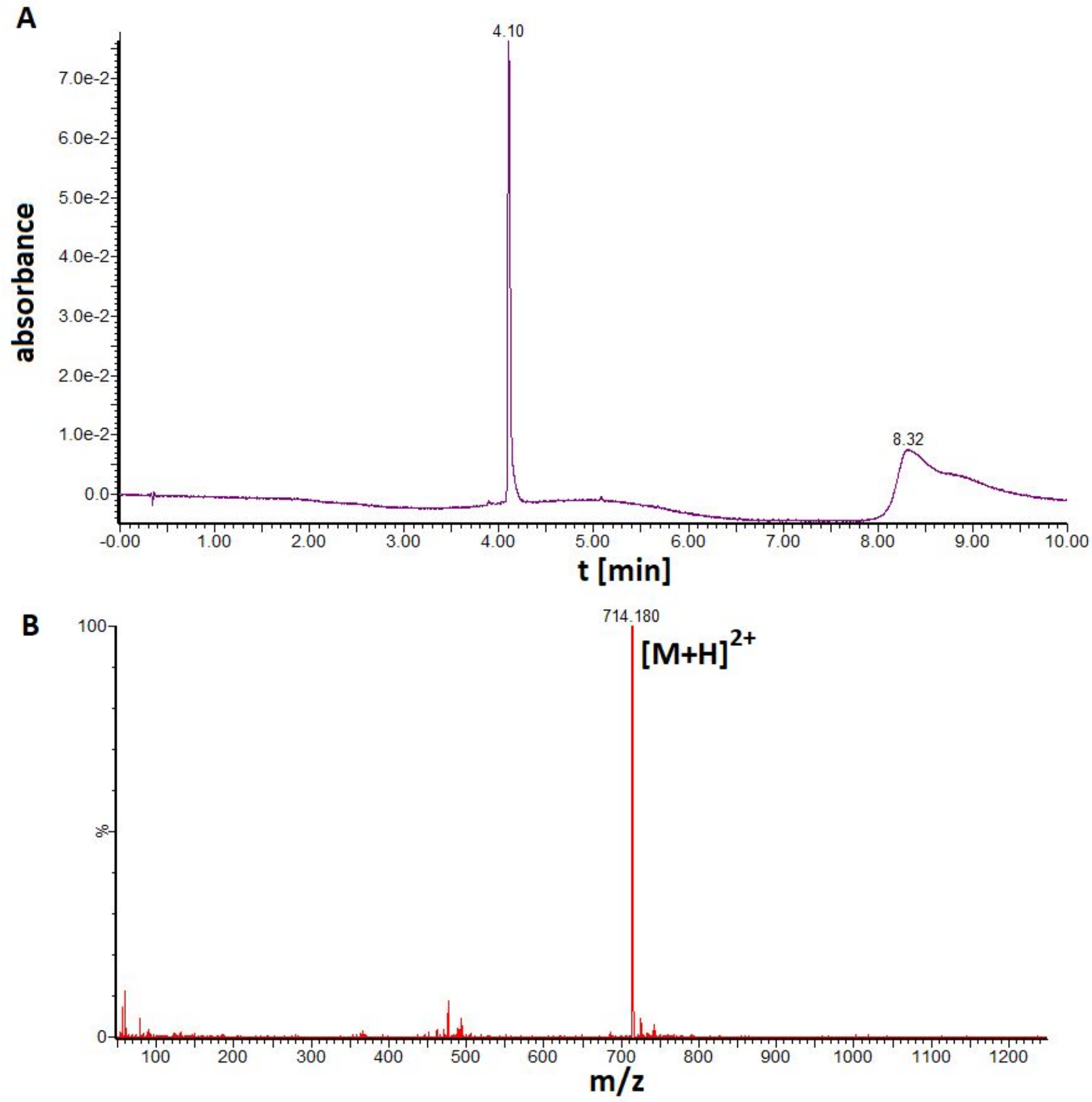

Figure S5: UPLC-MS Spectra of compound **5**. **A**. The photometric analysis was done between 200 to 400 nm (here shown at 280 nm). **B**. Mass spectra of the peak at 4.10 min.

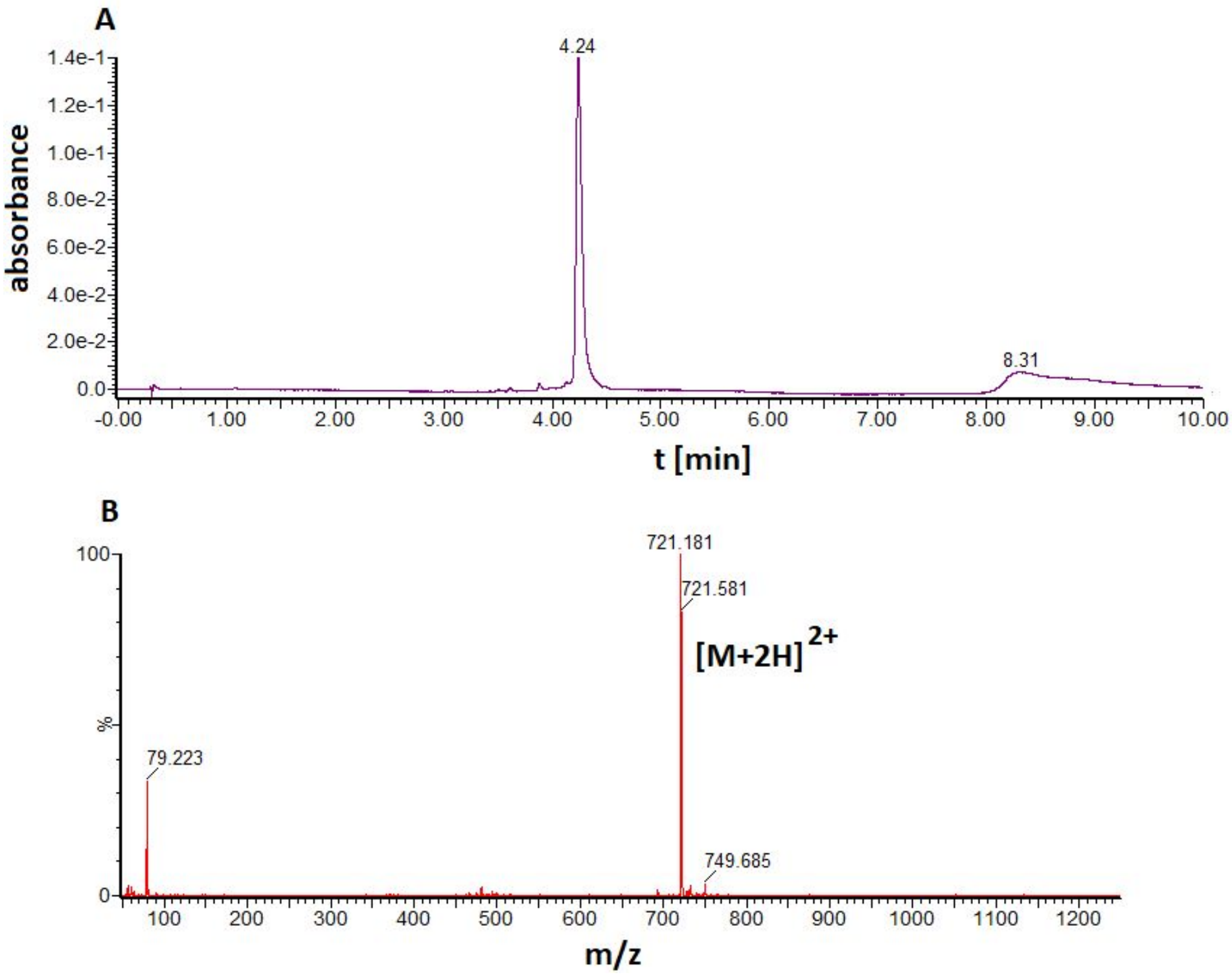

Figure S6: UPLC-MS Spectra of compound **6**. **A**. The photometric analysis was done between 200 to 400 nm (here shown at 280 nm). **B**. Mass spectra of the peak at 4.24 min.

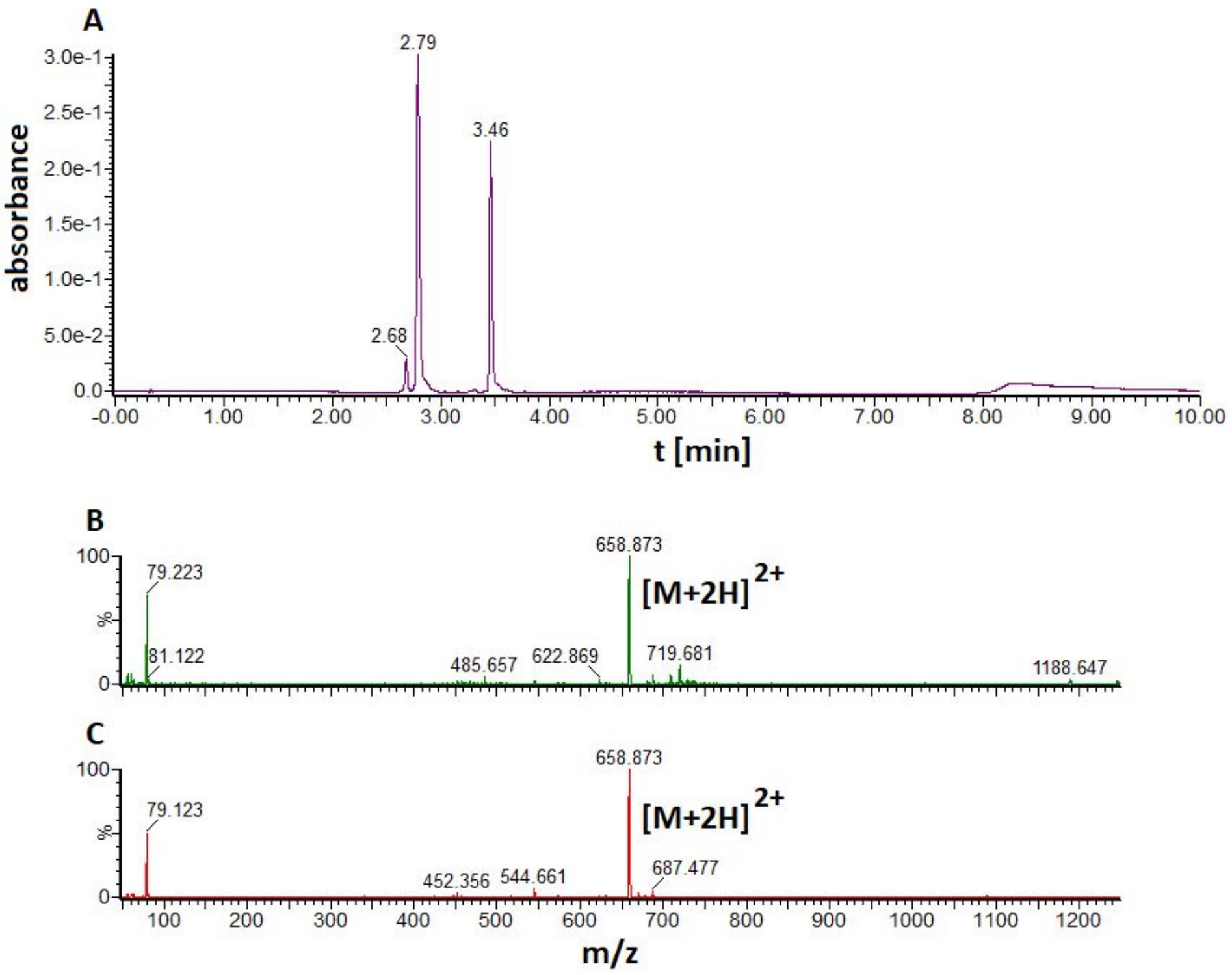

Figure S7: UPLC-MS Spectra of compound **7**. **A**. The photometric analysis was done between 200 to 400 nm (here shown at 280 nm). **B**. Mass spectra of the peak at 2.79 min. **C**. Mass spectra of the peak at 3.46 min

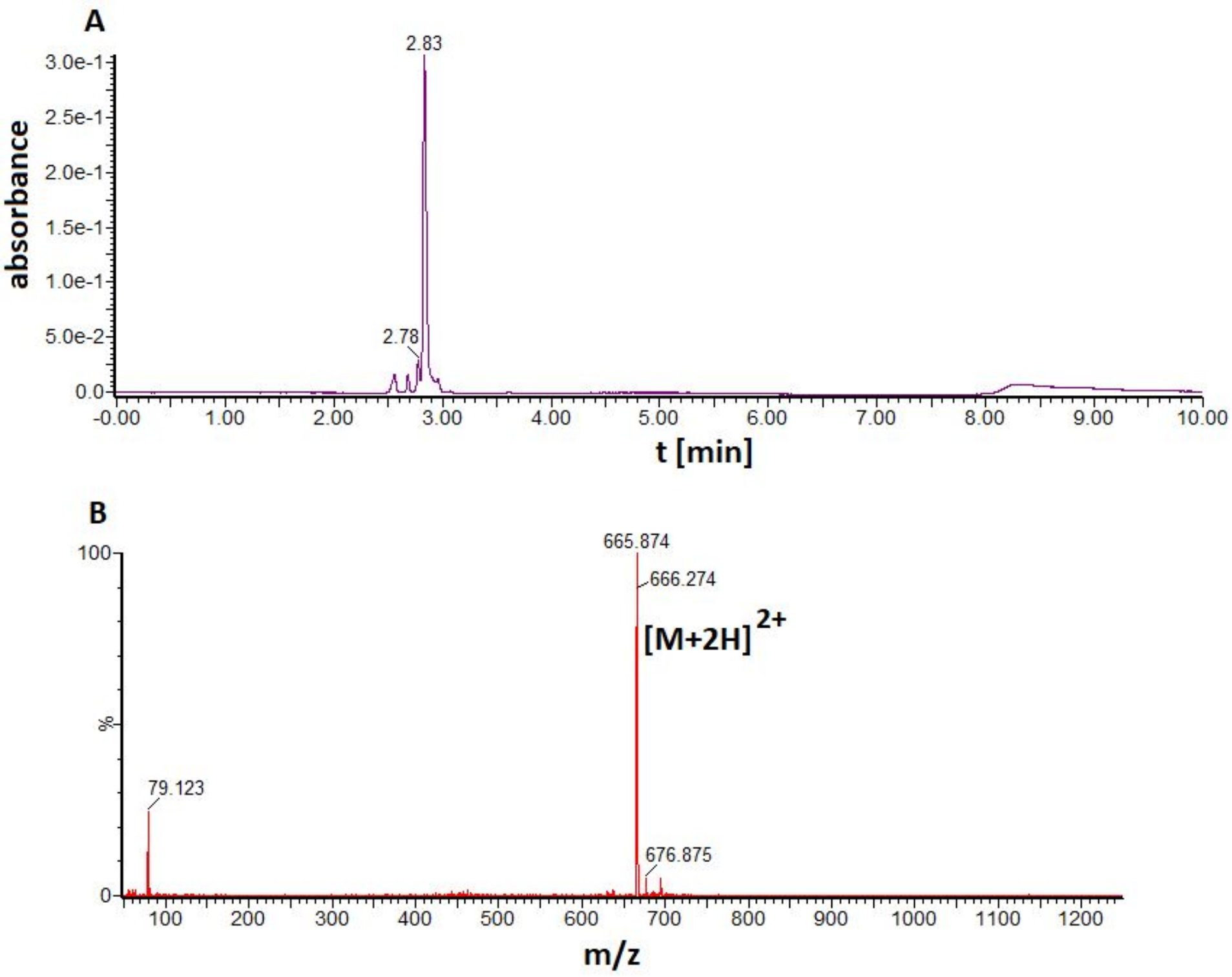

Figure S8: UPLC-MS Spectra of compound **8**. **A**. The photometric analysis was done between 200 to 400 nm (here shown at 280 nm). **B**. Mass spectra of the peak at 2.83 min.

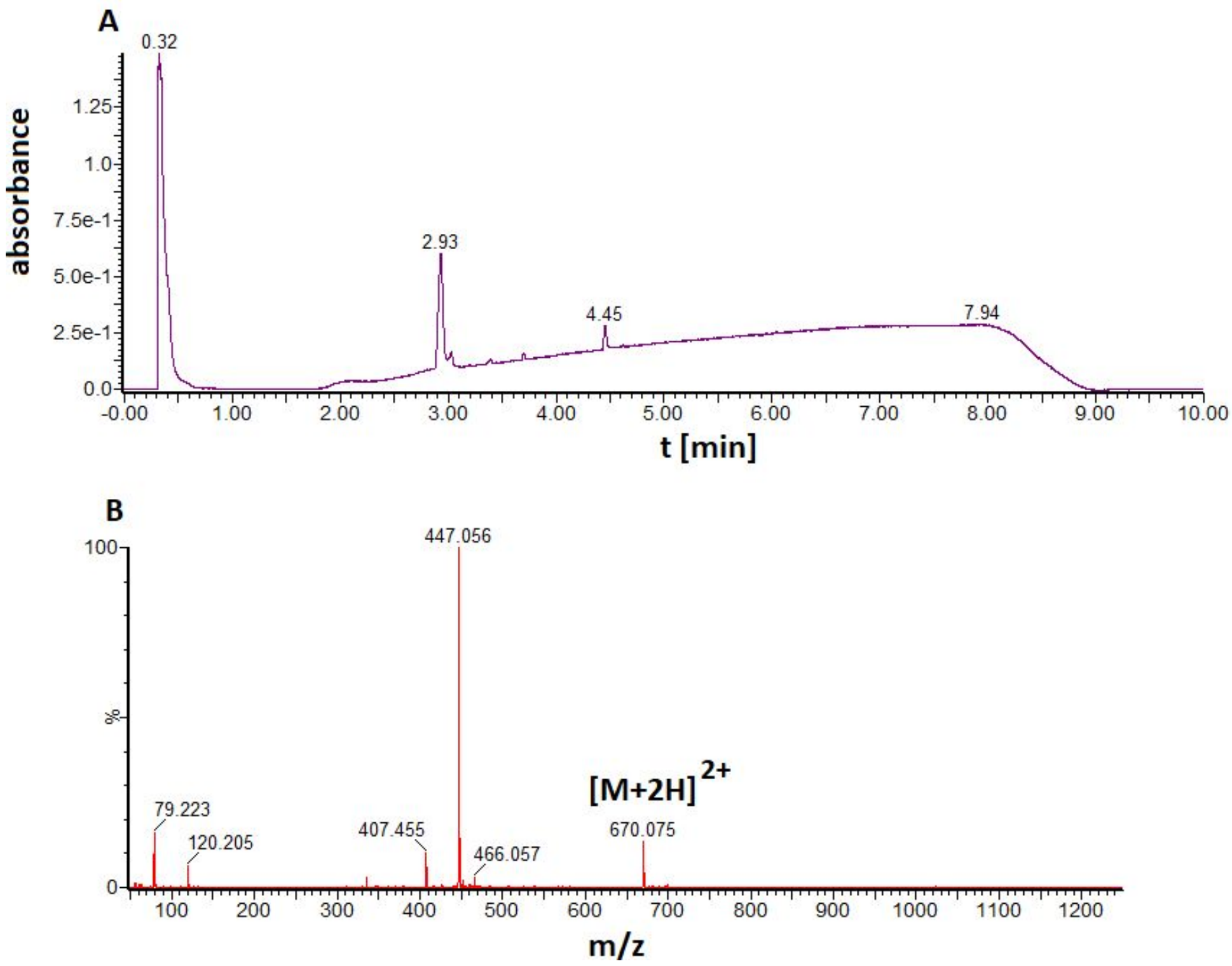

Figure S9: UPLC-MS Spectra of compound **9**. **A**. The photometric analysis was done between 200 to 400 nm (here shown at 220 nm). **B**. Mass spectra of the peak at 2.93 min.

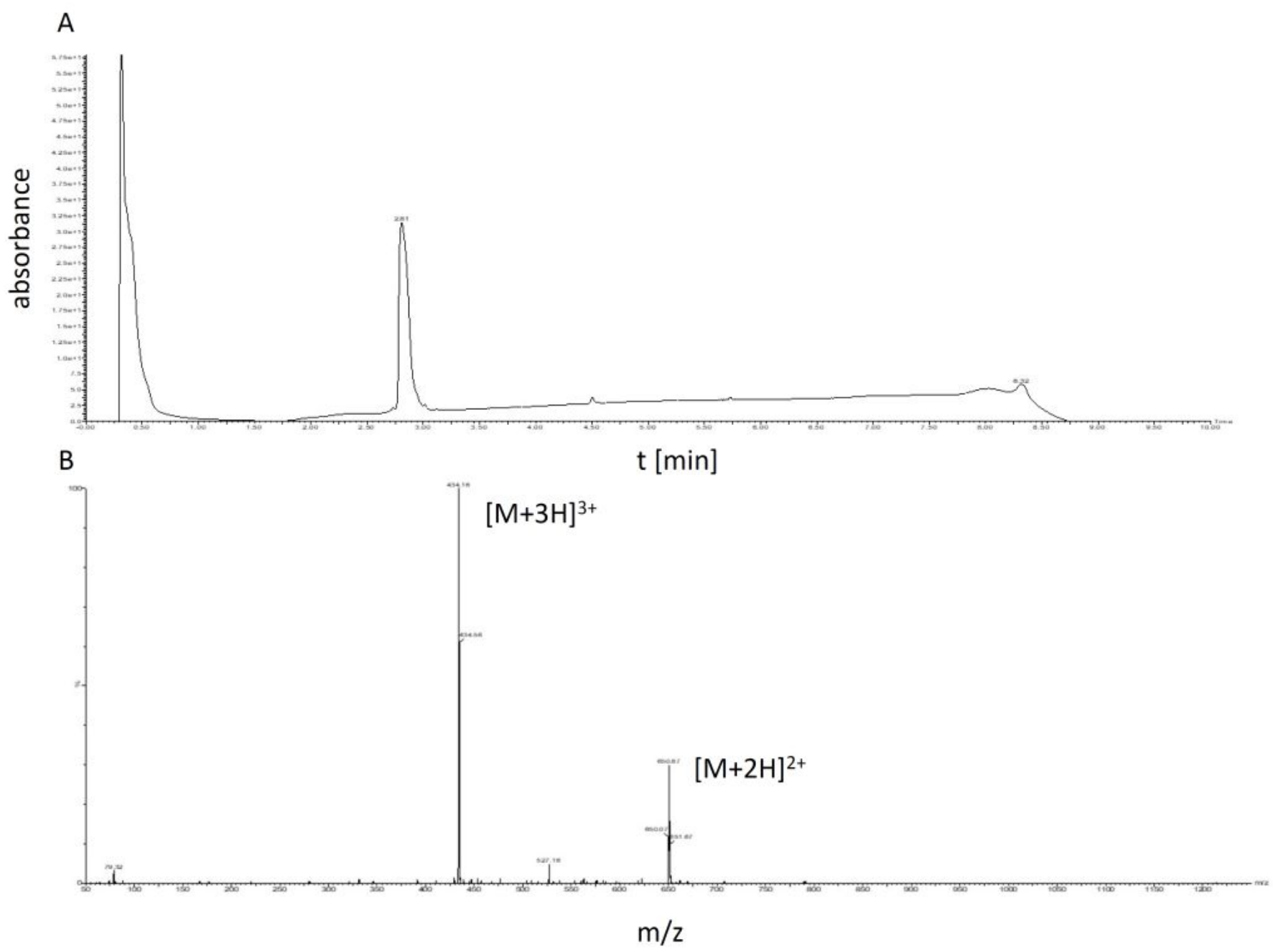

Figure S10: UPLC-MS Spectra of compound **10**. **A**. The photometric analysis was done between 200 to 400 nm (here shown at 220 nm). **B**. Mass spectra of the peak at 2.93 min.

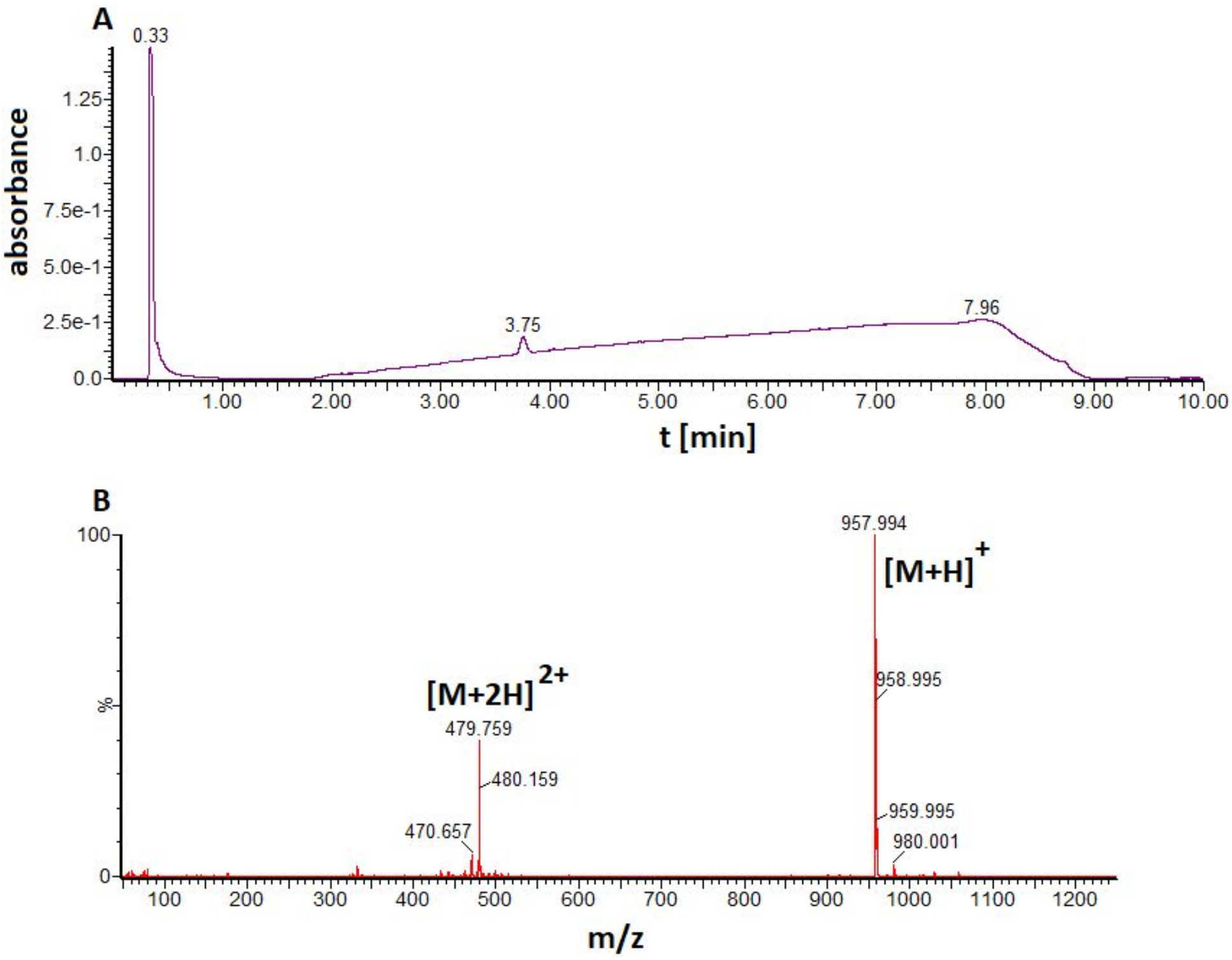

Figure S11: UPLC-MS Spectra of compound **11**. **A**. The photometric analysis was done between 200 to 400 nm (here shown at 220 nm). **B**. Mass spectra of the peak at 3.75 min.

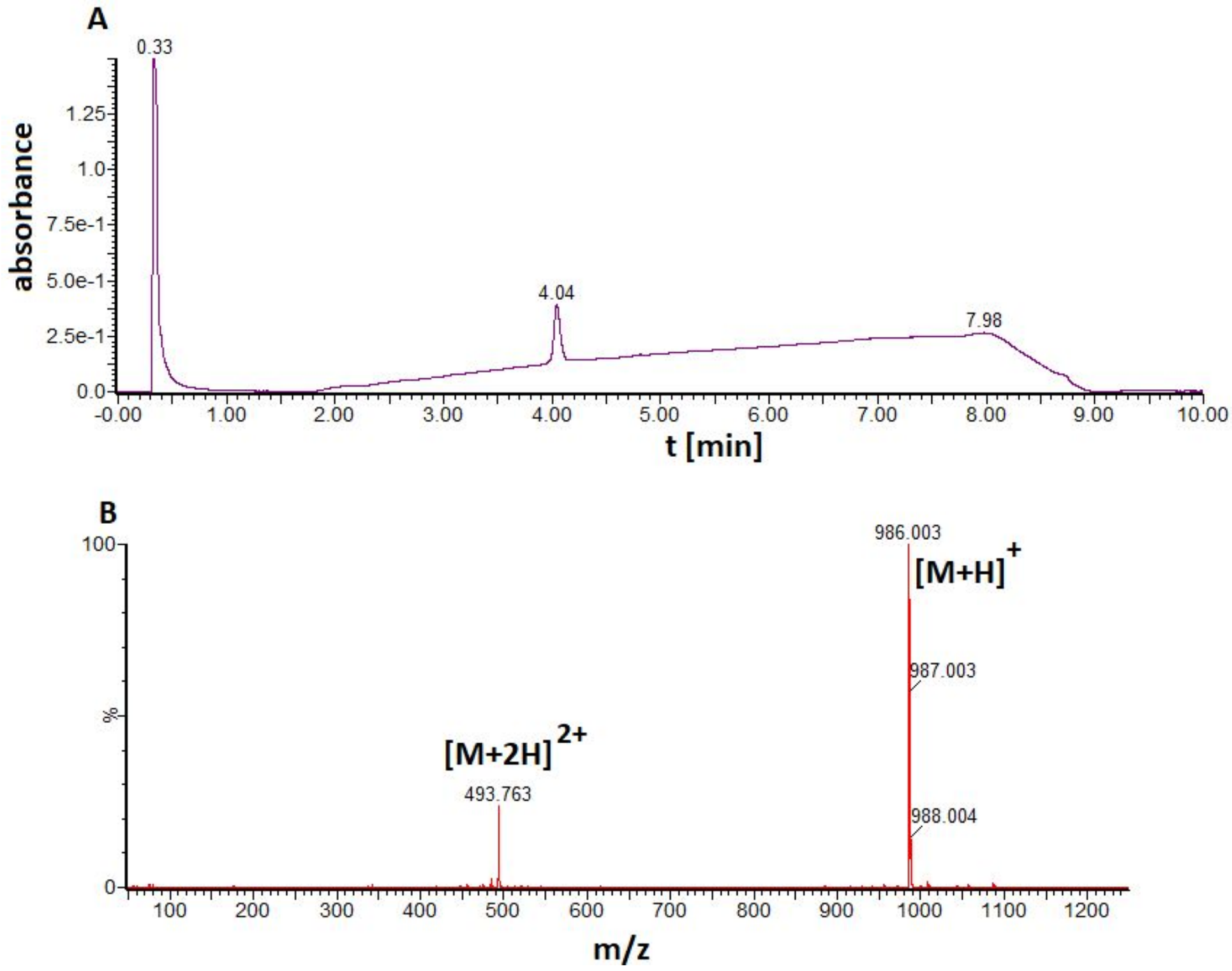

Figure S2: UPLC-MS Spectra of compound **12**. **A**. The photometric analysis was done between 200 to 400 nm (here shown at 220 nm). **B**. Mass spectra of the peak at 4.04 min.

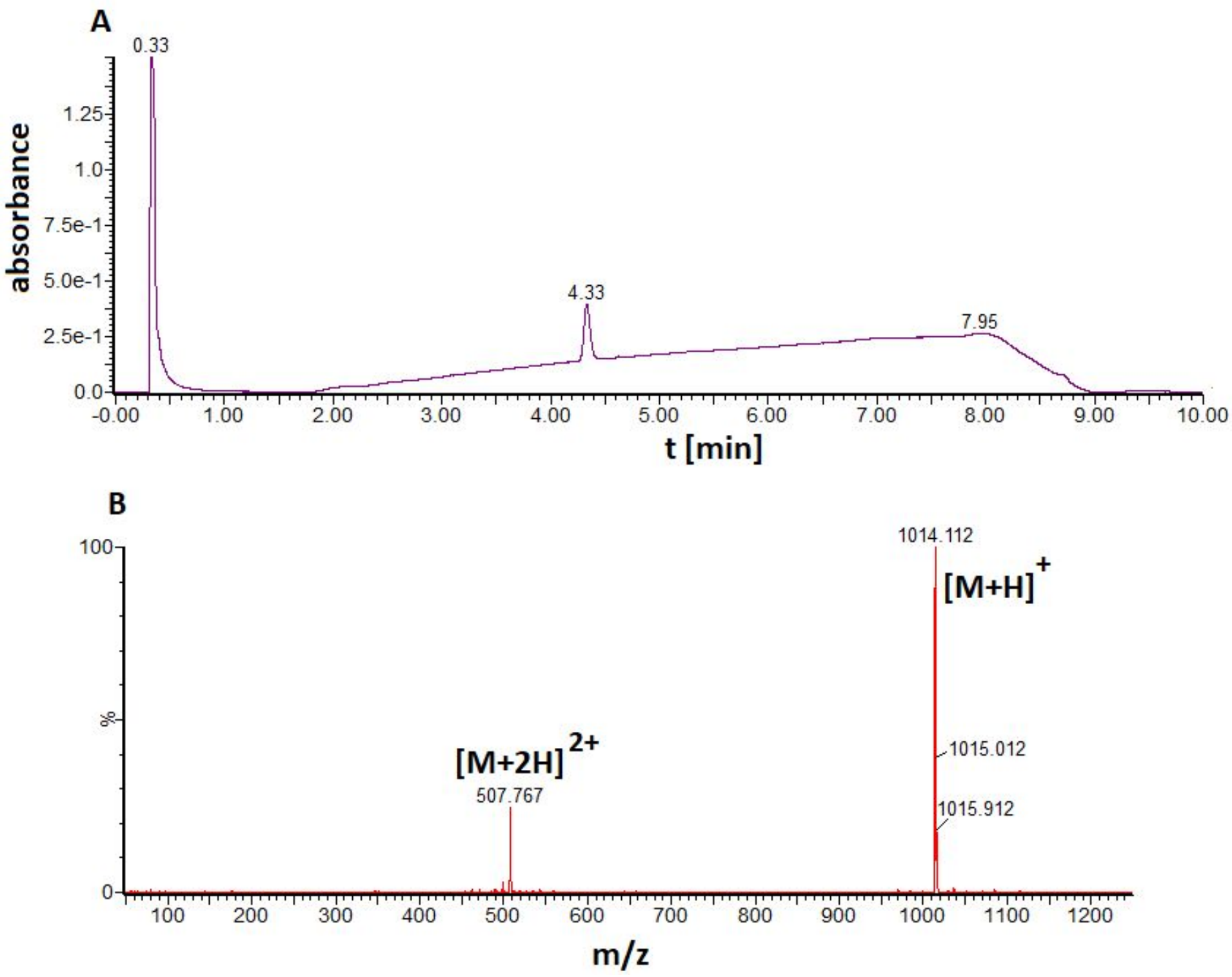

Figure S13: UPLC-MS Spectra of compound **13**. **A**. The photometric analysis was done between 200 to 400 nm (here shown at 220 nm). **B**. Mass spectra of the peak at 4.33 min.

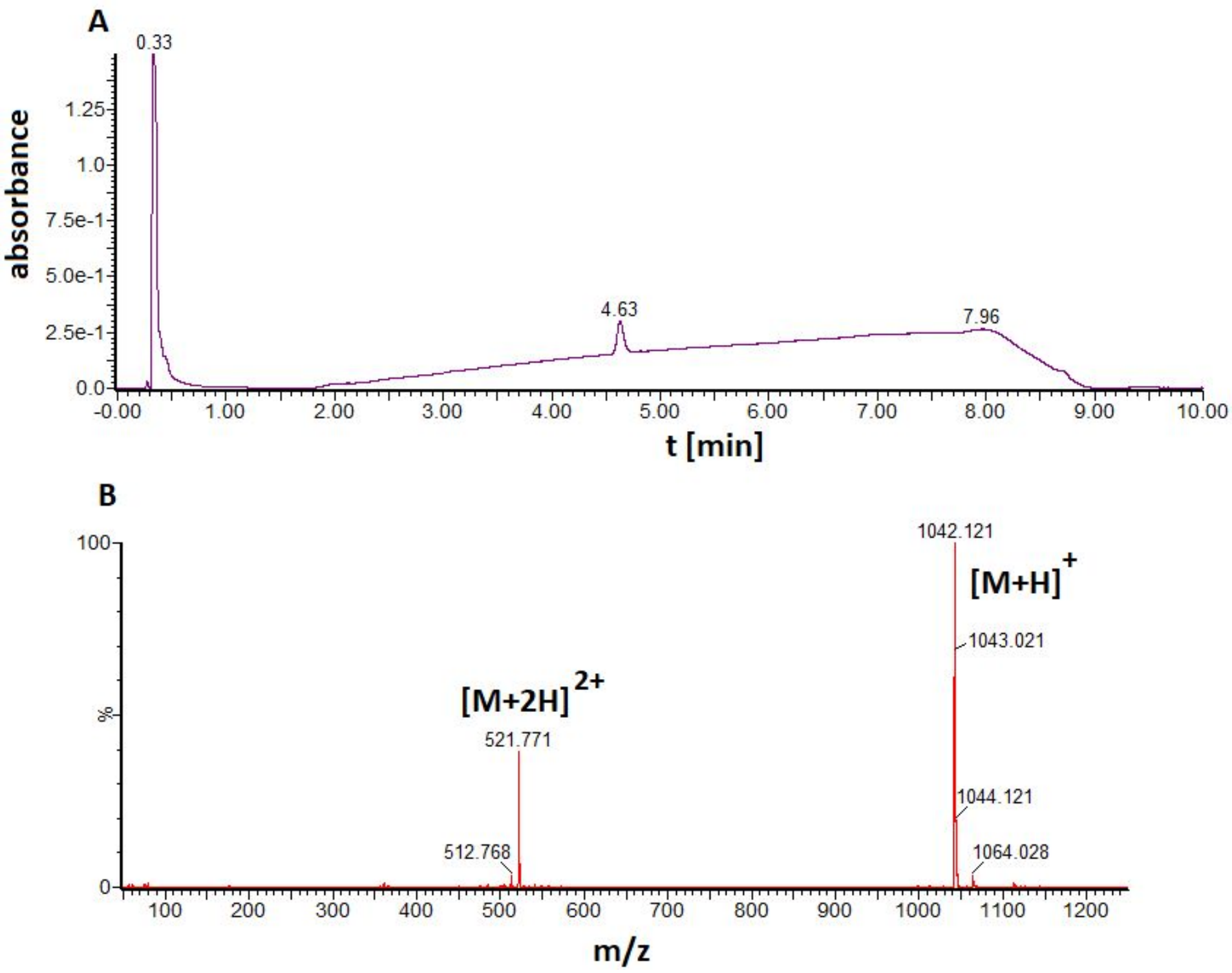

Figure S14: UPLC-MS Spectra of compound **14**. **A**. The photometric analysis was done between 200 to 400 nm (here shown at 220 nm). **B**. Mass spectra of the peak at 4.63 min.

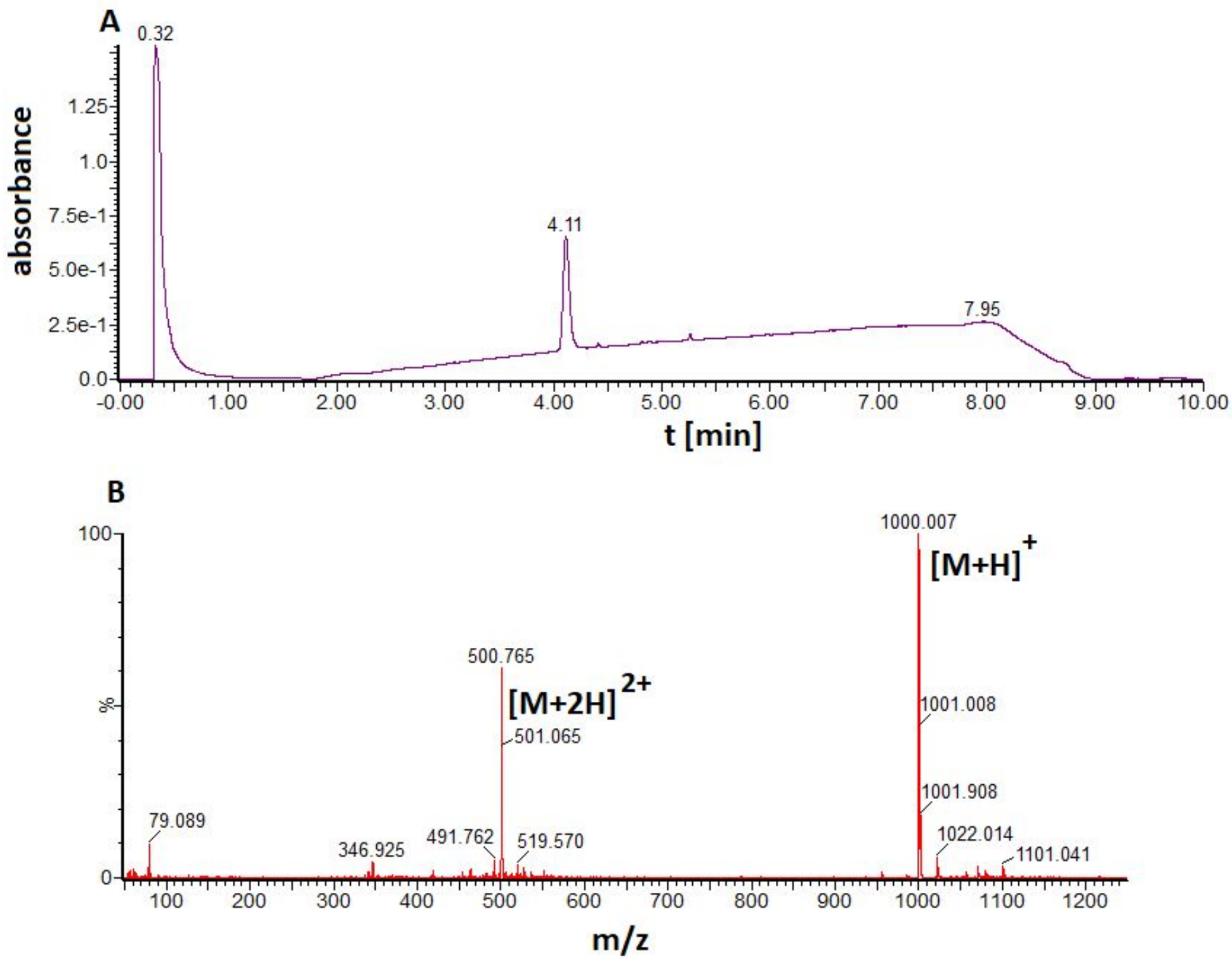

Figure S15: UPLC-MS Spectra of compound **15**. **A**. The photometric analysis was done between 200 to 400 nm (here shown at 220 nm). **B**. Mass spectra of the peak at 4.11 min.

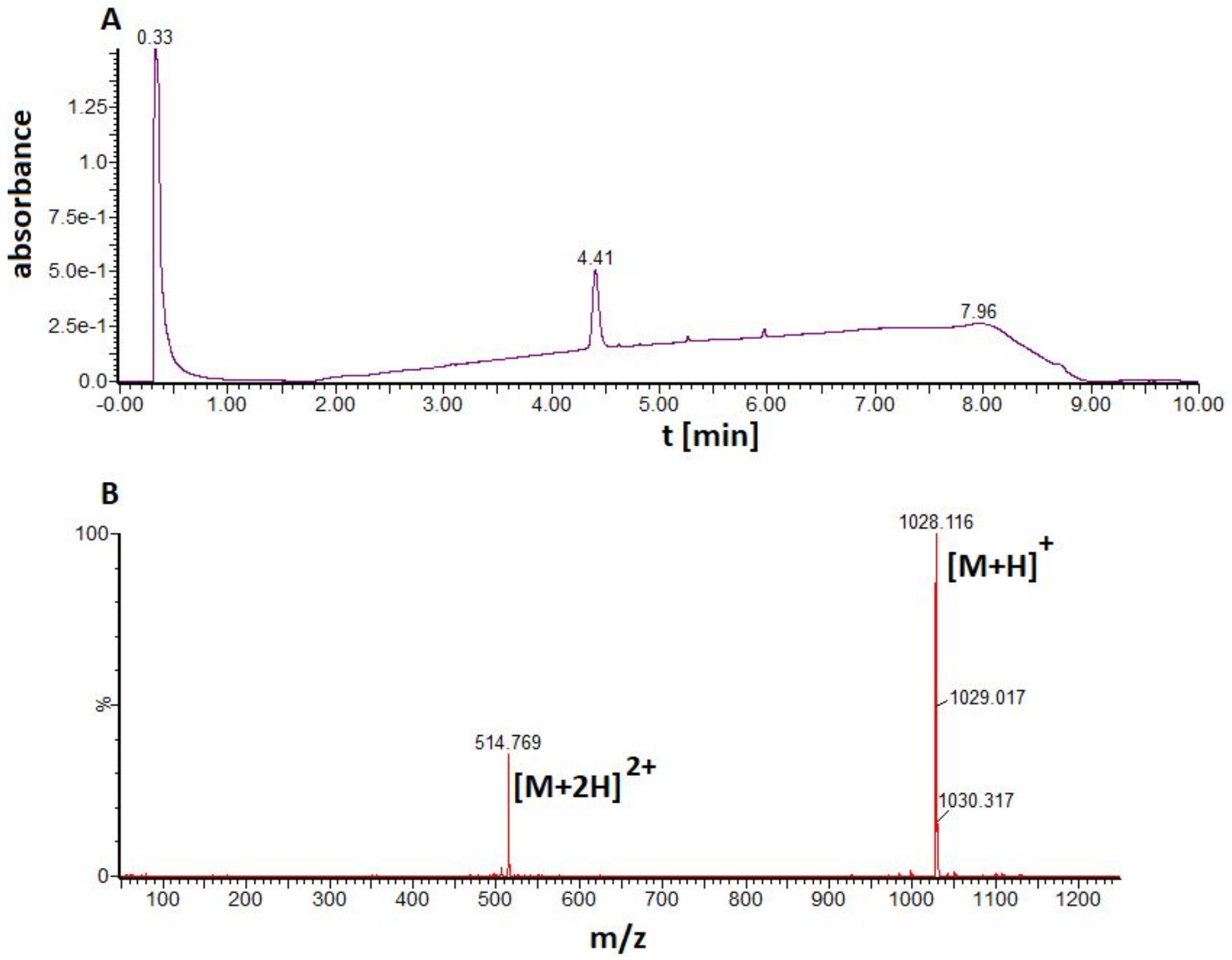

Figure S16: UPLC-MS Spectra of compound **16**. **A**. The photometric analysis was done between 200 to 400 nm (here shown at 220 nm). **B**. Mass spectra of the peak at 4.41 min.

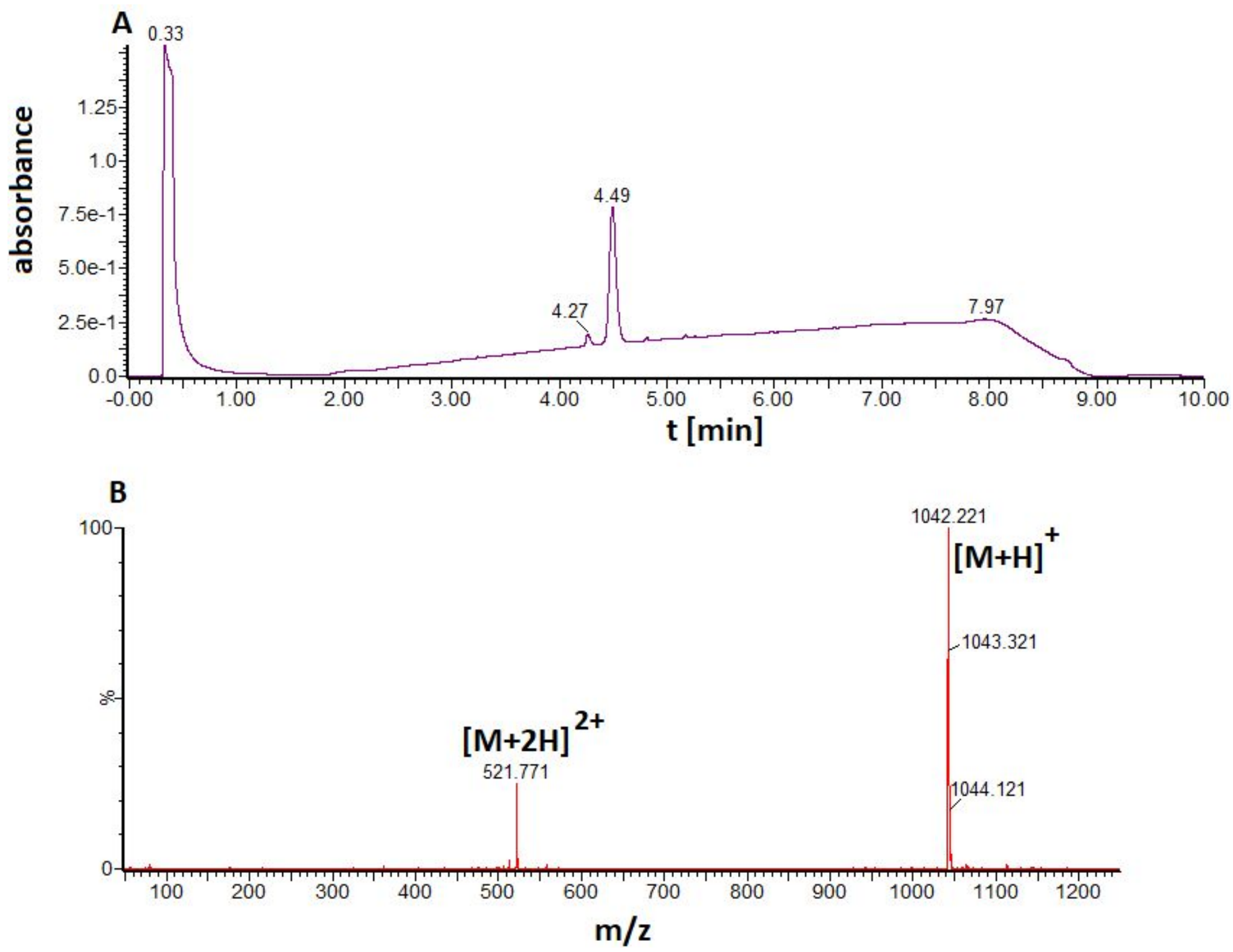

Figure S17: UPLC-MS Spectra of compound **17**. **A**. The photometric analysis was done between 200 to 400 nm (here shown at 220 nm). **B**. Mass spectra of the peak at 4.49 min.

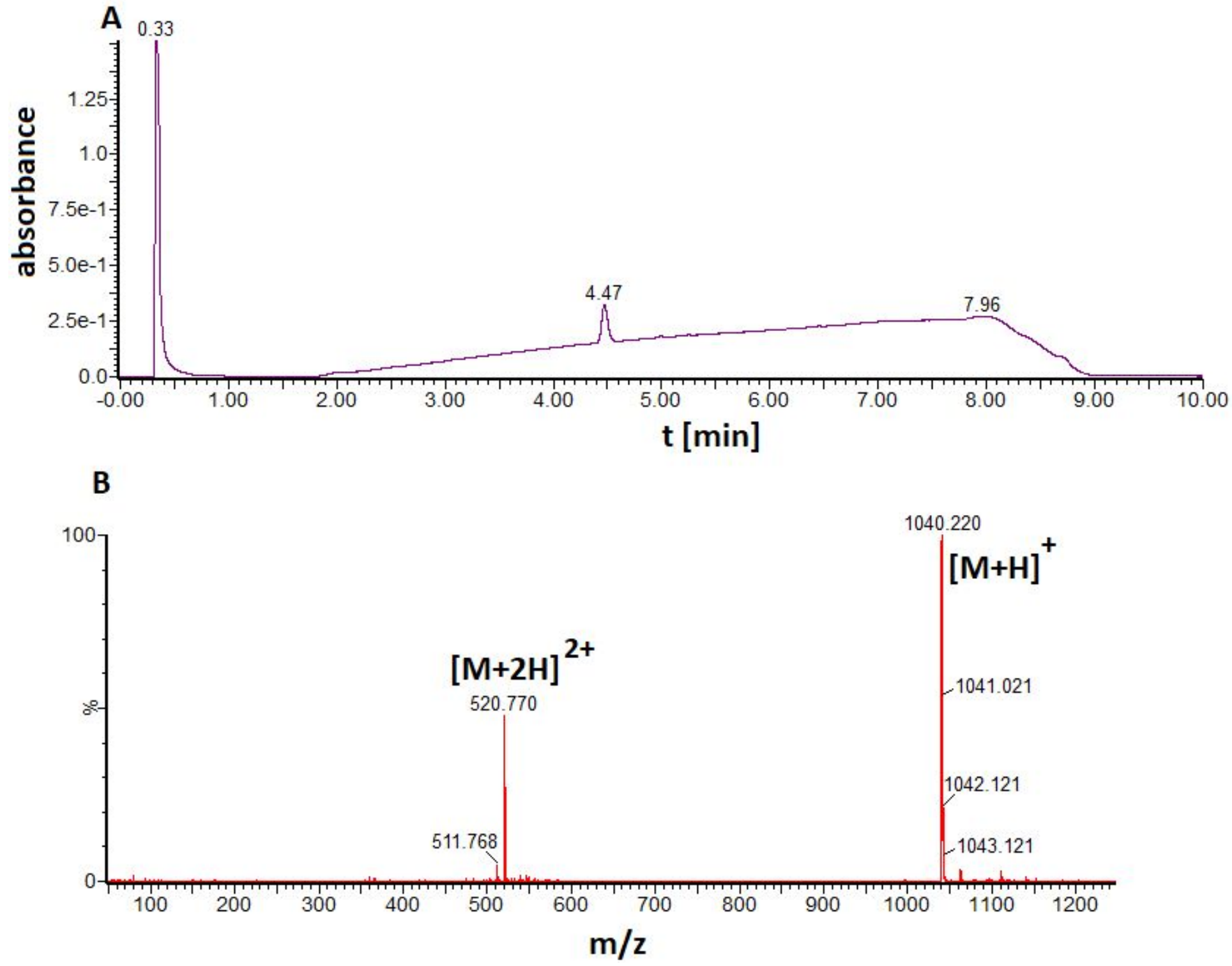

Figure S18: UPLC-MS Spectra of compound **18**. **A**. The photometric analysis was done between 200 to 400 nm (here shown at 220 nm). **B**. Mass spectra of the peak at 4.47 min.

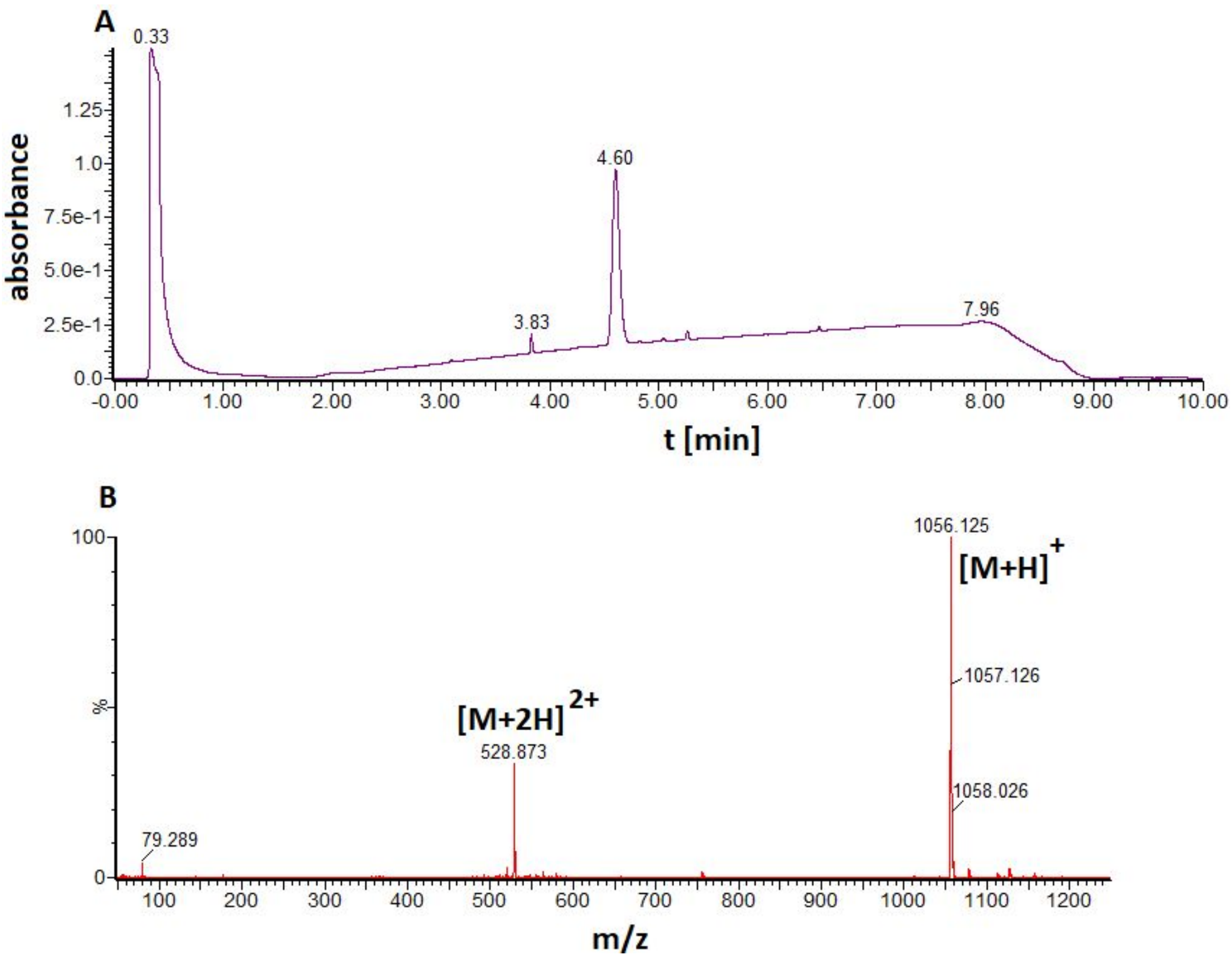

Figure S19: UPLC-MS Spectra of compound **19**. **A**. The photometric analysis was done between 200 to 400 nm (here shown at 220 nm). **B**. Mass spectra of the peak at 4.60 min.

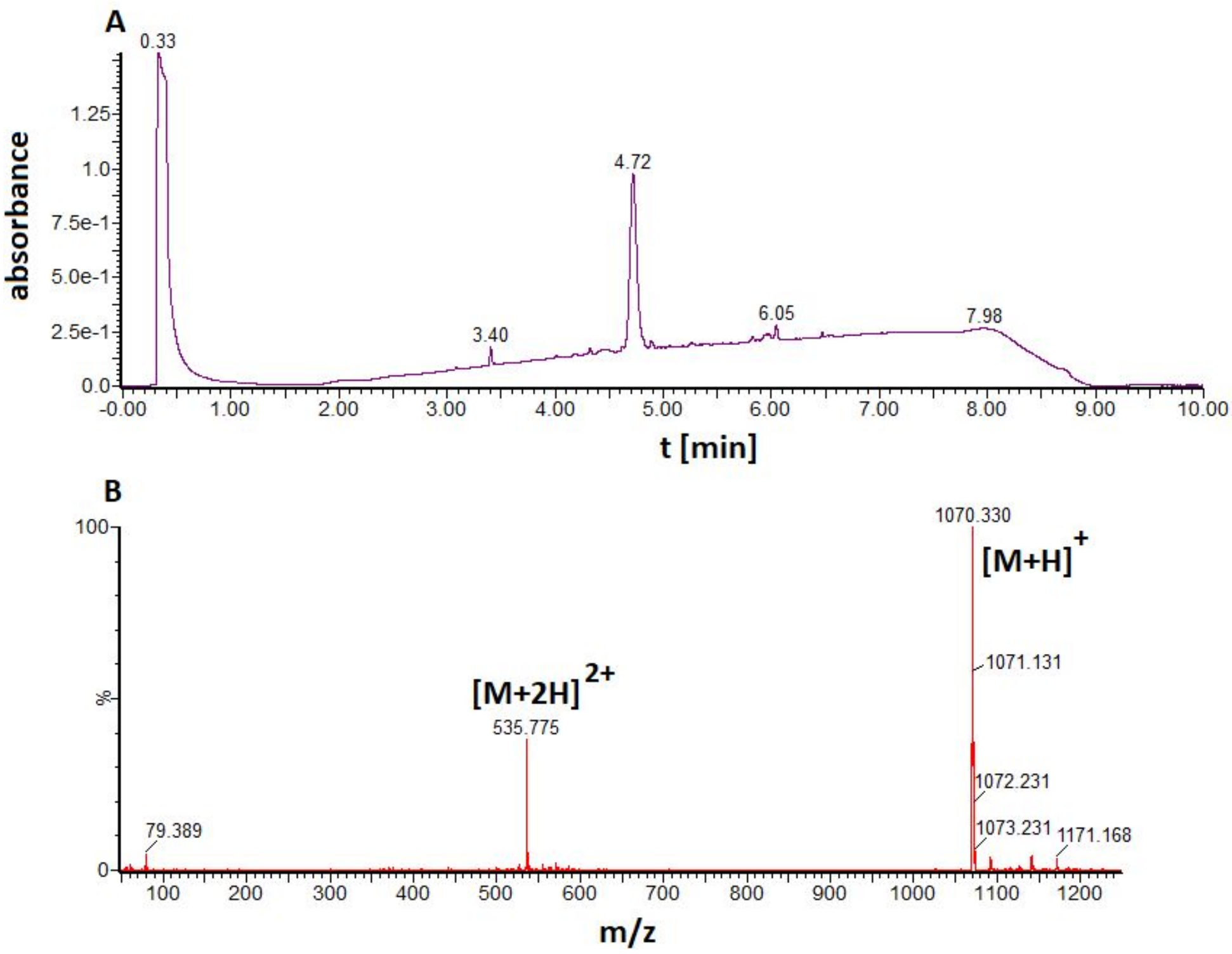

Figure S20: UPLC-MS Spectra of compound **20** **A**. The photometric analysis was done between 200 to 400 nm (here shown at 220 nm). **B**. Mass spectra of the peak at 4.72 min.

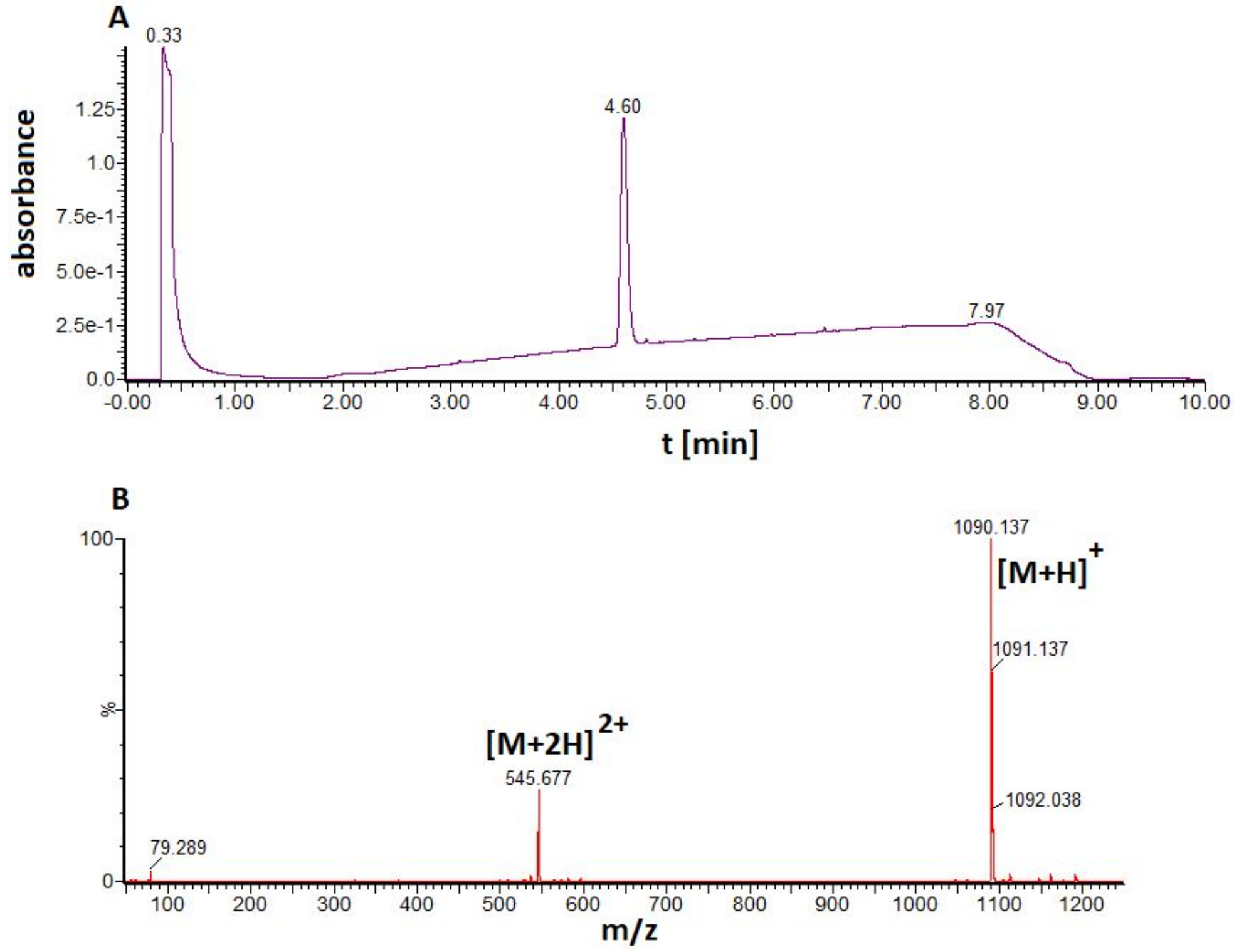

Figure S21: UPLC-MS Spectra of compound **21**. **A**. The photometric analysis was done between 200 to 400 nm (here shown at 220 nm). **B**. Mass spectra of the peak at 4.60 min.

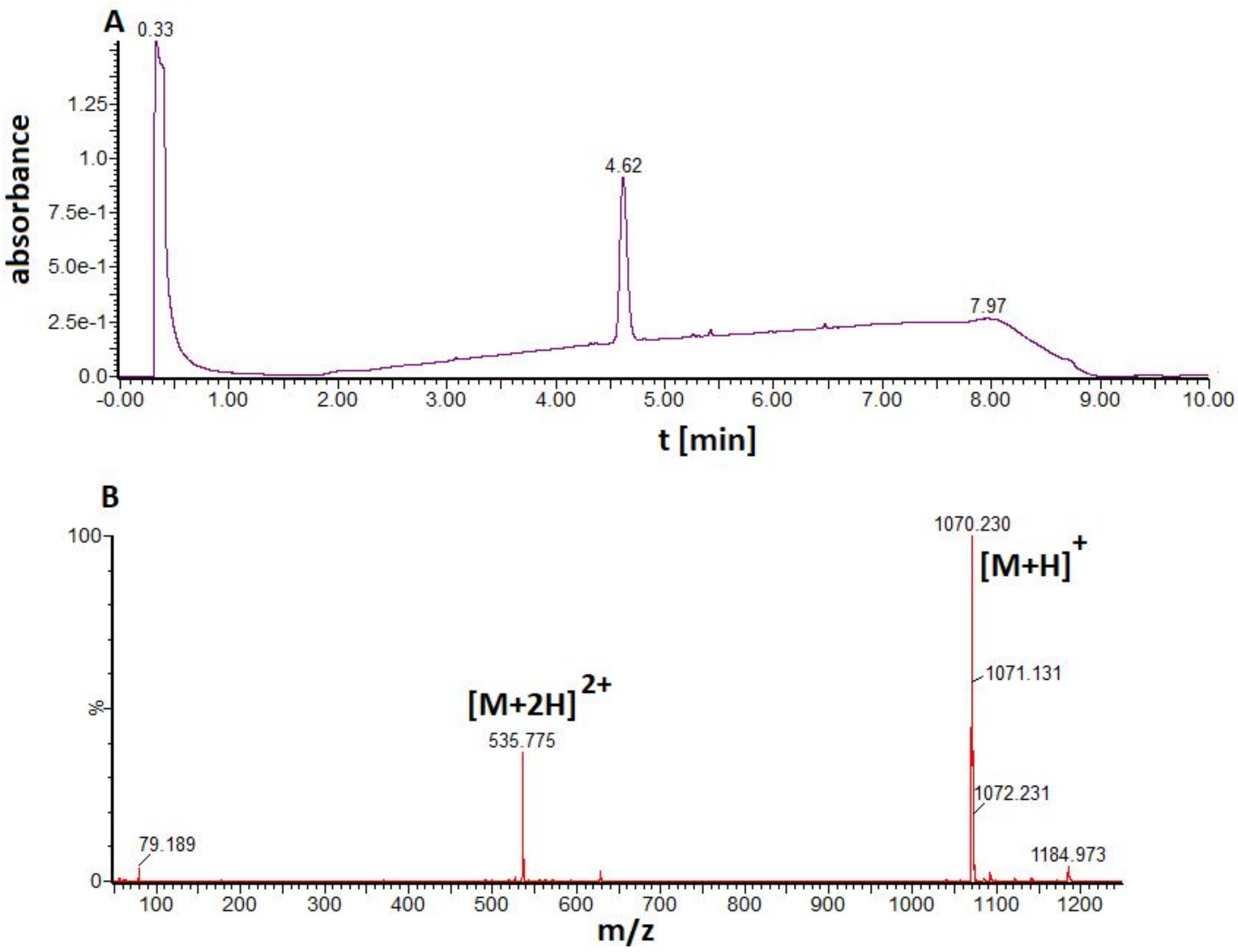

Figure S22: UPLC-MS Spectra of compound **22**. **A**. The photometric analysis was done between 200 to 400 nm (here shown at 220 nm). **B**. Mass spectra of the peak at 4.62 min.

B. UPLC-MS analysis of enzymatic deacylation experiments of the dually modified lysine peptides

50 µM of the corresponding peptide was incubated together with the corresponding enzyme in the buffer listed below with 5 % (*v/v*) DMSO, except for **6** where 15 % (*v/v*) DMSO was used, for 24 h at 30 °C. The high percentage of DMSO was proven to be not detrimental for enzyme activities (Figure S40). Class I, II and IV HDACs were used at 50 nM and SIRTs at 2 µM. The reactions were carried out in the following buffers: HDAC buffer (HDAC 1 – 9) contained 20 mM HEPES, 140 mM NaCl, 10 mM KCl, 0.1 mg ml^{-1} BSA, 1 mM TCEP, pH 7.4, HDAC10 buffer consisted of 20 mM HEPES, 0.1 mg ml^{-1} BSA, pH 7.4, HDAC11 buffer contained 20 mM HEPES, 0.5 mg ml^{-1} BSA, 70 µM TCEP, pH 7.4 and SIRT-buffer contained 20 mM Tris, 150 mM NaCl, 5 mM $MgCl_2$, 0.1 mg ml^{-1} BSA, 50 µM TCEP, 1 mM NAD^+, pH 7.8. Product formation was quantitatively evaluated by product peak area in the photometric analysis of the UPLC separation. Conversion less than 1 % after 24 h reaction with the respective HDAC was considered as not detectable by evaluation of absorbance. Absence of formed product was confirmed by mass spectra scans for the presumable cleavage product.

*B.1. Analysis of the peptide derivative H4K5AcMe (**4**)*

HDAC1

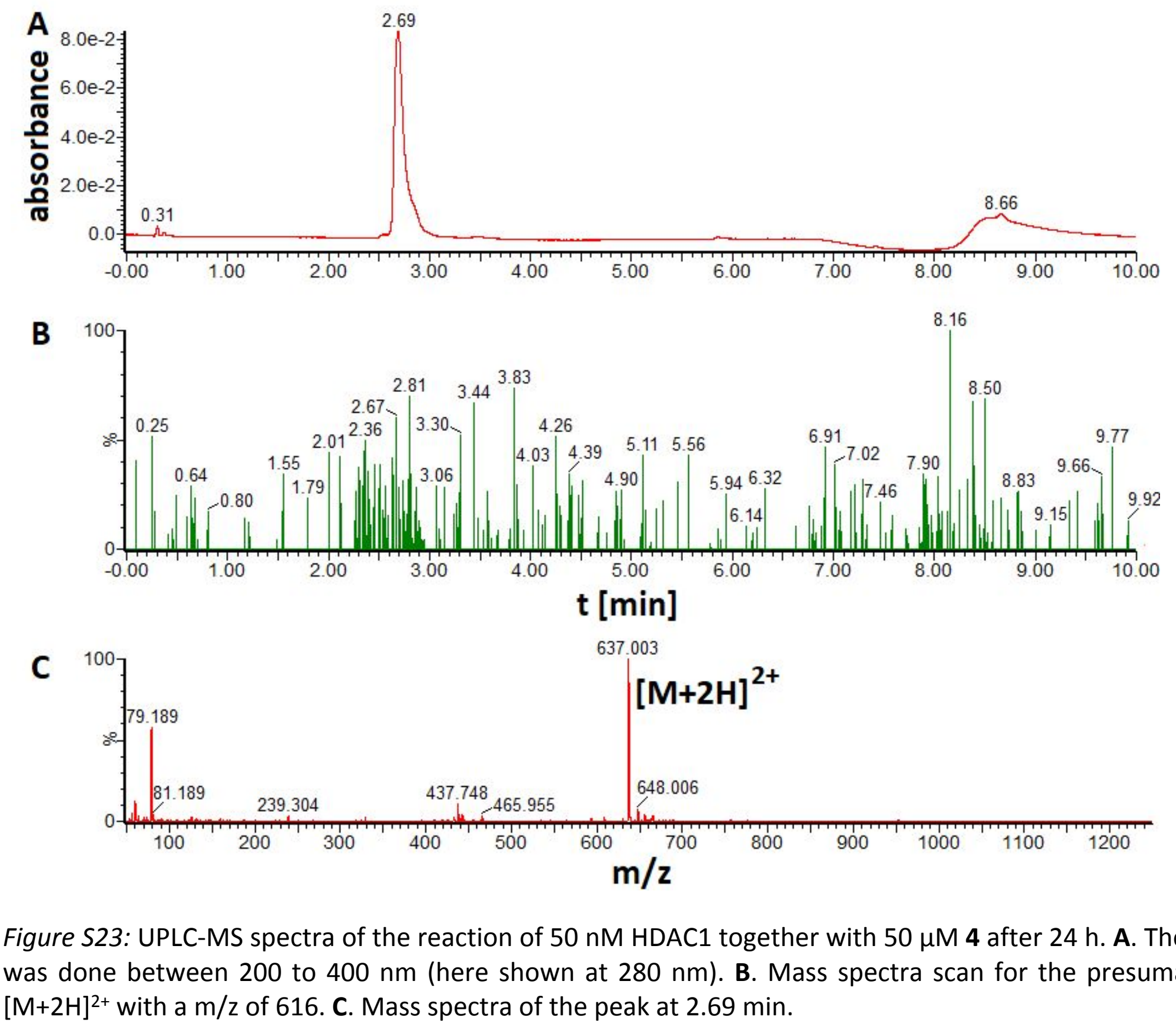

Figure S23: UPLC-MS spectra of the reaction of 50 nM HDAC1 together with 50 µM **4** after 24 h. **A**. The photometric analysis was done between 200 to 400 nm (here shown at 280 nm). **B**. Mass spectra scan for the presumable cleavage product $[M+2H]^{2+}$ with a m/z of 616. **C**. Mass spectra of the peak at 2.69 min.

HDAC2

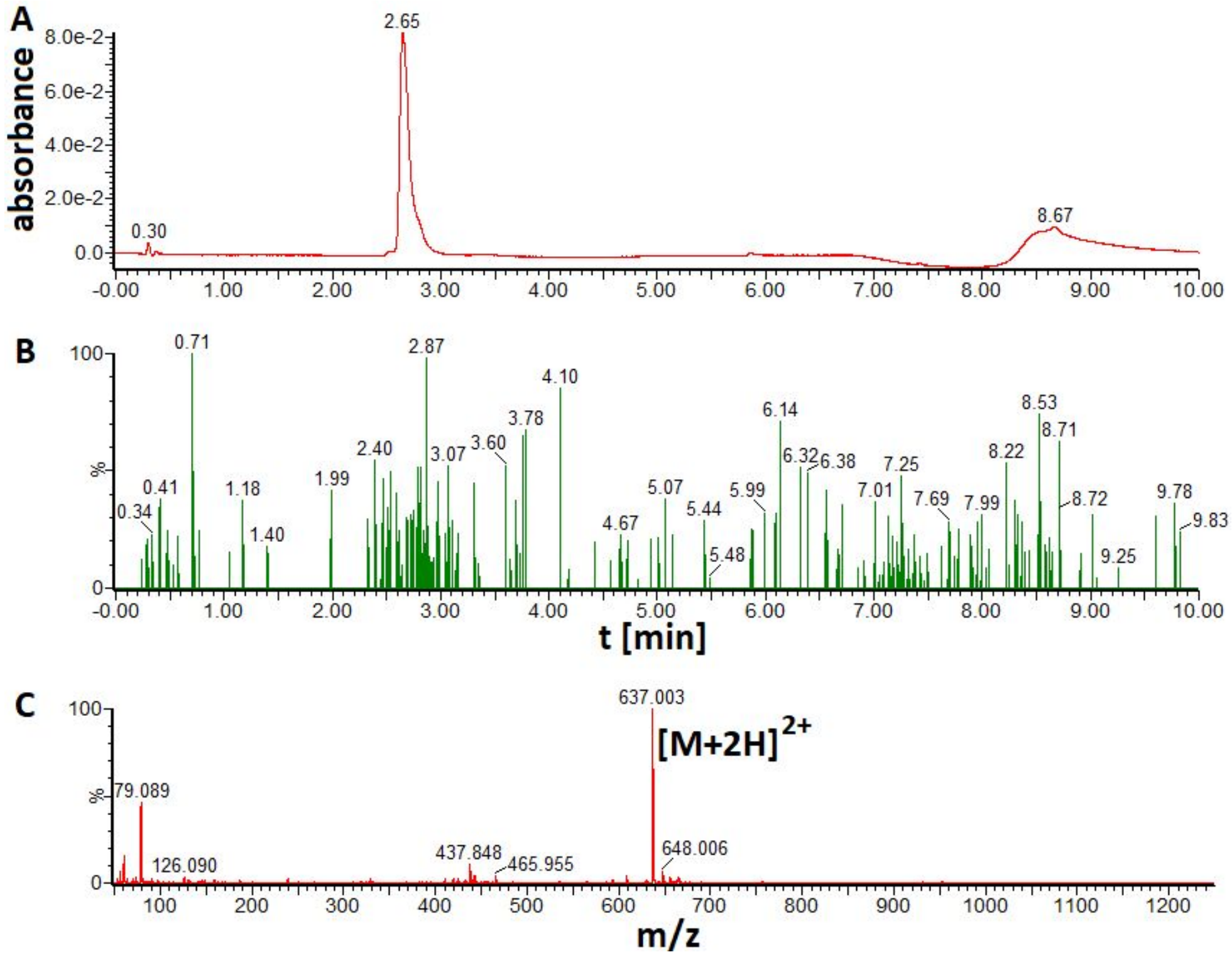

Figure S24: UPLC-MS spectra of the reaction of 50 nM HDAC2 together with 50 µM **4** after 24 h. **A**. The photometric analysis was done between 200 to 400 nm (here shown at 280 nm). **B**. Mass spectra scan for the presumable cleavage product $[M+2H]^{2+}$ with a m/z of 616. **C**. Mass spectra of the peak at 2.65 min.

HDAC3

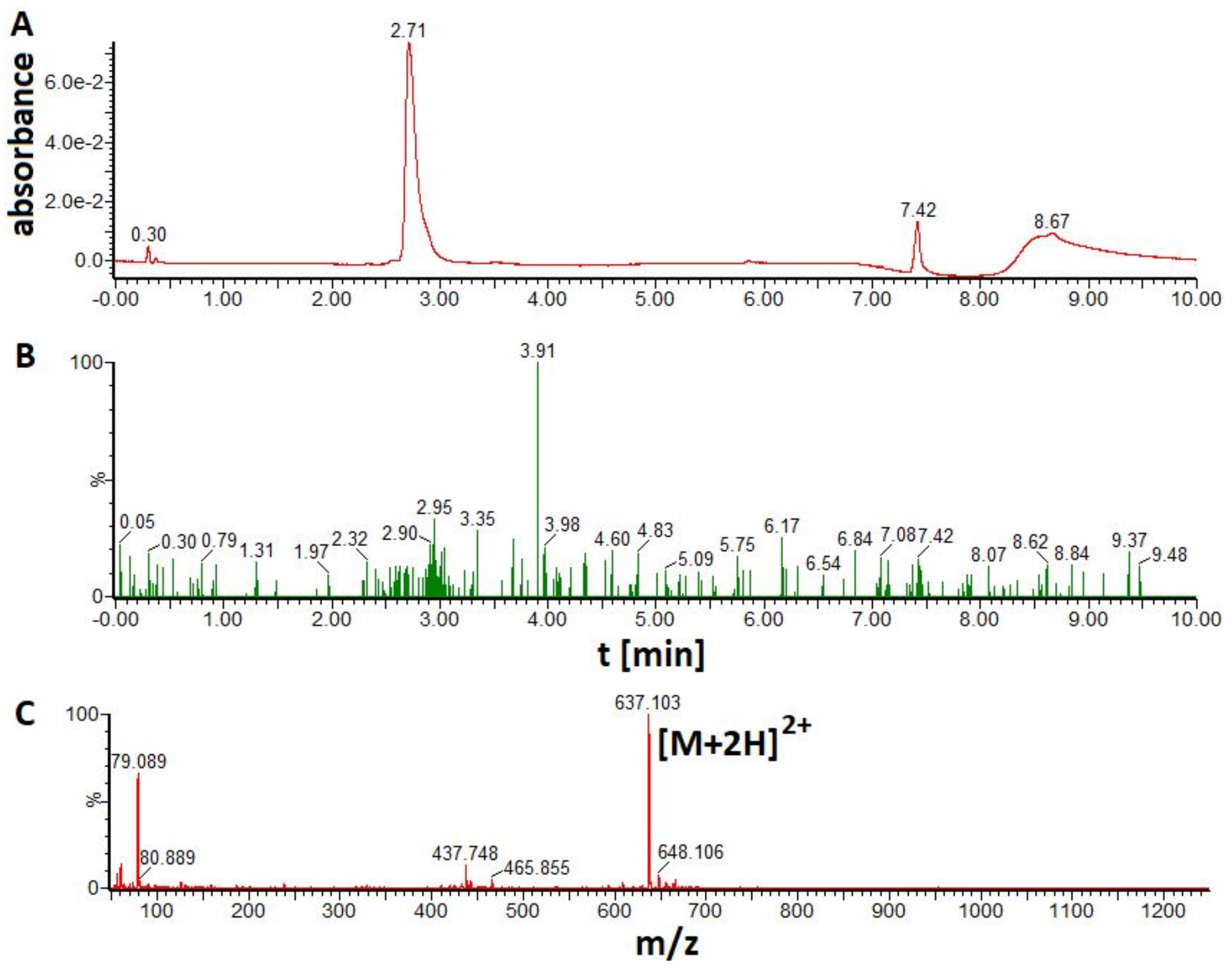

Figure S25: UPLC-MS spectra of the reaction of 50 nM HDAC3 together with 50 µM **4** after 24 h. **A**. The photometric analysis was done between 200 to 400 nm (here shown at 280 nm). **B**. Mass spectra scan for the presumable cleavage product $[M+2H]^{2+}$ with a m/z of 616. **C**. Mass spectra of the peak at 2.71 min.

HDAC6

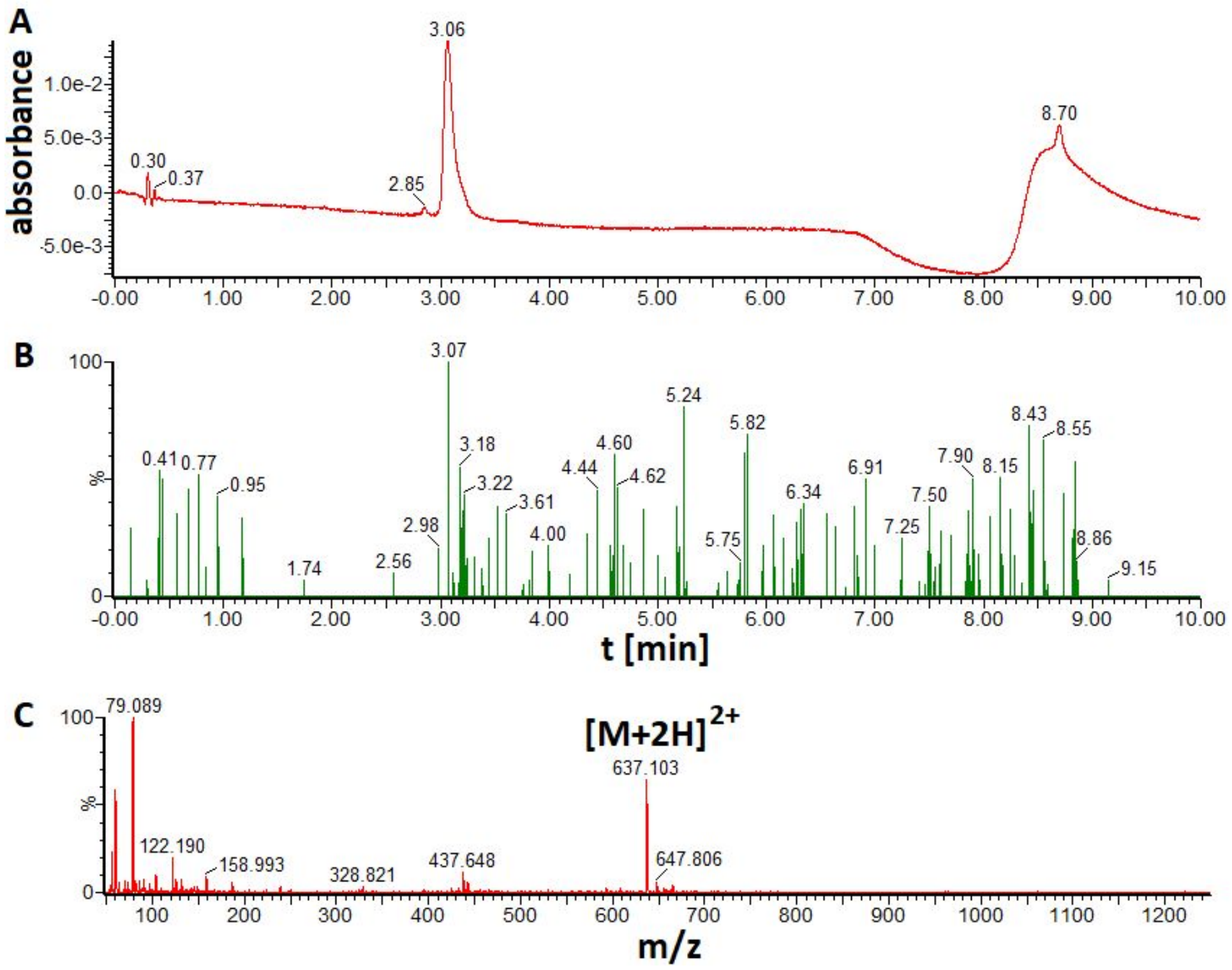

Figure S26: UPLC-MS spectra of the reaction of 50 nM HDAC6 together with 50 µM **4** after 24 h. **A**. The photometric analysis was done between 200 to 400 nm (here shown at 280 nm). **B**. Mass spectra scan for the presumable cleavage product $[M+2H]^{2+}$ with a m/z of 616. **C**. Mass spectra of the peak at 3.06 min.

SIRT1

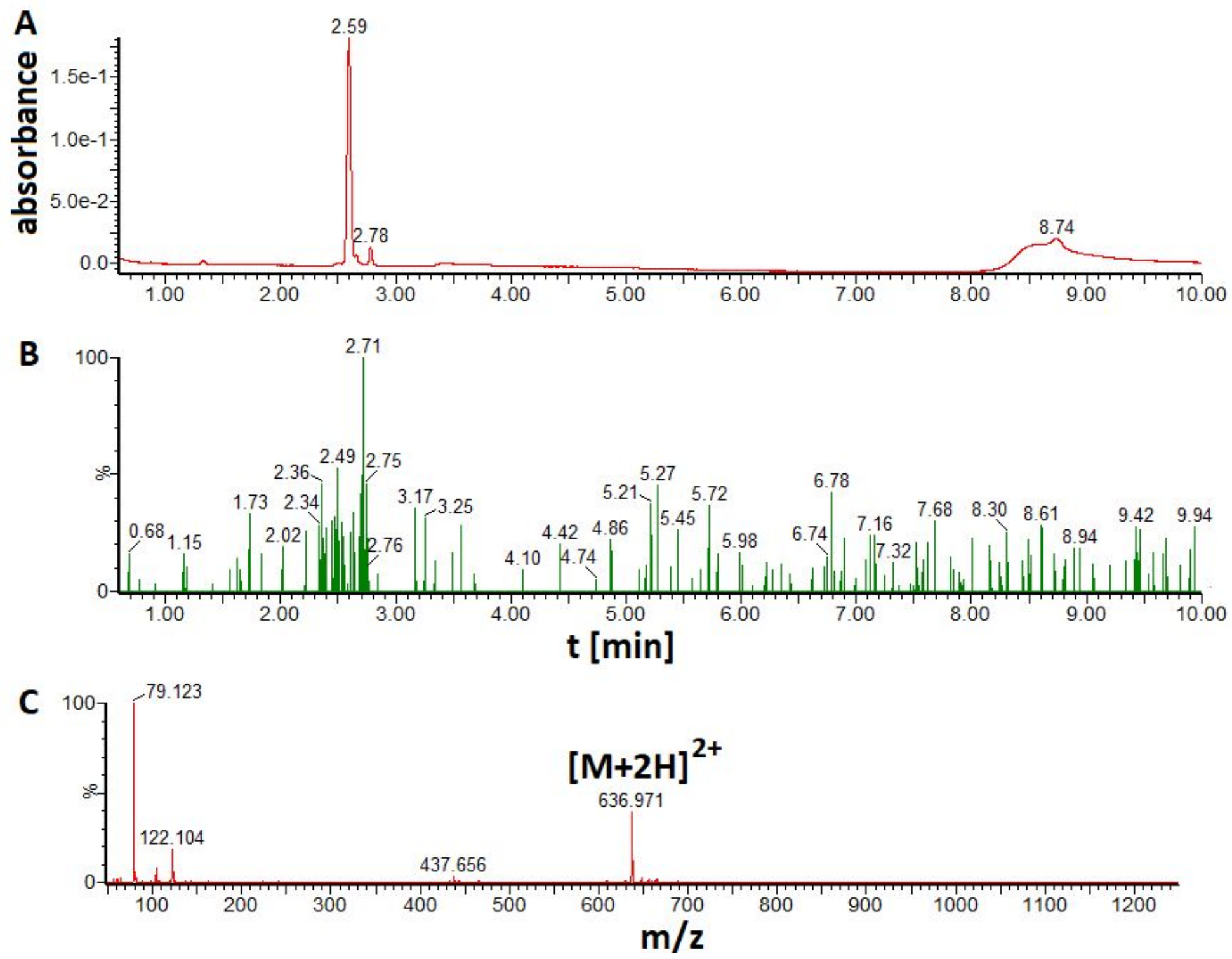

Figure S27: UPLC-MS spectra of the reaction of 2 µM SIRT1 together with 50 µM **4** after 24 h. **A**. The photometric analysis was done between 200 to 400 nm (here shown at 280 nm). **B**. Mass spectra scan for the presumable cleavage product $[M+2H]^{2+}$ with a m/z of 616. **C**. Mass spectra of the peak at 2.59 min.

SIRT2

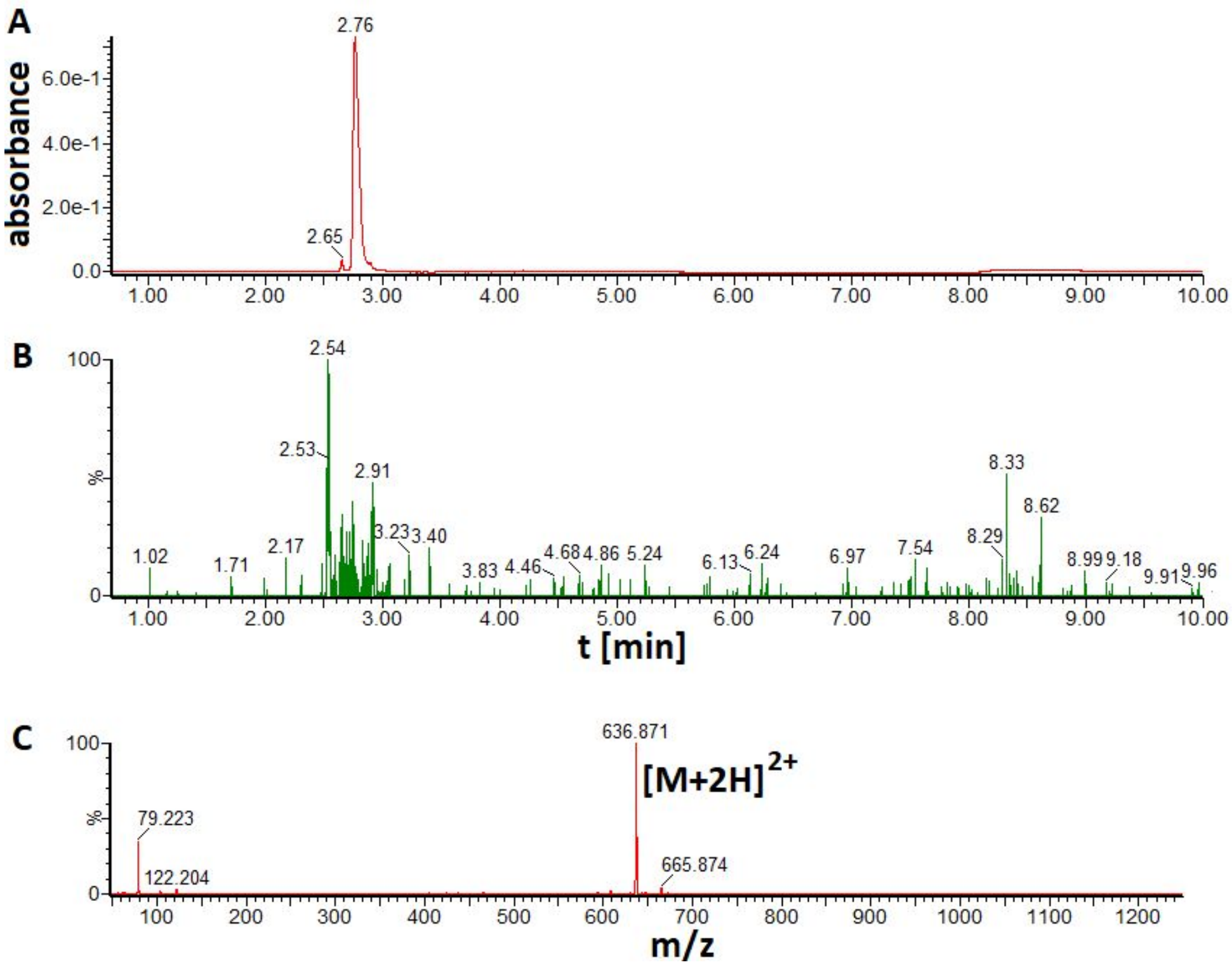

Figure S28: UPLC-MS spectra of the reaction of 2 µM SIRT2 together with 50 µM **4** after 24 h. **A**. The photometric analysis was done between 200 to 400 nm (here shown at 280 nm). **B**. Mass spectra scan for the presumable cleavage product $[M+2H]^{2+}$ with a m/z of 616. **C**. Mass spectra of the peak at 2.76 min.

SIRT3

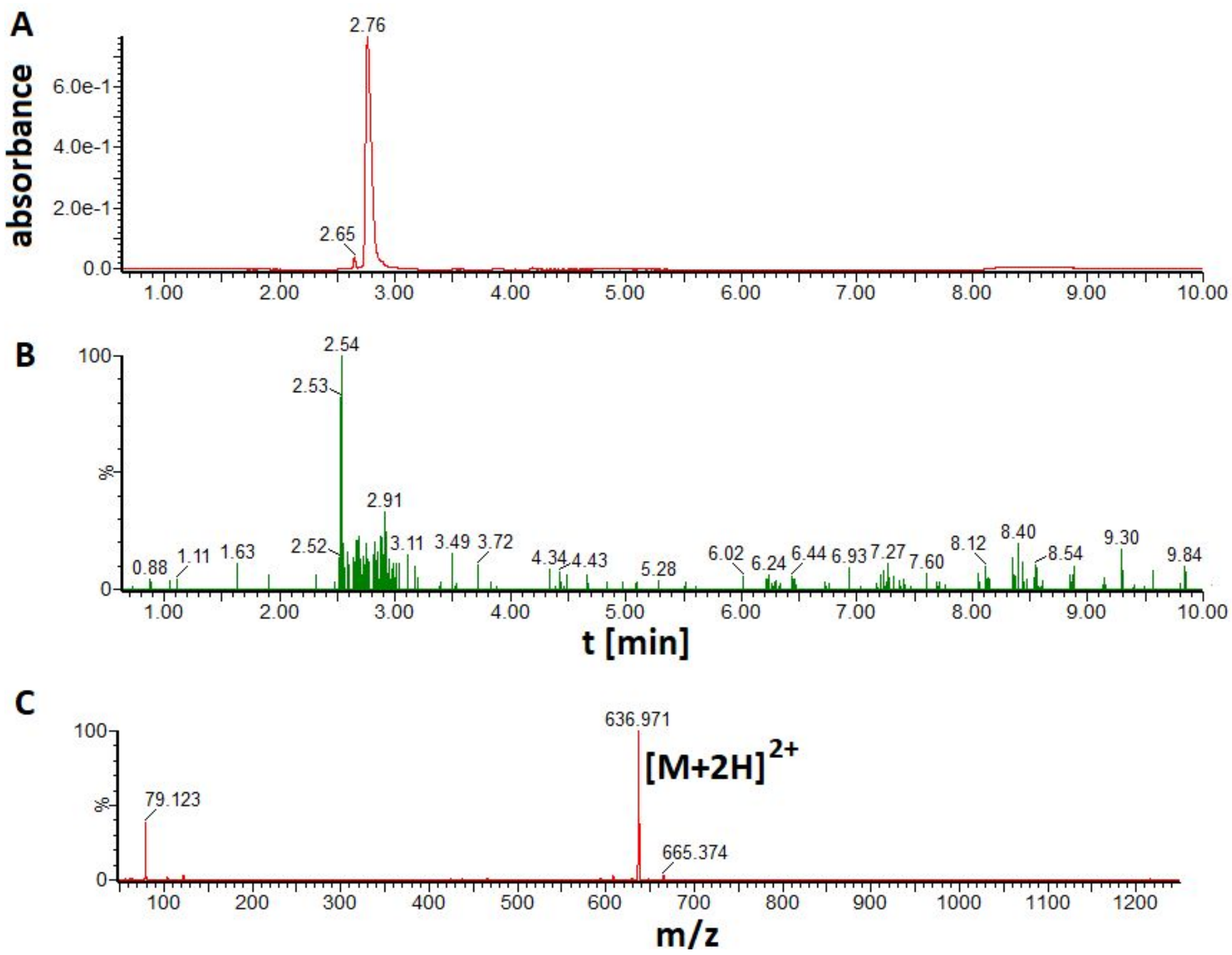

Figure S29: UPLC-MS spectra of the reaction of 2 µM SIRT3 together with 50 µM **4** after 24 h. **A**. The photometric analysis was done between 200 to 400 nm (here shown at 280 nm). **B**. Mass spectra scan for the presumable cleavage product $[M+2H]^{2+}$ with a m/z of 616. **C**. Mass spectra of the peak at 2.76 min.

SIRT6

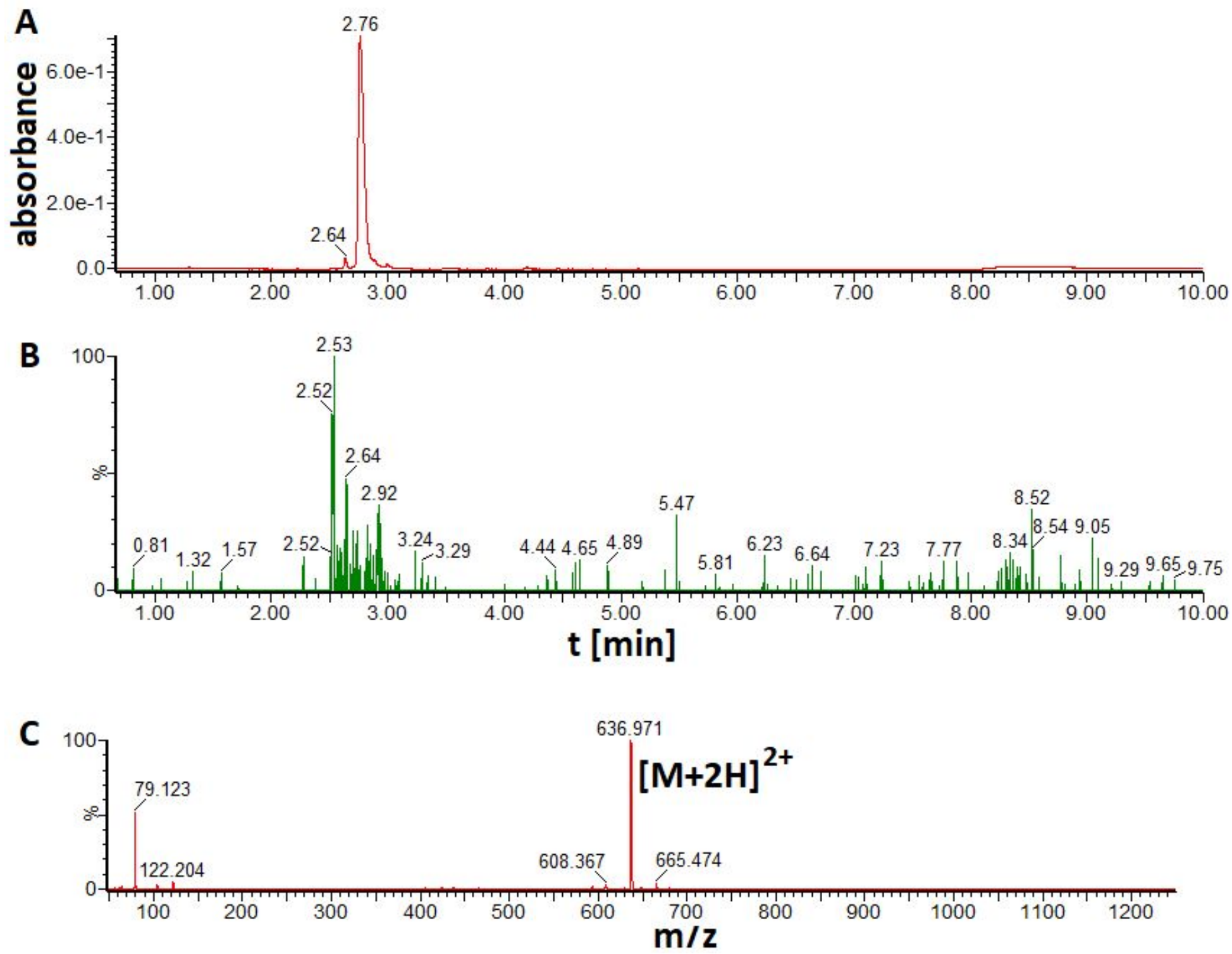

Figure S30: UPLC-MS spectra of the reaction of 5 µM SIRT6 together with 50 µM **4** after 24 h. **A**. The photometric analysis was done between 200 to 400 nm (here shown at 280 nm). **B**. Mass spectra scan for the presumable cleavage product $[M+2H]^{2+}$ with a m/z of 616. **C**. Mass spectra of the peak at 2.59 min. **D**. Mass spectra of the peak at 2.76 min.

*B.2. Analysis of the peptide derivative H4K5MyrMe (**6**)*

HDAC8

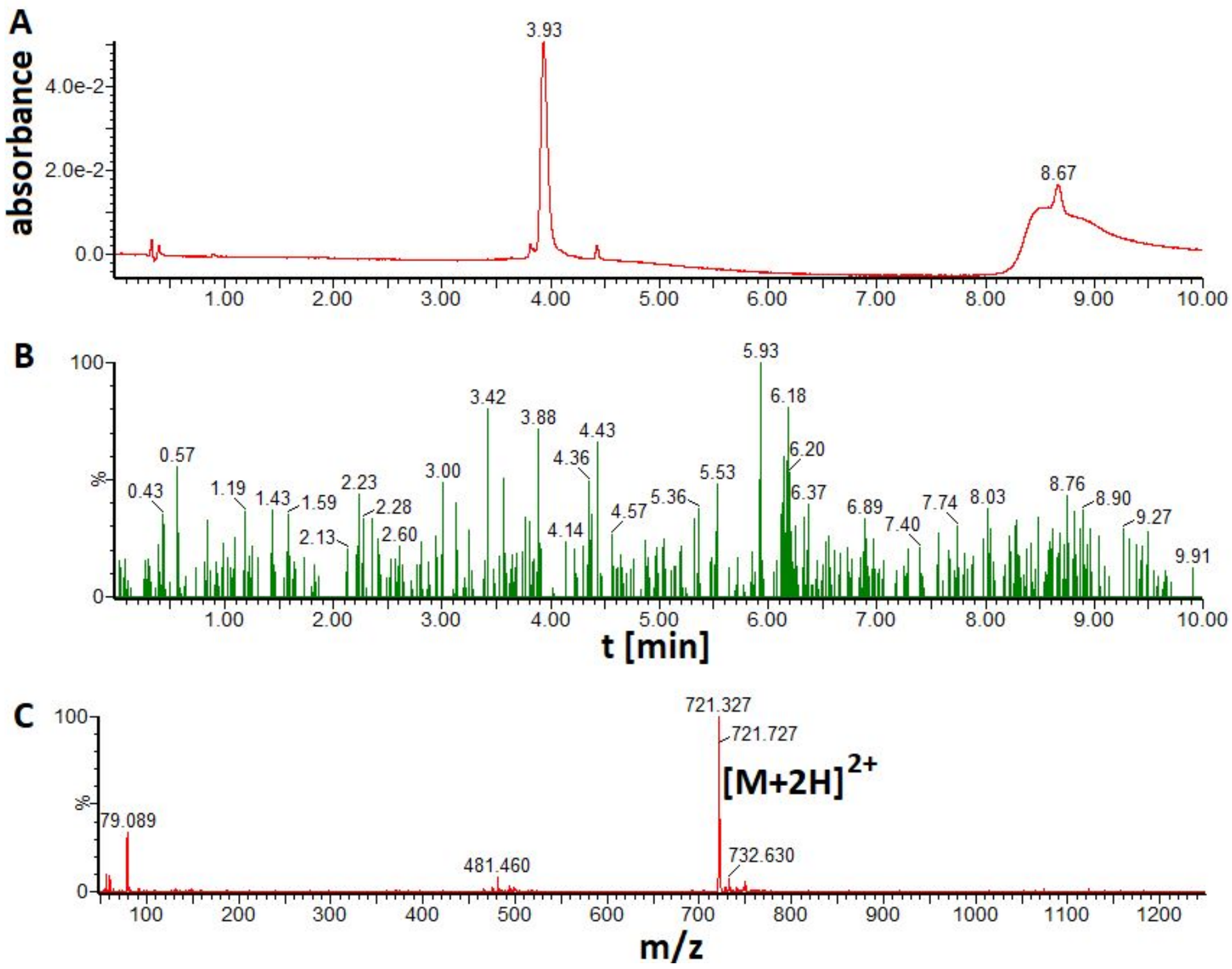

Figure S31: UPLC-MS spectra of the reaction of 50 nM HDAC8 together with 50 µM **6** after 24 h. **A**. The photometric analysis was done between 200 to 400 nm (here shown at 280 nm). **B**. Mass spectra scan for the presumable cleavage product $[M+2H]^{2+}$ with a m/z of 616. **C**. Mass spectra of the peak at 3.93 min.

HDAC11

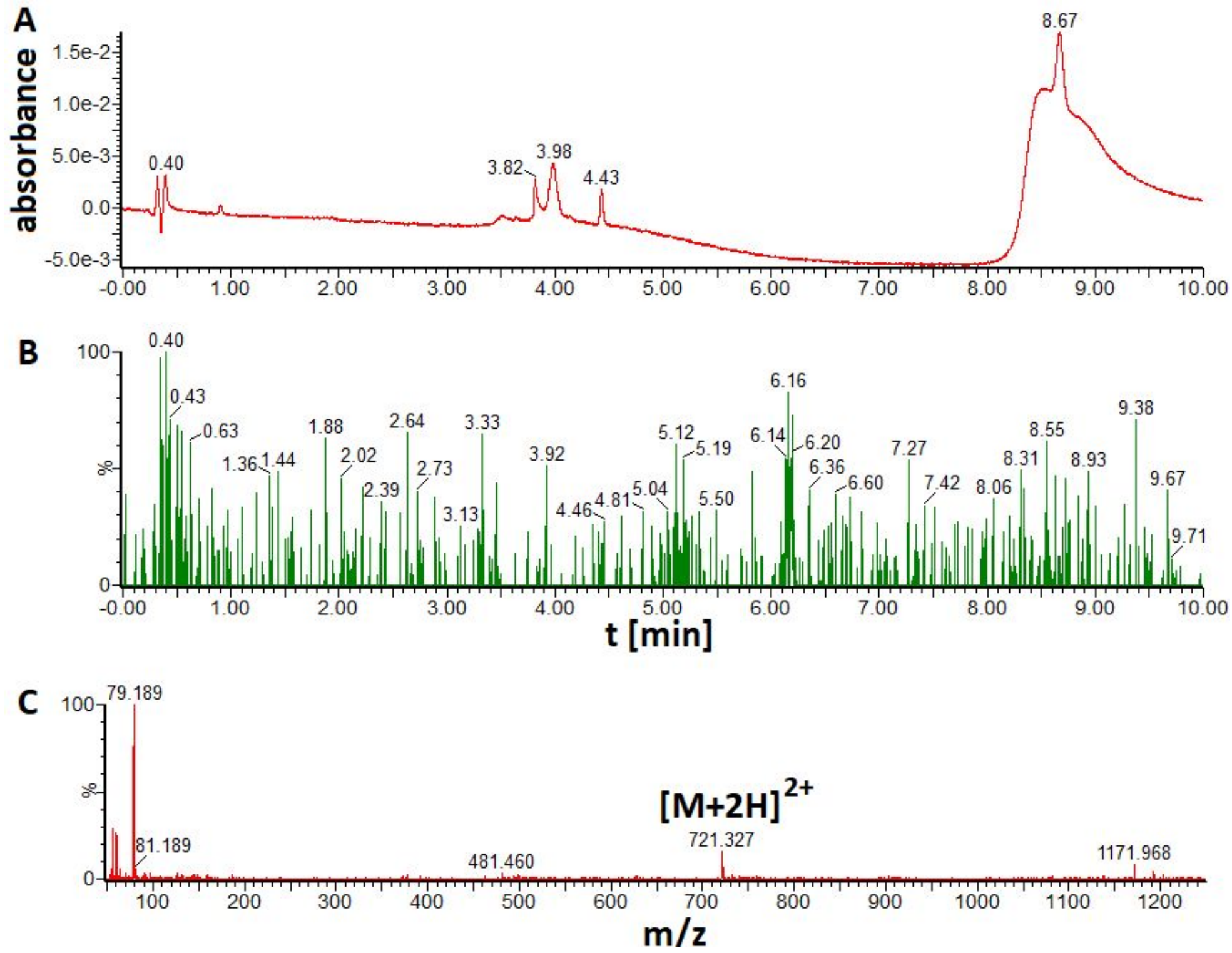

Figure S32: UPLC-MS spectra of the reaction of 50 nM HDAC11 together with 50 µM **6** after 24 h. **A**. The photometric analysis was done between 200 to 400 nm (here shown at 280 nm). **B**. Mass spectra scan for the presumable cleavage product $[M+2H]^{2+}$ with a m/z of 616. **C**. Mass spectra of the peak at 3.98 min.

SIRT2

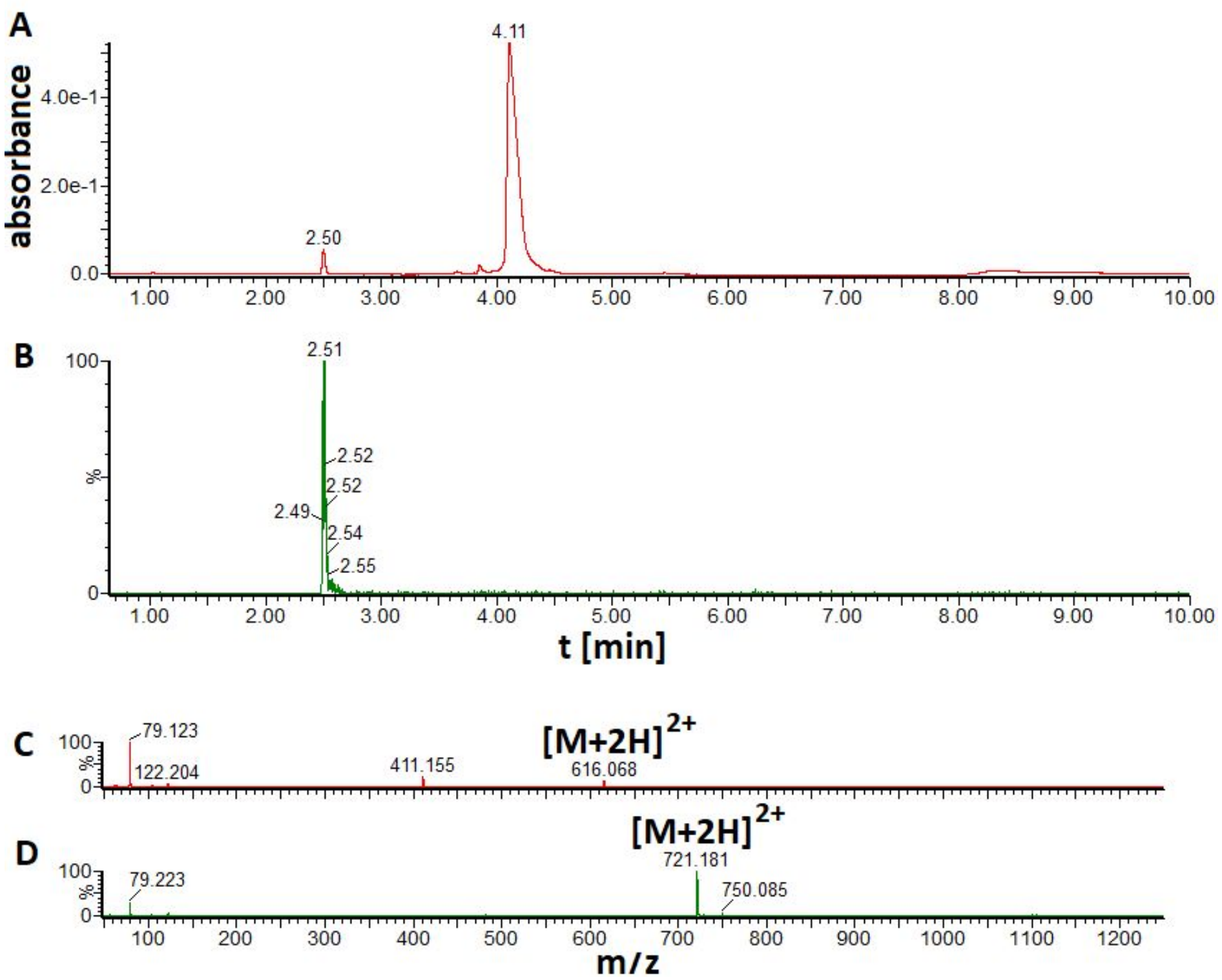

Figure S33: UPLC-MS spectra of the reaction of 2 µM SIRT2 together with 50 µM **6** after 24 h. **A**. The photometric analysis was done between 200 to 400 nm (here shown at 280 nm). **B**. Mass spectra scan for the presumable cleavage product $[M+2H]^{2+}$ with a m/z of 616. **C**. Mass spectra of the peak at 2.50 min. **D**. Mass spectra of the peak at 4.11 min.

SIRT3

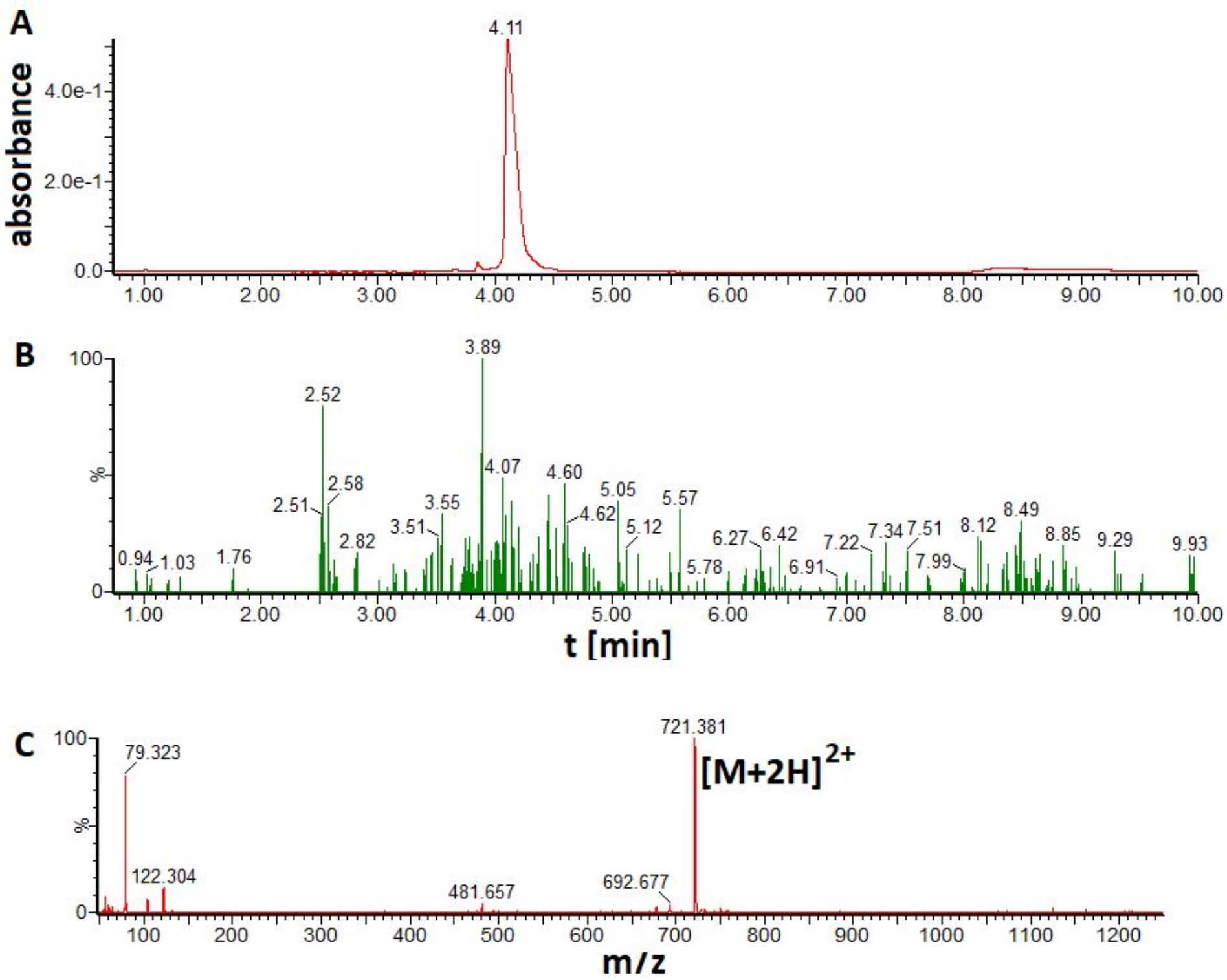

Figure S34: UPLC-MS spectra of the reaction of 2 µM SIRT3 together with 50 µM **6** after 24 h. **A**. The photometric analysis was done between 200 to 400 nm (here shown at 280 nm). **B**. Mass spectra scan for the presumable cleavage product $[M+2H]^{2+}$ with a m/z of 616. **C**. Mass spectra of the peak at 4.11 min

SIRT6

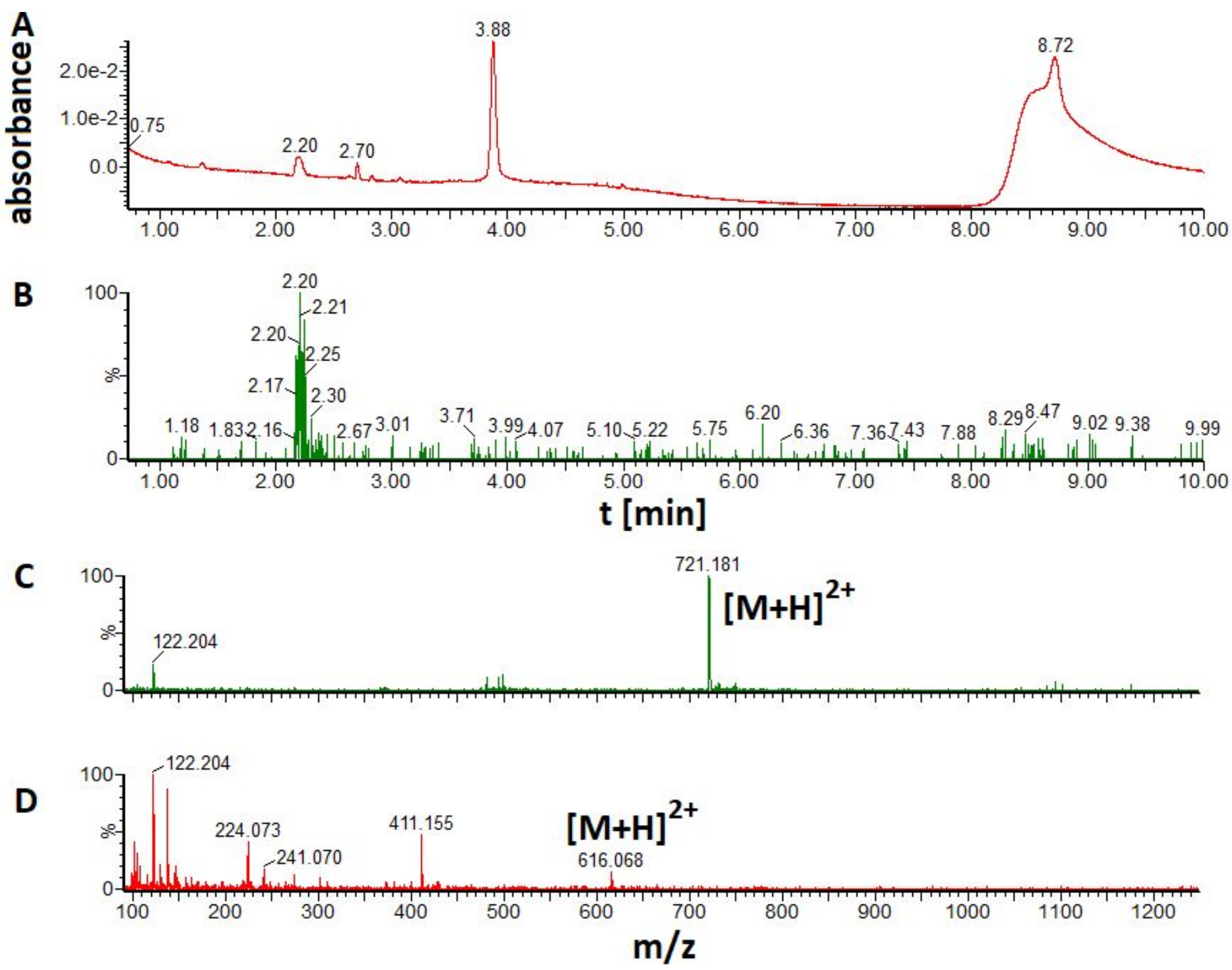

Figure S35: UPLC-MS spectra of the reaction of 2 µM SIRT6 together with 50 µM **6** after 24 h. **A**. The photometric analysis was done between 200 to 400 nm (here shown at 280 nm). **B**. Mass spectra scan for the presumable cleavage product $[M+2H]^{2+}$ with a m/z of 616 **C.** Mass spectra of the peak at 2.20 min. **D**. Mass spectra of the peak at 3.88 min.

C. Dose-response curves

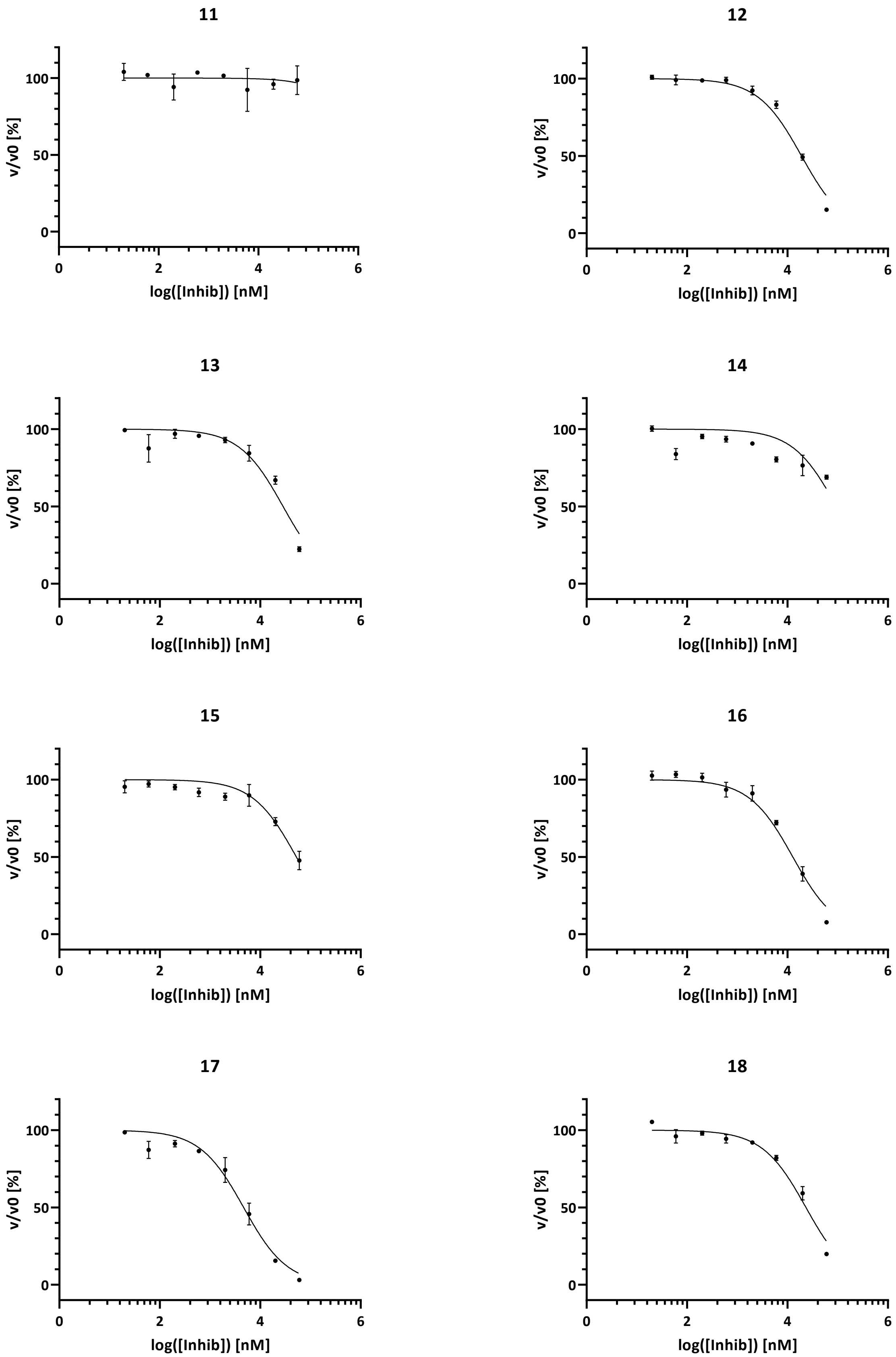

Figure S36: Dose-response analysis for the inhibitors **11** – **18** for HDAC11.

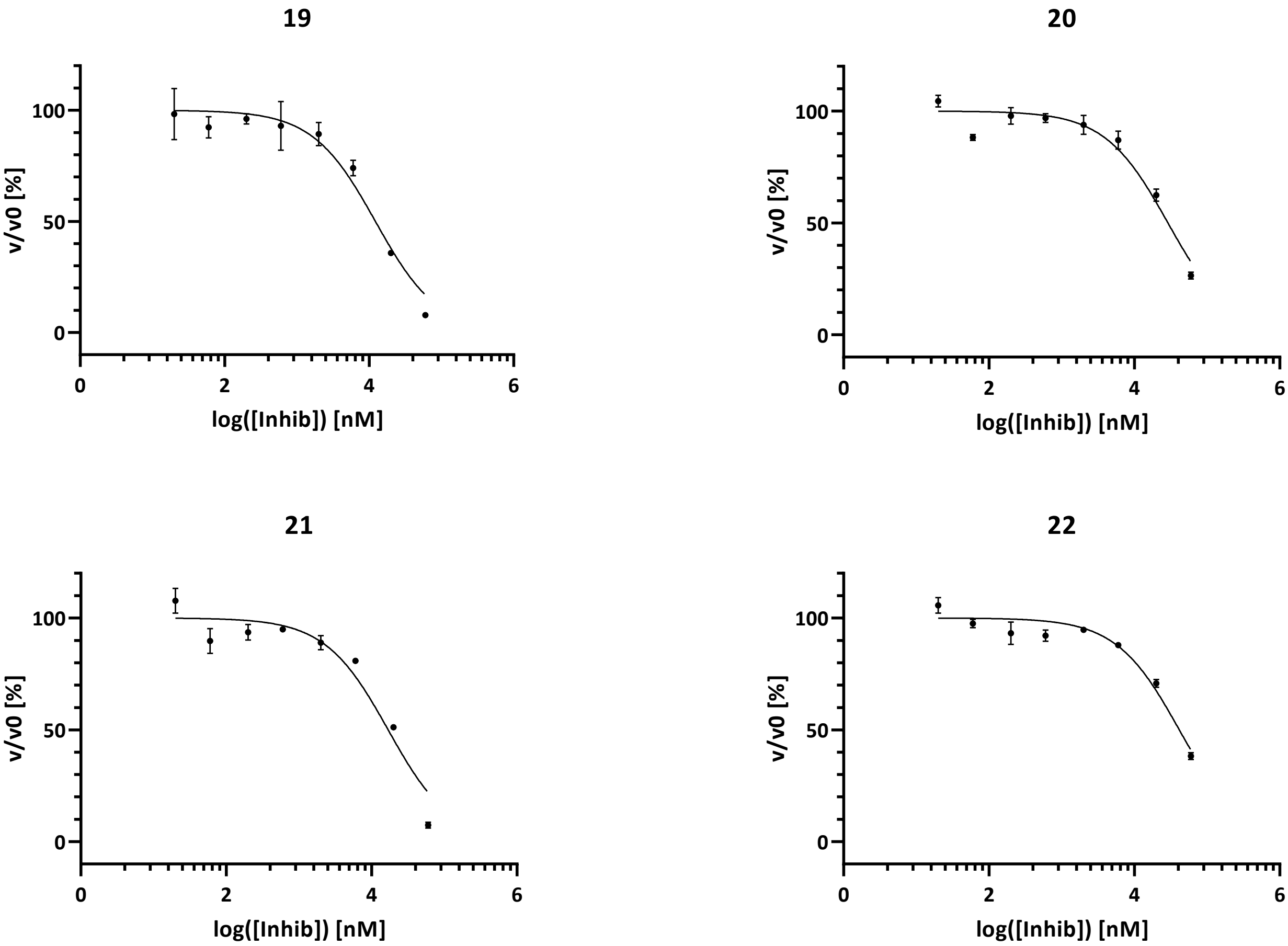

Figure S37: Dose-response analysis for the inhibitors **19** – **22** for HDAC11.

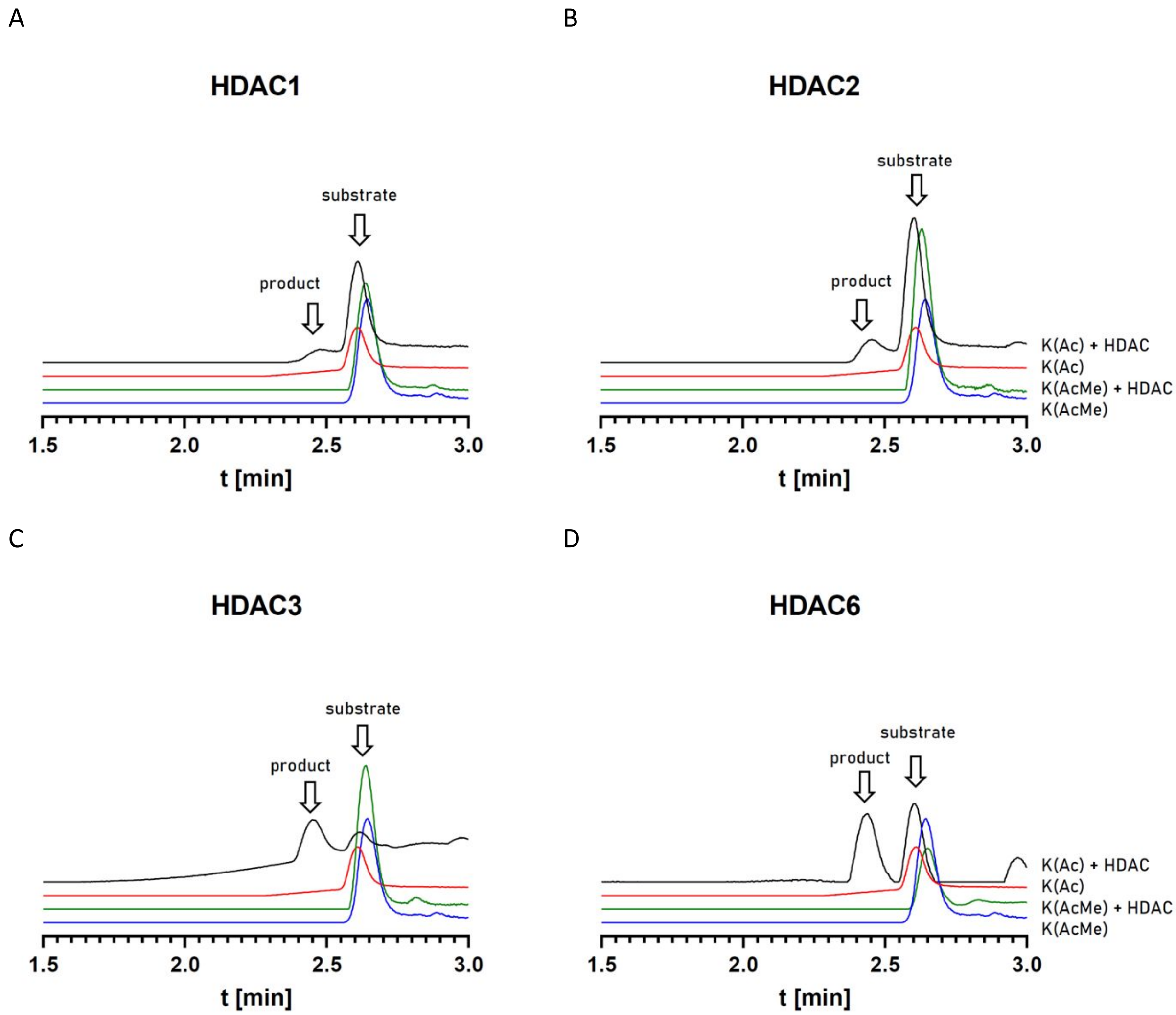

Figure S38: Comparison of the effect HDACs on KAc and KAcMe peptides. HPLC chromatograms from deacetylation reactions by HDAC1 (A), HDAC2 (B), HDAC3 (C) and HDAC6 (D). 100 µM of peptide **3** (H4K5(Ac)) (red, black) or **4** (H4K5AcMe) (green, blue) were incubated with 50 nM HDAC (red, green) or alone (black, blue) for 24 h at 30 °C. Analyses of the samples were performed by RP-HPLC using a C18 column and UV detection at 280 nm. The deacetylation of peptides caused elution at an earlier retention.

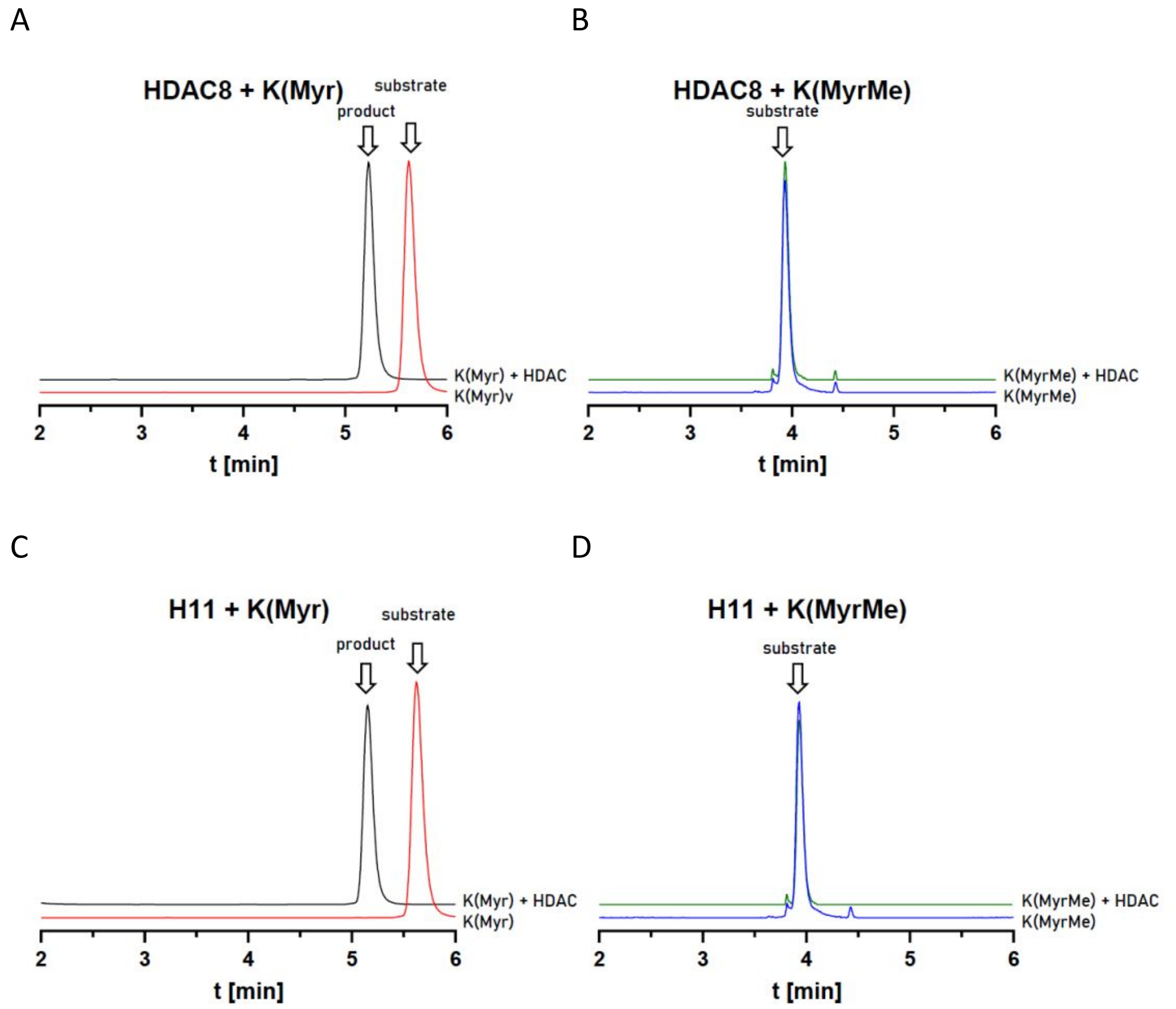

Figure S39: Comparison of the effect HDACs on KMyr and KMyrMe peptides. HPLC chromatograms from deacetylation reactions by HDAC8 (A, B) and HDAC11 (C, D). 100 µM of peptide **5** (H4K5(Myr)) (red, black) or **6** (H4K5(MyrMe)) (green, blue) were incubated with 50 nM HDAC (red, green) or alone (black, blue) for 24 h at 30 °C. Analyses of the samples were performed by RP-HPLC using a C18 column and UV detection at 280 nm. The deacetylation of peptides caused elution at an earlier retention.

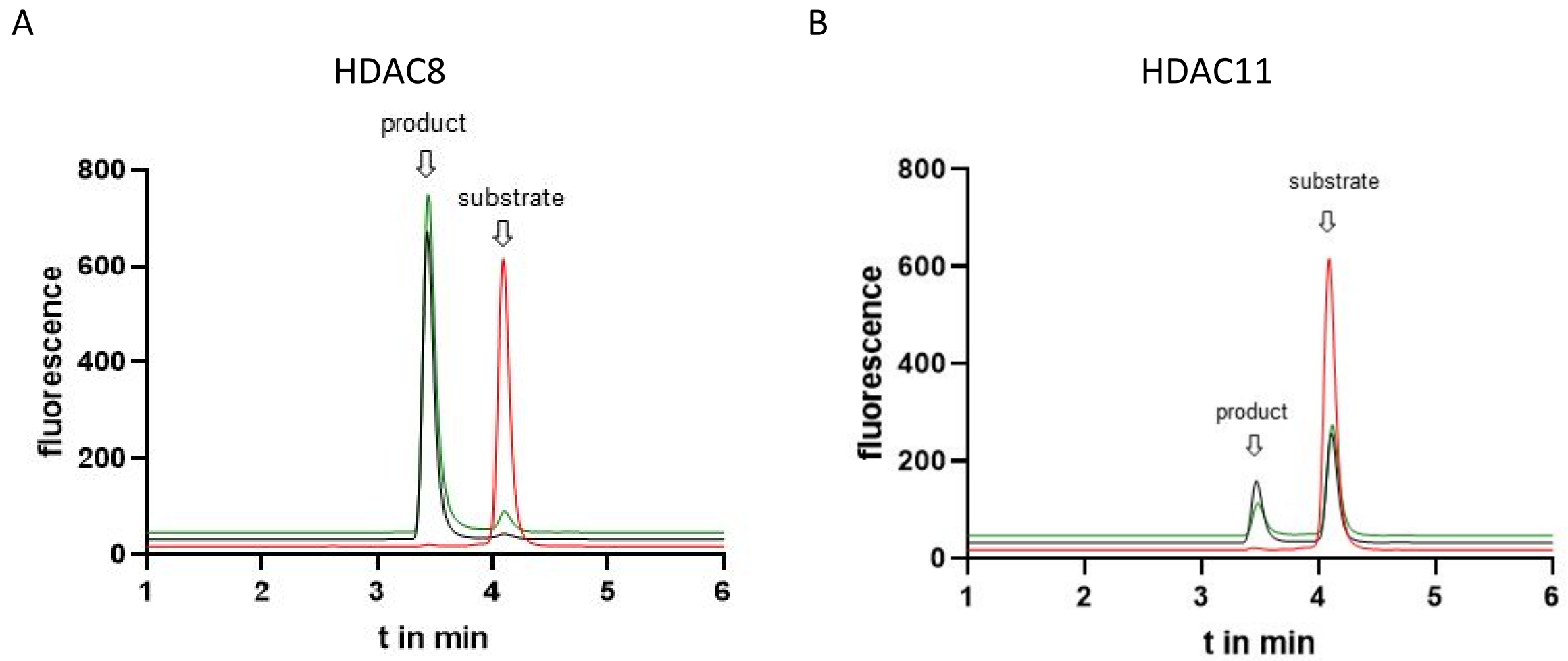

Figure S40: Enzymatic activity of HDAC8 and HDAC11 in the presence different amounts of DMSO. HPLC chromatograms from deacetylation reactions of peptide **S1** by HDAC8 (A) and HDAC11 (B). **A.** 500 µM of the peptide **S1** were incubated with 20 nM HDAC8 in the presence of 15 % DMSO (black, 96 % conversion) or 5 % DMSO as a control (green, 93 % conversion) or without HDAC (red) for 1 h at RT in HDAC buffer. **B.** 200 µM of the peptide **S1** were incubated with 200 nM HDAC11 in the presence of 15 % DMSO (black, 29 % conversion) or 5 % DMSO as a control (green, 23 % conversion) or without HDAC (red) for 14 h at RT in HDAC buffer. The deacetylation of peptides caused elution at an earlier retention. Analyses of the samples were performed by RP-HPLC using a C18 column and fluorescence detection with excitation at 320 nm and emission at 420 nm. 5 % DMSO was standardly used in all deacylation reactions performed.

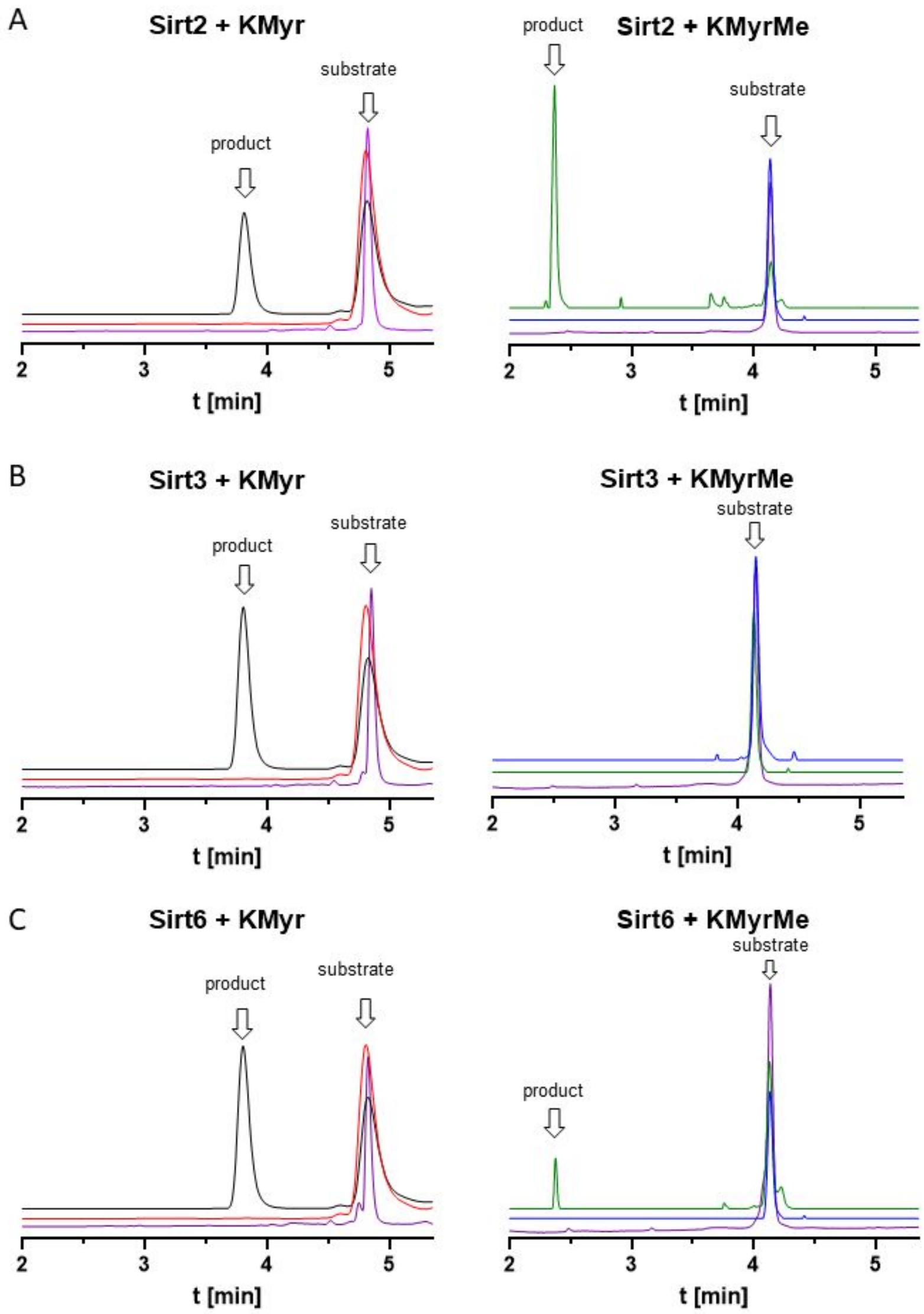

Figure S41: Demyristoylation of H4K5Myr (**5**) and H4K5MyrMe (**6**) by SIRT2, SIRT3 and SIRT6. **A**. HPLC chromatograms from demyristoylation reactions by SIRT2. **B.** HPLC chromatograms from demyristoylation reactions by SIRT3. **C.** HPLC chromatograms from demyristoylation reactions by SIRT6. 100 µM of peptide **5** (H4K5Myr) (black, red, purple) or **6** (H4K5MyrMe) (green, blue, purple) were incubated with the respective sirtuin (black, green, purple) in the presence (black, green) or absence of NAD^+ (purple) or alone (red, blue) for 24 h at 30 °C. The omission of NAD^+ in the reaction mixture as a control resulted in prevention of the reaction (purple). Analyses of the samples were performed by RP-HPLC using a C18 column and UV detection at 280 nm. The demyristoylation of peptides caused elution at an earlier retention.

D. Crystallographic data

Table S2: Crystallographic data collection and refinement statistics of the SIRT2–**12** and SIRT2–**12**–NAD+ complexes.

Structure	**zSIRT5–KSucMeCPS1**	**SIRT2–12**	**SIRT2–12–NAD+**
Beamline	BESSY Beamline 14.1	ID30A-3	ID23-2
**Data Collection**			
Space group	$P6_522$	$P2_1$	$P2_1$
a, b, c (Å)	87.43, 87.43, 314.84	36.11, 73.12, 55.02	38.01, 75.93, 56.30
α, β, γ (deg)		90.00, 95.49, 90.00	90.00, 97.28, 90.00
Wavelength (Å)	0.9184	0.9677	0.8731
Resolution (Å)	19.69 – 2.80 (2.90 – 2.80)	43.83 – 1.45 (1.47 – 1.45)	44.99 – 2.80 (2.95 – 2.80)
Total/unique no. of reflections	345,507 (18,451)	353,686 (49,798)	42,350 (7,337)
$R_{merge}{}^{a,b}$	0.258 (2.381)	0.071 (0.437)	0.439 (3.396)
$R_{pim}{}^{a,c}$	0.061 (0.567)	0.029 (0.172)	0.225 (1.661)
$CC_{1/2}{}^{a,d}$	0.995 (0.494)	0.998 (0.938)	0.789 (0.207)
$I/\sigma(I)$ [a]	12.3 (1.6)	16.5 (4.8)	6.0 (1.2)
Redundancy[a]	18.7 (18.5)	7.1 (7.3)	5.8 (5.9)
Completeness (%)[a]	99.6 (100.0)	98.8 (99.0)	93.1 (99.1)
**Refinement**			
No. of reflections used in refinement/test set	17,526 (1,227)	49,769 (4,982)	7,137 (713)
$R_{work}{}^{e}$	0.199 (0.337)	0.174 (0.221)	0.228 (0.333)
$R_{free}{}^{f}$	0.262 (0.389)	0.199 (0.252)	0.264 (0.385)
Number of Atoms[g]	4275	2745	2249
Average *B*-Factors (Å^2)	68.3	21.1	42.8
protein		19.8	42.8
ligands		37.6	44.0
solvent		28.5	40.2
RMS Deviations			
bonds (Å)	0.011	0.006	0.01
angles (deg)	1.64	0.84	1.19
Ramachandran plot (%)[h]			
favored	93.42	98.25	95.02
allowed	5.23	1.75	4.18
outliers	1.35	0	0.38
PDB accession code	30KU	30KD	30KE

[a] Values in parentheses refer to the highest-resolution shell of the data.

[b] $R_{merge} = \sum|I_h - \langle I_h\rangle|/\sum\langle I_h\rangle$; I_h = intensity measure for reflection *h;* $\langle I_h\rangle$ = average intensity for reflection *h* calculated from replicate data.

[c] $R_{pim} = \sum(1/(n-1))^{1/2}|I_h - \langle I_h\rangle|/\sum\langle I_h\rangle$; *n* = number of observations (redundancy).

[d] $CC_{1/2} = \sigma_\tau^2/(\sigma_\tau^2 + \sigma_\varepsilon^2)$, where σ_τ^2 is the true measurement error variance and σ_ε^2 is the independent measurement error variance.

[e] $R_{work} = \sum||F_o| - |F_c||/\sum|F_o|$ for reflections contained in the working set. $|F_o|$ and $|F_c|$ are the observed and calculated structure factor amplitudes, respectively.

[f] $R_{free} = \sum||F_o| - |F_c||/\sum|F_o|$ for reflections contained in the test set held aside during refinement.

[g] Per asymmetric unit.

[h] Assessed by MolProbity.

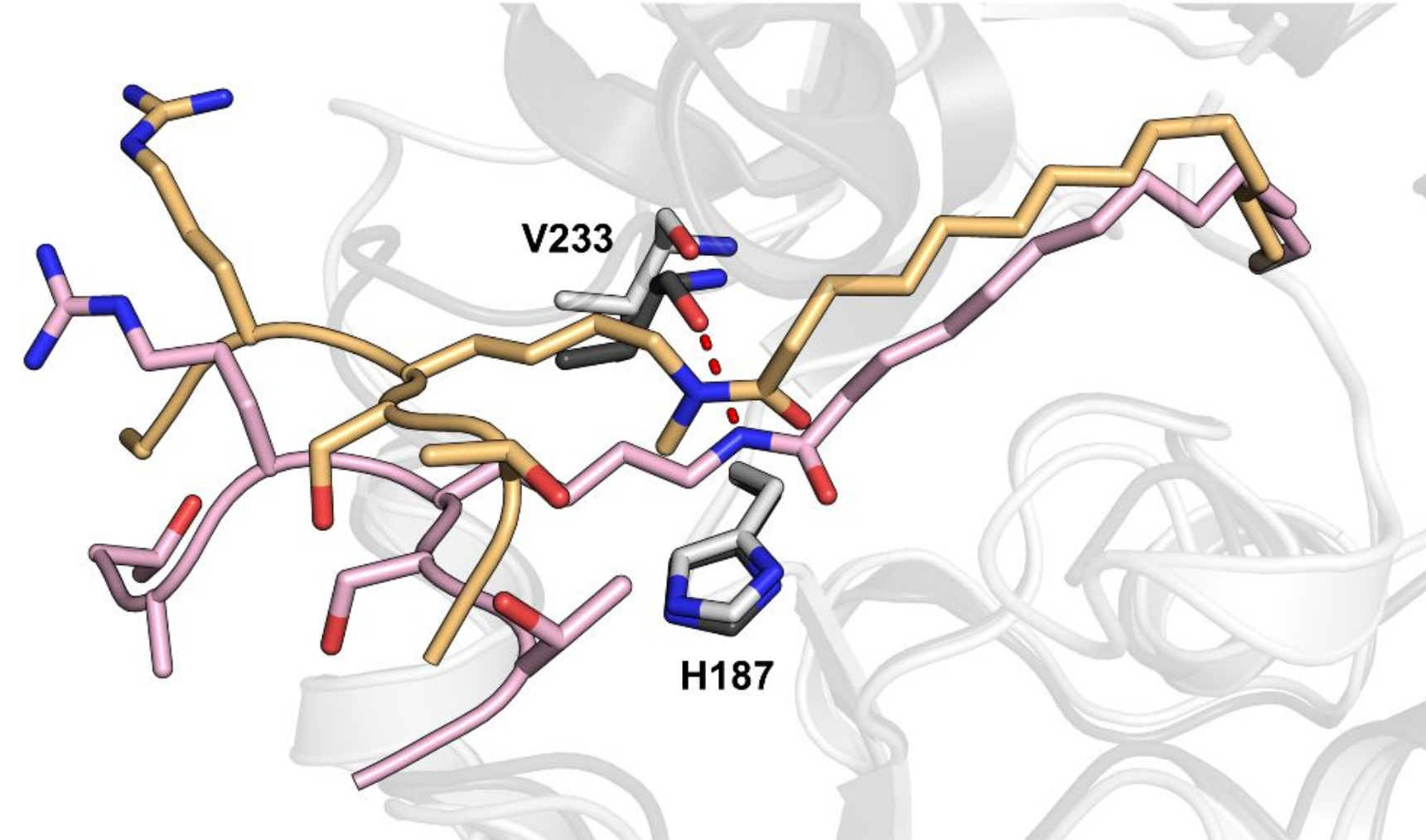

Figure S42: Structural analysis of the SIRT2–**12** (PDB 30KD) complex. Superimposition of SIRT2–**12** (yellow–orange) with SIRT2–H3K9Myr (light pink, PDB 4Y6L). To avoid steric clashes with the Val233 backbone, the N-methyl amide bond of KMyrMe in the SIRT2–**12** (PDB 30KD) complex adopts a *cis* conformation. In contrast, the amide bond of KMyr in the SIRT2–H3K9Myr complex adopts a *trans* conformation and the amide nitrogen forms a hydrogen bond with the backbone carbonyl of Val233.
